# How Are Adolescents Participating in the Transformation of Healthy Food Environments? A Scoping Review of Empirical Research

**DOI:** 10.1111/obr.70002

**Published:** 2025-07-24

**Authors:** Allyson R. Todd, Putu Novi Arfirsta Dharmayani, Sisi Jia, Rebecca Raeside, Seema Mihrshahi, Katrina E. Champion, Health Advisory Panel for Youth at the University of Sydney (HAPYUS), Penny Farrell, Alice A. Gibson, Stephanie R. Partridge

**Affiliations:** ^1^ Susan Wakil School of Nursing and Midwifery, Faculty of Medicine and Health The University of Sydney Camperdown New South Wales Australia; ^2^ Charles Perkins Centre The University of Sydney Camperdown New South Wales Australia; ^3^ Department of Health Sciences, Faculty of Medicine, Health, and Human Sciences Macquarie University Sydney New South Wales Australia; ^4^ School of Public Health, Faculty of Medicine and Health The University of Sydney Camperdown New South Wales Australia; ^5^ The Matilda Centre, Faculty of Medicine and Health The University of Sydney Camperdown New South Wales Australia; ^6^ The Leeder Centre for Health Policy, Economics & Data, Faculty of Medicine and Health The University of Sydney Camperdown New South Wales Australia

**Keywords:** adolescent participation, food environments, noncommunicable disease prevention, nutrition policy, public health nutrition, youth engagement

## Abstract

Adolescence (10–19 years) is a pivotal life stage, presenting both risks and opportunities for optimizing nutrition. There are global calls advocating for adolescents to play a central role in transforming food environments. The extent to which adolescents have participated in research to improve food environments is unknown. A scoping review was conducted to investigate the extent, impact, and processes of adolescent participation in food environment research. Included studies were mapped through the Healthy Food Environment Policy Index. Extent, impact (individual/community/national/international‐level), and processes (barriers/enablers) were analyzed. Adolescent co‐researchers informed each stage. Eighty‐nine articles (70 unique studies with 20,697 participants across 31 countries) were identified. Most (81%) were conducted in high‐income countries. Food retail (56%) and provision (54%) were most reported, improving the availability of healthy food in their local community and school. Most were adolescent‐led (41%); however, only 16% included adolescents throughout each research stage. Impact included improved professional skills and implemented policy. Using engaging participatory methods helped reduce power imbalances. There is substantial evidence of adolescents participating in various modes, methods, and levels of research to transform healthy food environments through policy, demonstrating adolescents can be central actors for change. Ensuring diverse representation from priority population groups and lower‐income countries is necessary to reduce the global noncommunicable disease burden.

## 1 Introduction

Noncommunicable diseases (NCDs) are among the most significant health challenges facing today's 1.3 billion adolescents aged 10–19 years worldwide [1]. With the largest adolescent population in history [1], it is important to support their health as they transition into adulthood. Adolescence is a unique period of growth, accompanied by increased independence, agency, and autonomy [2]. This pivotal life stage presents both risks and opportunities for health through optimal nutrition with lasting intergenerational impacts [1, 2, 3]. Optimal nutrition is one of the most important protective factors to prevent NCDs among adolescents [4]. Currently, poor diets contribute to one in five (22%) adult deaths from NCDs globally [4]. Further, many countries are experiencing a triple burden of malnutrition, where rising rates of overweight and obesity are co‐existing with undernutrition and micro‐nutrient deficiencies [5, 6, 7]. Globally, 159 million children and adolescents are living with obesity which increases their risk of co‐morbidities in adulthood such as cardiovascular disease, diabetes type 2, and some cancers [4, 8]. National data on adolescents' dietary intake are limited, particularly within low and middle‐income countries (LMICs) [2, 9, 10]. From available data, it is estimated that 40% of populations aged over 5 years globally do not meet the recommendations outlined in their national dietary intake guidelines [11]. Most adolescents do not meet their daily recommended intake of fruits and vegetables. This is coupled with high consumption of energy‐dense, nutrient‐poor processed foods and sugar sweetened beverages, categorized as “unhealthy foods and drinks” [6, 12]. Achieving the sustainable development goals (SDGs), including Goals 2 and 3 to reach Zero Hunger and Good Health and Wellbeing is paramount to optimizing nutrition and improving adolescent health and wellbeing [13, 14, 15].

The food environment is defined by an array of physical, political, economic, socio‐cultural factors that influence people's diet and nutritional status [16, 17]. Collectively, these factors determine how consumers interact with the food system [2]. Current food environments do not promote healthy dietary choices for adolescents due to the convenience, availability, affordability, and advertising of unhealthy food and drinks [2]. Adolescents living in areas of low socio‐economic status, or in rural or regional communities, commonly experience “food deserts” where there is a paucity of healthy foods available in comparison to unhealthy foods, influencing poorer health outcomes [18]. Further, adolescents are often exposed to marketing of unhealthy foods and drinks near their schools, sporting events, public transport, and media platforms [19, 20, 21]. Addressing these upstream determinants of health are necessary to promote healthy food environments that optimize adolescent nutrition and reduce the global NCD burden.

The World Health Organization (WHO) and the United Nations Children Fund (UNICEF) are advocating for adolescents to play a central role in transforming food systems to ensure accessible, sustainable, and optimal nutrition for all [22]. Adolescents have a right to adequate nutrition and to be involved in decisions impacting them, as outlined in the 1989 Convention on the Rights of the Child that has been ratified by 196 countries [23]. Meaningful adolescent participation upholds these rights, enabling adolescents to be central actors in driving positive change on local and global scales [2, 10, 24].

Although adolescents' preferences to be involved in food environment transformation may vary across populations, adolescents want to engage at various levels [25, 26]. This can range from being consulted on policy decisions impacting them or being collaborative partners, through to adolescent‐led initiatives such as advocacy [17, 24]. Globally, adolescents are advocating to be involved in improving food environments [27]. For example, they are demanding greater regulation to reduce the harmful marketing from fast food outlets [27, 28, 29] and improving the availability and affordability of nutritious food [27]. There are global calls urging the importance of creating opportunities for adolescents to meaningfully contribute to solutions to improve food environment by sharing their needs, ideas, and perspectives [2, 10, 27]. Actively involving adolescents throughout policy‐relevant research is needed to recognize the dynamic, social, and cultural contexts of how adolescents interact with their food environment in their home, school, and local community [2, 30, 31]. It is also reported that adolescent involvement in policy processes may lead to individual benefits including increased wellbeing and self‐efficacy [26, 32, 33]. This is important because expecting adolescents to participate from altruistic motivation alone may lead to self‐selection bias and limit inclusive participation. Further, it has the potential to enhance the quality and value of adolescent‐focused research. Ultimately, these benefits may be a key factor in building healthy food environment policies [2, 27, 34].

Food environment policies have the potential to improve nutritional outcomes and reduce socio‐economic health inequities for adolescents and future generations to come [2, 35]. For example, over 50 countries have introduced a tax on sugar sweetened beverages [36]. In Mexico, this has led to reduced sugar consumption, particularly across low income households, as well as influenced sugar sweetened beverage reformation to reduce sugar levels [37, 38]. It is also estimated to be cost‐effective [36]. For every USD $1 spent on implementation of this tax in Mexico, it leads to a USD $4 return in health care costs [39], highlighting the potential for food environment policies to reduce the NCD burden.

Research‐informed policy is required to promote healthier, supportive food environments. The Healthy Food Environment Policy Index (Food‐EPI) tool was developed by the International Network for Food and Obesity/noncommunicable diseases (NCDs) Research, Monitoring and Action Support (INFORMAS) to assess and monitor the implementation of food policies worldwide based on international best practice [40]. They developed seven policy domains: (i) food composition, (ii) labeling, (iii) promotion, (iv) provision, (v) pricing, (vi) retail, and (vii) trade and investment. Research that is aligned with these policy domains has the potential to inform policy decision makers to improve healthier food environments.

There are ongoing calls to promote opportunities for adolescents to meaningfully contribute to solutions to improve food environments [2, 22]. However, it is unknown to what extent adolescents have participated in research focused on improving food environment policies that impact them. Therefore, the primary aim of this study was to conduct a scoping review to investigate the extent of adolescent participation in transforming healthy food environments in empirical studies, mapped through the Food‐EPI policy domains. A secondary aim was to understand the impact of participation on adolescents individually and any reported policy impacts on a community, national, or international level. Further, we aimed to understand the processes that enable or inhibit participation from adolescent and adult perspectives in food environment policy research.

## 2 Materials and Methods

### 2.1 Protocol and Registration

Our review was informed by the Joanna Briggs Institute scoping review guidelines [41, 42, 43], Arksey and O'Malley's [44] six‐stage methodological framework, and Levac and colleagues recommendations for scoping reviews [45]. Further, our review adheres to the Preferred Reporting Items for Systematic Reviews and Meta‐Analyses extension for Scoping Reviews (PRISMA‐ScR) [46] The PRISMA‐ScR checklist is available in the Supporting Information. Quality assessment was not conducted due to the explorative nature of this review [41]. Detailed methodology is available in the pre‐registered scoping review protocol on Open Science Framework, doi: https://osf.io/jazd3.

We consulted adolescents (aged 16–18 years) at each stage of our scoping review and incorporated their suggestions. Adolescents were members of the Health Advisory Panel for Youth at The University of Sydney (HAPYUS) and co‐authored this manuscript as co‐researchers (Table 1). Scoping review guidelines recommend consultation to inform and validate research findings [41, 42]. Ethical approval was not required as they were not involved as traditional study participants. None of the adolescents' personal health data or demographic data were collected or reported as part of this scoping review.

**TABLE 1:** Definitions of adolescent participation.

Mode of adolescent participation	
Consultative	Adult initiated and led where adolescents contribute their ideas, perspectives, knowledge, and experience, often as traditional study participants, for example, through surveys and focus groups.
Collaborative	Adult initiated; however, adolescents are involved as partners and able to influence the decision‐making processes. This may include engaging as a co‐researcher.
Adolescent‐led	Adolescents identify the topic and control the process and outcomes, often with the assistance of adult facilitators. This includes leading an initiative or research project.

Research cycle stages	
Identification of topic	Adolescents' views, opinions, or aspirations are sought in the identification and development of the research idea and or topic.
Design and development	Adolescents are involved in the selection and development of methods.
Conduct	Adolescents lead or facilitate research methods and collect data.
Analyses	Adolescents are involved in consolidating and reporting findings.
Dissemination	Adolescents contribute to sharing and presenting the findings. They may also assist translating the results into action.
Adolescent co‐researcher	Adolescents who make a significant contribution to any stage of the research cycle. Personal data or information are not collected for analysis.
Traditional study participant	An individual who voluntary takes part in a research study by providing personal data or information for the researcher to analyze.

*Note:* Definitions are adapted from the UNICEF Conceptual Framework for Measuring Outcomes of Adolescent Participation [24], the Australia's National Health and Medical Research Council (NHMRC) Framework for Effective Consumer and Community Engagement in Research [47] and the NHMRC National Statement on Ethical Conduct in Human Research [48].

### 2.2 Eligibility Criteria

Peer reviewed empirical studies were eligible for inclusion if they outlined how adolescents participated to improve healthy food environments across any of the seven Food‐EPI policy domains: (i) food composition, (ii) labeling, (iii) promotion, (iv) provision, (v) pricing, (vi) retail, and (vii) trade and investment [40]. Adolescence was defined as the second decade of life (10–19 years inclusive) corresponding with secondary school [1], and participants could be of any gender and from any country [49]. Adolescent participation was defined according to the Lansdown‐UNICEF conceptual framework's [24] mode of participation, which may range from consultative, collaborative, or adolescent‐led (Table 1) as previously used in other reviews [26, 50]. Studies that only involved adolescents as traditional study participants were eligible if they were consulted or had a say in a Food‐EPI policy domain impacting them [40]. No restriction was placed on publication date or language. A translation software was used as required. Studies were excluded if the mean age was outside 10–19 years and where adolescents did not have the opportunity to express their perspectives or contribute to research focused on a Food‐EPI policy domain [40].

### 2.3 Information Sources and Search Strategy

A search strategy was developed with the assistance of an academic librarian and informed by relevant literature [26, 51, 52]. Six databases were searched: MEDLINE (via OVID), Embase (via OVID), Eric (via OVID), Cinahl (via EBSCO), Scopus, and Web of Science (Web of Science Core Collection) capturing studies published before May 6, 2024. The following search term categories, combinations, truncations, and synonyms were used: “adolescent participation” OR “youth engagement” OR “co‐design” AND “food environment policy” OR “food retail” OR “food provision” OR “food marketing” OR “food accessibility” OR “food labelling” OR “nutritional quality” OR “food price” OR “food trade and investment.” The search strategy for MEDLINE is available in the Supporting Information.

### 2.4 Evidence Selection

All identified studies were collated and imported into Covidence Software (Veritas Health Innovation, Melbourne, Australia). After duplicates were removed, all abstracts and full texts were independently dual screened (A.R.T., P.N.A.D., S.J., P.F., A.A.G., and S.R.P.) against the eligibility criteria. Discrepancies were discussed with the research team until consensus was reached. Any remaining conflicts were resolved by a third reviewer (SRP). Corresponding authors were emailed to obtain missing or unreported data that were necessary for inclusion. Reference lists of reviews and included studies were scanned for citation chaining.

### 2.5 Data Charting

A data extraction template was developed by the lead author (ART) to capture key outcome data with variables encompassing the extent, impact, and process of adolescent participation in research to inform healthy food environment policies. Extent outcomes extracted included: study and socio‐demographic characteristics; modes and methods of participation; level of research cycle involvement; and youth engagement frameworks used. The impact of adolescent participation was extracted on the individual level, including outcomes on health and well‐being or self‐development. Further, we extracted any policy‐related impacts that were focused on healthy food environments and categorized this across the community, national, and international levels. Policy‐related impact was defined broadly to capture short‐ or long‐term outputs that may shape, inform, or influence healthy food environment policies. Process outcomes extracted were barriers and enablers to adolescent participation from the perspective of the adolescents and adult researchers if reported. Data extraction was performed by ART, with 20% cross‐checked for consistency by two master's students (available in the Supporting Information).

### 2.6 Synthesis of Results

Adolescent participation was mapped across the Food‐EPI policy domains [40]. The extent of participation was assessed according to the key modes of participation. It was also contextualized throughout the stages of the research cycle, informed by Australia's National Health and Medical Research Council (NHMRC) Framework for Effective Consumer and Community Engagement in Research (Table 1) [47]. Further, adolescent engagement frameworks, models, or theories were categorized as power, process, impact, or equity focused to inform analysis [53]. Simple descriptive statistics were calculated to highlight the extent of participation and are presented in tables. Content analyses were performed through an inductive approach to identify recurring concepts of impact and processes of participation and are reported in narrative synthesis [54].

## 3 Results

**FIGURE 1:**
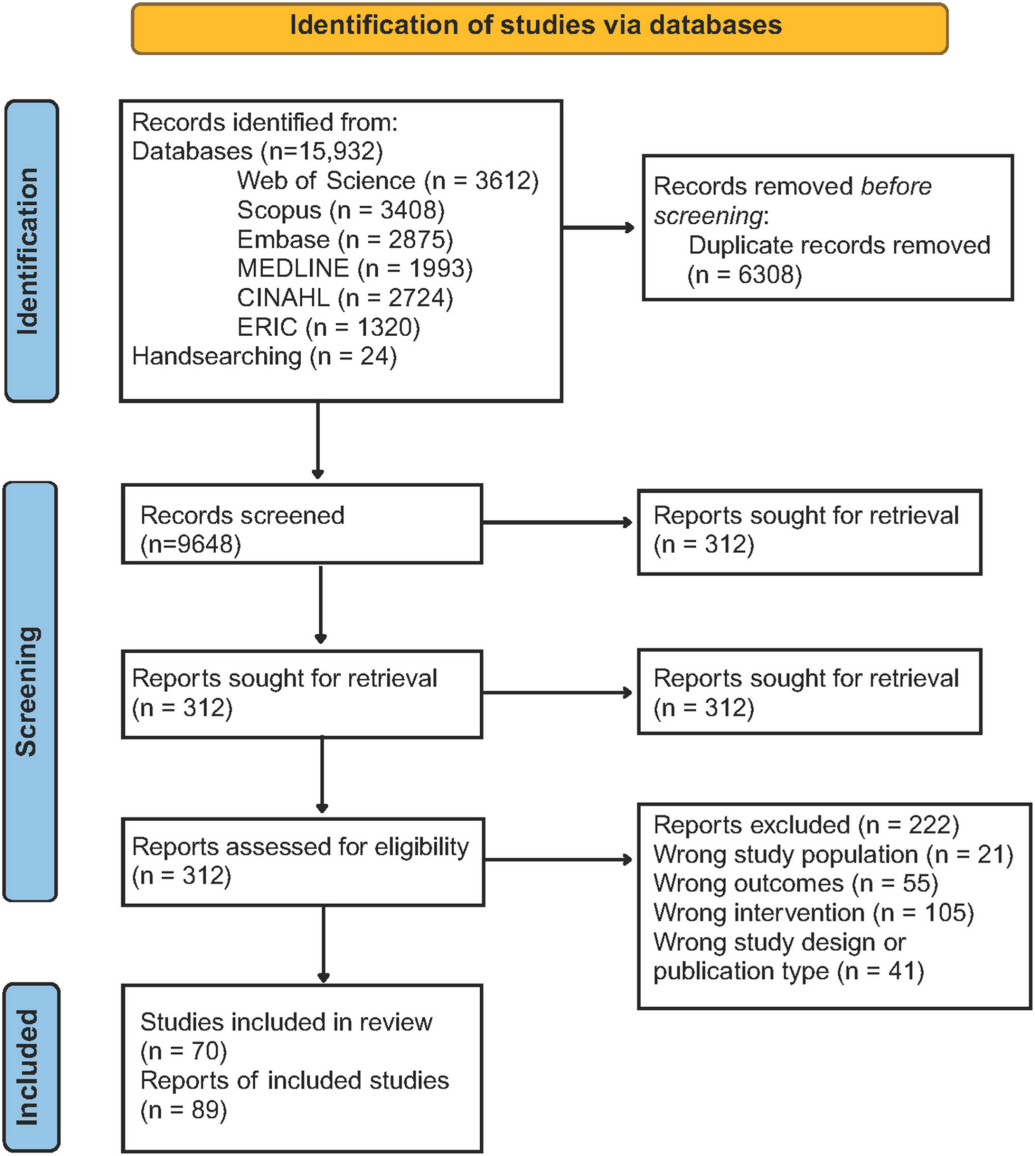
PRISMA‐ScR flow diagram.

The initial search strategy produced 15,932 articles (Figure 1). Twenty‐four studies were identified through handsearching. After duplicates were removed, 9648 studies were screened against the eligibility criteria. A total of 312 full texts were dual assessed by two independent reviewers. Reasons for exclusion are available in Supporting Information. Seventy unique studies were included across 89 articles [55, 56, 57, 58, 59, 60, 61, 62, 63, 64, 65, 66, 67, 68, 69, 70, 71, 72, 73, 74, 75, 76, 77, 78, 79, 80, 81, 82, 83, 84, 85, 86, 87, 88, 89, 90, 91, 92, 93, 94, 95, 96, 97, 98, 99, 100, 101, 102, 103, 104, 105, 106, 107, 108, 109, 110, 111, 112, 113, 114, 115, 116, 117, 118, 119, 120, 121, 122, 123, 124, 125, 126, 127, 128, 129, 130, 131, 132, 133, 134, 135, 136, 137, 138, 139, 140, 141, 142, 143] representing 20,697 unique adolescents across 31 countries.

### 3.1 Study Characteristics

Studies were published from 1996 to 2024, with most published within the last 10 years (79%), peaking in 2023 (17%) (Table 2). Most studies (59%) were of qualitative design [55, 56, 58, 62, 64, 65, 66, 67, 68, 72, 76, 80, 85, 90, 91, 92, 94, 96, 97, 100, 102, 103, 105, 106, 109, 115, 117, 118, 121, 122, 124, 128, 129, 130, 131, 132, 133, 136, 141, 143] and were conducted in North America (61%) [55, 56, 57, 58, 59, 63, 64, 65, 66, 67, 74, 77, 78, 80, 81, 83, 84, 85, 91, 93, 94, 96, 97, 99, 100, 103, 105, 106, 109, 111, 113, 118, 119, 120, 121, 125, 130, 133, 135, 141, 142]. Most studies (81%) were based in high‐income countries [55, 56, 57, 58, 59, 60, 62, 63, 64, 65, 66, 67, 68, 73, 74, 75, 76, 77, 78, 79, 80, 81, 83, 84, 85, 89, 90, 91, 92, 93, 94, 96, 97, 99, 100, 101, 102, 103, 105, 106, 107, 108, 109, 111, 113, 114, 116, 117, 119, 120, 121, 124, 125, 127, 128, 129, 130, 132, 133, 135, 136, 141, 142, 143]. One article (1%) included multiple studies conducted across multiple countries, including LMICs from South Asia, the Middle East, and North Africa [117]. Approximately a third of studies reported equal gender of participants (33%) [59, 63, 65, 77, 79, 80, 84, 85, 89, 92, 96, 100, 101, 103, 104, 105, 106, 108, 109, 111, 117, 128, 142], unreported gender (33%) [56, 57, 60, 62, 64, 67, 72, 74, 75, 90, 91, 94, 99, 114, 115, 116, 123, 124, 129, 130, 135, 136], or had a majority of participants as female (31%) [55, 58, 66, 68, 69, 76, 78, 83, 93, 97, 102, 107, 113, 118, 119, 120, 121, 122, 125, 131, 132, 141], with two studies (3%) only including people from one gender [127, 133]. Five studies (7%) reported including gender‐diverse or non‐binary participants [63, 115, 117, 118, 139].

**TABLE 2:** Detailed characteristics of included studies (*n* = 70).

Study characteristics	Subcategory of study characteristics	Number of results (%)	References
Study design^a^	Qualitative study	41/70 (60%)	[55, 56, 58, 62, 64, 65, 66, 67, 68, 72, 76, 80, 85, 90, 91, 92, 94, 96, 97, 100, 102, 103, 105, 106, 109, 115, 117, 118, 121, 122, 124, 128, 129, 130, 131, 132, 133, 136, 141, 143]
	Mixed methods	21/70 (30.0%)	[57, 59, 63, 74, 77, 78, 81, 93, 99, 101, 104, 108, 111, 113, 119, 120, 123, 125, 127, 135, 142]
	Cross sectional	4/70 (5.7%)	[75, 79, 84, 89]
	Other^b^	4/70 (4.3%)	[61, 107, 114, 116]
Publication year^a^	≤ 2000	1/70 (1.4%)	[105]
	2001–2010	6/70 (8.6%)	[57, 59, 60, 83, 125, 130]
	2011–2015	14/70 (20.0%)	[55, 56, 58, 63, 64, 67, 69, 74, 81, 92, 93, 101, 109, 114, 118]
	2016–2020	26/70 (37.1%)	[65, 66, 77, 78, 79, 84, 85, 90, 96, 99, 100, 103, 106, 108, 113, 115, 116, 121, 122, 128, 131, 133, 135, 136, 142, 143]
	2021–2024	23/70 (32.9%)	[62, 68, 72, 75, 76, 80, 89, 91, 94, 102, 104, 107, 110, 111, 117, 120, 121, 123, 124, 127, 129, 132, 141]
	Range	1996–2024	
Sample size	1–10	6/70 (8.6%)	[56, 66, 74, 109, 118, 133]
	11–50	32/70 (45.7%)	[55, 58, 65, 68, 78, 81, 83, 85, 90, 91, 94, 96, 97, 99, 102, 105, 108, 111, 113, 121, 122, 123, 125, 127, 131, 132]
	51–100	10/70 (14.3%)	[67, 76, 77, 80, 104, 106, 120, 128, 142, 143]
	> 100	20/70 (28.6%)	[57, 59, 60, 63, 64, 72, 75, 79, 84, 89, 93, 101, 107, 115, 116, 119, 124, 129]
	Not reported	2/70 (1.4%)	[62, 130]
	Total participants	20,697	
	Median (IQR)	40 (19.8–183.0)	
	Range	6–9102	
Geographic region^c^	North America	43/70 (61.4%)	[55, 56, 57, 58, 59, 63, 64, 65, 66, 67, 74, 77, 78, 80, 81, 83, 84, 85, 91, 93, 94, 96, 97, 99, 100, 103, 105, 106, 109, 111, 113, 118, 119, 120, 121, 125, 130, 133, 135, 141, 142]
	Europe and Central Asia	40/70 (57.1%)	[62, 68, 73, 75, 76, 90, 101, 102, 107, 108, 117, 124, 127, 128, 129, 132, 136, 143]
	East Asia and Pacific	8/70 (11.4%)	[60, 79, 89, 116, 117, 122, 123]
	Latin America and Caribbean	6/70 (8.6%)	[104, 115, 117, 122, 123]
	Sub‐Saharan Africa	5/70 (7.1%)	[73, 117]

	South Asia	2/70 (2.9%)	[117]
Middle East and North Africa	2/70 (2.9%)	[117]
Countries by World Bank classification of income^c^	High‐income	86/106 (81.1%)	[55, 56, 57, 58, 59, 60, 62, 63, 64, 65, 66, 67, 68, 73, 74, 75, 76, 77, 78, 79, 80, 81, 83, 84, 85, 89, 90, 91, 92, 93, 94, 96, 97, 99, 100, 101, 102, 103, 105, 106, 107, 108, 109, 111, 113, 114, 116, 117, 119, 120, 121, 124, 125, 127, 128, 129, 130, 132, 133, 135, 136, 141, 142, 143]
	Upper middle‐income	9/106 (8.5%)	[104, 115, 117, 122, 123]
	Lower middle‐income	10/106 (9.4%)	[117]
	Low‐income	1/106 (0.9%)	[117]
Gender of participants	Mixed (almost equal proportions of male and female)	23/70 (32.9%)	[59, 63, 65, 77, 79, 80, 84, 85, 89, 92, 96, 100, 101, 103, 104, 105, 106, 108, 109, 111, 117, 128, 142]
	Majority female (≥ 60%)	22/70 (31.4%)	[55, 58, 66, 68, 69, 76, 78, 83, 93, 97, 102, 107, 113, 118, 119, 120, 121, 122, 125, 131, 132, 141]
	All female	1/70 (1.4%)	[133]
	All male	1/70 (1.4%)	[127]
	Reported gender diverse/non‐binary	5/70 (7·1%)	[65, 117, 119, 120, 141]
	Not reported	23/70 (32.9%)	[56, 57, 60, 62, 64, 67, 72, 74, 75, 90, 91, 94, 99, 114, 115, 116, 123, 124, 129, 130, 135, 136]
Age inclusion	10‐year‐old	8/70 (11.4%)	[60, 78, 79, 103, 104, 106, 115, 117, 138]
	11‐year‐old	16/70 (22.9%)	[58, 60, 64, 78, 79, 84, 93, 96, 102, 103, 104, 106, 115, 117, 122, 138]
	12‐year‐old	23/70 (32.9%)	[58, 60, 64, 68, 76, 78, 79, 80, 84, 89, 96, 97, 102, 103, 104, 106, 111, 115, 117, 121, 122, 138]
	13‐year‐old	33/70 (47.1%)	[56, 58, 60, 64, 65, 68, 76, 77, 78, 79, 80, 83, 84, 89, 90, 93, 96, 97, 99, 100, 102, 103, 104, 106, 108, 111, 113, 115, 117, 119, 120, 121, 122]
	14‐year‐old	36/70 (51.4%)	[56, 58, 62, 63, 64, 65, 68, 74, 76, 77, 79, 80, 83, 84, 89, 90, 91, 92, 96, 97, 99, 100, 102, 104, 106, 108, 111, 113, 115, 117, 119, 120, 121, 122, 123]
	15‐year‐old	36/70 (51.4%)	[56, 58, 62, 63, 64, 65, 68, 70, 74, 77, 79, 80, 83, 84, 89, 90, 91, 92, 93, 97, 99, 100, 106, 107, 108, 109, 111, 113, 117, 118, 119, 120, 123, 127, 129, 141]
	16‐year‐old	42/70 (60.0%)	[56, 58, 62, 63, 64, 65, 68, 70, 74, 77, 79, 80, 83, 89, 90, 91, 92, 93, 97, 99, 100, 106, 107, 108, 109, 111, 117, 118, 119, 120, 123, 127, 129, 141]
	17‐year‐old	36/70 (51.4%)	[58, 62, 63, 64, 65, 68, 69, 73, 74, 75, 79, 80, 83, 85, 89, 90, 91, 92, 93, 99, 100, 106, 107, 109, 111, 117, 118, 119, 120, 123, 124, 127, 129, 131, 132]

	18‐year‐old	23/70 (32.9%)	[62, 63, 64, 65, 68, 73, 74, 75, 79, 80, 85, 91, 92, 93, 99, 106, 107, 111, 117, 118, 124, 129, 132]
	19‐year‐old	5/70 (7.1%)	[74, 79, 80, 85, 106, 117]
Age not reported^d^	16/70 (22.9%)	[55, 57, 59, 66, 67, 94, 101, 105, 114, 125, 128, 130, 133, 135, 142, 143]
FOOD‐EPI policy domain addressed^e^	Food retail	39/70 (55.7%)	[55, 56, 58, 63, 64, 65, 67, 68, 72, 74, 76, 77, 80, 81, 84, 85, 92, 93, 96, 99, 100, 103, 106, 107, 109, 111, 113, 117, 118, 123, 124, 128, 129, 130, 131, 132, 141, 142]
	Food provision	38/70 (54.3%)	[55, 57, 58, 59, 60, 63, 64, 66, 75, 76, 80, 83, 84, 89, 90, 91, 93, 97, 99, 100, 101, 105, 106, 108, 114, 116, 122, 124, 125, 128, 129, 131, 132, 133, 136, 141, 143]
	Food promotion	24/70 (34.3%)	[58, 62, 68, 72, 76, 81, 83, 90, 94, 96, 100, 102, 104, 107, 113, 119, 120, 121, 124, 128, 129, 130, 131, 132]
	Food prices	17/70 (24.3%)	[58, 62, 64, 72, 89, 96, 100, 102, 117, 124, 129, 130, 132, 133]
	Food labelling	11/70 (15.7%)	[64, 68, 79, 89, 102, 104, 114, 115, 121, 127, 132]
	Food composition	2/70 (2.9%)	[96, 127]
	Food trade and investment	1/70 (1.4%)	[130]
	Addressed ≥ 2 Food‐EPI policy domains	36/70 (51.4%)	[55, 56, 58, 62, 63, 64, 68, 70, 72, 73, 76, 80, 81, 83, 84, 89, 90, 93, 96, 99, 100, 102, 104, 106, 107, 113, 117, 121, 124, 127, 128, 129, 130, 131, 132, 133, 141]
	Total food‐EPI policy domain outcomes	132	
Socioeconomic status (SES)	Low SES	6/70 (8.6%)	[56, 69, 89, 99, 131, 135]
	Underserved/low‐income setting; however, participant data are not reported	18/70 (25.7%)	[64, 67, 74, 78, 85, 91, 94, 96, 104, 111, 113, 114, 118, 122, 123, 133, 138, 141]
	Mixed high and low SES	9/70 (12.9%)	[68, 71, 79, 80, 83, 90, 101, 105, 108]
	High SES	1/70 (1.4%)	[100]
	Not reported	36/70 (51.4%)	[55, 57, 58, 59, 60, 62, 63, 65, 66, 73, 75, 76, 77, 84, 92, 93, 97, 102, 103, 106, 107, 109, 115, 117, 119, 120, 121, 124, 125, 127, 128, 129, 130, 132, 142, 143]

^a^ Study design and year of first published paper if there were multiple reports published regarding the same study.

^b^ One study was a pre‐post study, one a concurrent controlled before‐and‐after study, and another a quasi‐experimental study.

^c^ Total count is greater than the number of included studies as six studies report more than one country.

^d^ Studies reported that the adolescents participating were in high school.

^e^ Total count is greater than included studies as most addressed more than one policy outcome.

Age was inconsistently reported across studies. All studies included participants in secondary school, and more than half included participants aged between 14 and 17 years [56, 58, 62, 63, 64, 65, 68, 69, 70, 73, 74, 75, 76, 77, 79, 80, 83, 84, 85, 89, 90, 91, 92, 93, 96, 97, 99, 100, 102, 104, 106, 107, 108, 109, 111, 113, 115, 117, 118, 119, 120, 121, 122, 123, 124, 127, 129, 131, 132, 141]. Where reported, participants across studies were aged between 9 and 24 years, yet as per our inclusion criteria, the reported mean age for all studies was between 10 and 19 years.

Approximately half of the studies (51%) did not report the socio‐economic status (SES) of participants [55, 57, 58, 59, 60, 62, 63, 65, 66, 73, 75, 76, 77, 84, 92, 93, 97, 102, 103, 106, 107, 109, 115, 117, 119, 120, 121, 124, 125, 127, 128, 129, 130, 132, 142, 143]. However, 26% reported that the study was conducted in an underserved or low‐income area [64, 67, 74, 78, 85, 91, 94, 96, 104, 111, 113, 114, 118, 122, 123, 133, 138, 141]. Nine studies (13%) included participants from both high and low SES [68, 71, 79, 80, 83, 90, 101, 105, 108], six studies (9%) included only participants with a low SES [56, 69, 89, 99, 131, 135], and only one study (1%) included participants with a high SES [100].

### 3.2 Study Outcomes: Extent

#### 3.2.1 Food Environment Policy

Overall, there were 132 Food‐EPI policy‐related outcomes. Thirty‐six studies (51%) [55, 56, 58, 62, 63, 64, 68, 70, 72, 73, 76, 80, 81, 83, 84, 89, 90, 93, 96, 99, 100, 102, 104, 106, 107, 113, 117, 121, 124, 127, 128, 129, 130, 131, 132, 133, 141] reported more than one policy outcome. *Food retail* was the most common policy domain addressed across 39 studies (56%) [55, 56, 58, 63, 64, 65, 67, 68, 72, 74, 76, 77, 80, 81, 84, 85, 92, 93, 96, 99, 100, 103, 106, 107, 109, 111, 113, 117, 118, 123, 124, 128, 129, 130, 131, 132, 141, 142]. Most of these studies focused on improving the availability of healthy food in their local community outside of a school setting to address the higher proportion of unhealthy food sold in retail stores.

*Food provision* was addressed by 38 studies (54%), with most focusing on improving healthy canteen or cafeteria policies within schools. Twenty‐four studies (34%) focused on *food promotion* [58, 62, 68, 72, 76, 81, 83, 90, 94, 96, 100, 102, 104, 107, 113, 119, 120, 121, 124, 128, 129, 130, 131, 132] most commonly on the marketing of unhealthy foods in‐store and online through special offers and discounts and using graphics to entice consumers. *Food prices* were addressed by 17 studies (24%) [58, 62, 64, 72, 89, 96, 100, 102, 117, 124, 129, 130, 132, 133] with most highlighting cost as a barrier to healthy eating or focused on a sugar‐sweetened beverages tax.

*Food labeling* was addressed by 11 studies (16%) [64, 68, 79, 89, 102, 104, 114, 115, 121, 127, 132] with most emphasizing the importance of easy‐to‐understand nutrition labels on food products to help consumers make healthier choices. Two studies [96, 127] reported on *food composition*, focusing on nutritional quality and portion sizes of foods. One study addressed *food trade and investment*, which aimed to reduce the availability of unhealthy food products in corner stores from companies that were subsidized by the tobacco industry [130].

#### 3.2.2 Methods of Adolescent Participation

The most common mode of participation was adolescent‐led (41%), [60, 62, 63, 65, 67, 69, 70, 73, 83, 87, 88, 92, 96, 99, 106, 107, 109, 111, 112, 118, 122, 123, 124, 128, 129, 130, 132, 138, 141, 142] followed by consultative (36%) [55, 57, 58, 59, 64, 68, 75, 76, 79, 84, 89, 90, 91, 94, 100, 101, 102, 105, 108, 115, 117, 121, 125, 135, 143] and collaborative (23%) [56, 66, 74, 77, 78, 80, 85, 97, 103, 104, 113, 119, 120, 127, 131, 133] (Table 3).

**TABLE 3:** Summary of adolescent participation (*n* = 70).

Study characteristics	Subcategory of study characteristics	Number of results (%)	References
Methods of participation^a^	Focus groups	27/70 (38.6%)	[55, 63, 64, 66, 67, 68, 76, 77, 86, 91, 94, 96, 102, 103, 105, 108, 111, 115, 119, 121, 125, 127, 128, 130, 133, 135, 137, 143]
	Surveys	22/70 (31.4%)	[56, 57, 59, 63, 65, 74, 75, 77, 79, 84, 86, 89, 92, 98, 99, 102, 107, 108, 112, 113, 124, 126, 129, 130, 131, 134]
	Photovoice	19/70 (27.1%)	[55, 58, 65, 66, 77, 80, 83, 88, 92, 96, 103, 109, 118, 124, 129, 131, 132, 141, 142]
	Collecting field data^b^	12/70 (17.1%)	[55, 74, 77, 86, 96, 99, 106, 111, 119, 120, 127, 130, 142]
	Interviews	11/70 (15.7%)	[58, 86, 96, 97, 100, 106, 111, 113, 126, 130, 131, 135]
	Youth advisory positions^c^	7/70 (10.0%)	[61, 62, 95, 106, 107, 124, 126]
	Participatory workshops	6/70 (8.6%)	[62, 90, 104, 113, 117, 126]
	Geographic Information System Mapping	6/70 (8.6%)	[56, 74, 78, 85, 123, 131]
	Advocacy	4/70 (5.7%)	[88, 111, 124, 133]
	Social marketing	3/70 (4.3%)	[69, 95, 114]
	System mapping	3/70 (4.3%)	[73, 113, 126]
	Other^d^	6/70 (8.6%)	[63, 67, 70, 90, 122, 141]
Modes of participation	Consultative	25/70 (35.7%)	[55, 57, 58, 59, 64, 68, 75, 76, 79, 84, 89, 90, 91, 94, 100, 101, 102, 105, 108, 115, 117, 121, 125, 135, 143]
	Collaborative	16/70 (22.9%)	[56, 66, 74, 77, 78, 80, 85, 97, 103, 104, 113, 119, 120, 127, 131, 133]
	Adolescent‐led	29/70 (41.4%)	[60, 62, 63, 65, 67, 69, 70, 73, 83, 87, 88, 92, 96, 99, 106, 107, 109, 111, 112, 118, 122, 123, 124, 128, 129, 130, 132, 138, 141, 142]
Levels of research cycle involvement^a^	Identification of topic	24/70 (34.3%)	[60, 61, 63, 65, 70, 71, 83, 86, 87, 88, 92, 93, 99, 106, 107, 109, 116, 117, 118, 122, 124, 128, 129, 130, 131, 132, 133, 136, 137, 138, 139, 140, 141, 142]
	Design or development	28/70 (40.0%)	[60, 61, 62, 63, 65, 69, 81, 82, 83, 86, 87, 88, 93, 94, 95, 97, 99, 100, 106, 107, 109, 111, 112, 113, 114, 120, 122, 124, 128, 129, 130, 133, 136, 137, 138, 139, 140, 142]
	Conduct	45/70 (64.3%)	[55, 56, 58, 60, 61, 62, 63, 66, 69, 70, 71, 72, 73, 74, 77, 78, 80, 81, 82, 83, 85, 86, 87, 88, 90, 92, 93, 96, 97, 103, 106, 107, 111, 112, 113, 114, 116, 117, 118, 119, 123, 124, 128, 129, 130, 131, 132, 133, 136, 137, 138, 139, 140, 141, 142]
	Analyses	22/70 (31.4%)	[62, 63, 65, 66, 72, 73, 80, 82, 83, 85, 86, 92, 99, 113, 118, 123, 128, 129, 130, 132, 133, 136, 137, 138, 139, 140, 141, 142]
	Dissemination	31/70 (44.3%)	[55, 62, 65, 69, 71, 74, 78, 81, 83, 85, 86, 87, 88, 92, 93, 99, 103, 106, 109, 111, 112, 113, 114, 117, 118, 123, 127, 130, 131, 132, 133, 136, 137, 138, 139, 140, 141, 142]
	Participation in at least one of the two formative stages (stages 1 and 2)	34/70 (48.6%)	[60, 61, 62, 63, 65, 69, 81, 82, 83, 86, 87, 88, 93, 94, 95, 97, 99, 100, 104, 106, 107, 109, 111, 112, 114, 119, 120, 122, 124, 128, 129, 130, 133, 136, 137, 138, 139, 140, 141, 142]
	Participation in all 5 stages	11/70 (15.7%)	[63, 65, 83, 86, 99, 109, 129, 130, 133, 136, 137, 138, 139, 140, 141, 142]
	Traditional study participant^e^	40/70 (57.1%)	[55, 57, 58, 59, 60, 61, 63, 64, 67, 68, 75, 76, 77, 79, 84, 85, 89, 91, 92, 94, 95, 96, 97, 100, 101, 102, 105, 106, 107, 108, 111, 113, 115, 120, 121, 123, 125, 127, 128, 131, 135, 143]
Youth engagement frameworks^f^	**Power focused**	3/70 (4.3%)	
	Ladder of authentic youth participation	1/70 (1.4%)	[106]

	Hart's ladder of children's participation	1/70 (1.4%)	[138]
	Type pyramid	1/70 (1.4%)	[65]
	**Process focused**	3/70 (4.3%)	
	Proposed engagement framework	1/70 (1.4%)	[78]
	P7 model	1/70 (1.4%)	[65]
	Patient oriented framework	1/70 (1.4%)	[100]
	**Impact focused**	2/70 (2.9%)	
	Youth Development Aotearoa Strategy goals framework	1/70 (1.4%)	[60]
	Youth engagement conceptual framework	1/70 (1.4%)	[93]
	**Equity focused**	1/70 (1.4%)	[142]
	YPAR 2.0	1/70 (1.4%)	[142]
	**Multi‐focused**	27/70 (38.6%)	
	CBPR	13/70 (18.6%)	[63, 64, 80, 83, 84, 85, 86, 92, 96, 99, 109, 113, 130, 133]
	YPAR	13/70 (18.6%)	[56, 62, 66, 72, 73, 97, 107, 118, 124, 126, 127, 128, 132, 136, 141]
	Victorian Health Promotion, Youth Engagement Evaluation Framework	1/70 (1.4%)	[117]
**Not reported**	36/70 (51.4%)	[55, 57, 58, 59, 67, 68, 69, 70, 74, 75, 76, 77, 79, 81, 89, 90, 91, 94, 101, 102, 103, 104, 105, 108, 111, 115, 119, 120, 121, 122, 123, 125, 128, 131, 135, 143]

Abbreviations: CBPR: community‐based participatory research; YPAR: Youth Participatory Action Research.

^a^ Total count is greater than the included studies.

^b^ One study conducted a waste audit, nine studies completed a school or community food assessment, and two studies captured food marketing data.

^c^ Four studies included a Youth Alliance, two studies involved a youth advisory group or board, and one study included a student council.

^d^ One study included body maps and lifeline methodology, one study used poetry and graffiti art, one was a peer leadership program, one study led a fresh produce stall, and two studies were a community‐oriented project.

^e^ Twenty studies only included adolescents as traditional study participants in a consultative role and were not involved in any stage of the research cycle. Twenty studies involved adolescents as both traditional study participants and throughout the research cycle.

^f^ Two studies utilized two youth engagement frameworks.

Level of adolescent involvement as co‐researchers throughout the research cycle varied. Approximately half of studies (49%) involved adolescents in the formative stages (identifying the topic and/or the design and development) [60, 61, 62, 63, 65, 69, 81, 82, 83, 86, 87, 88, 93, 94, 95, 97, 99, 100, 104, 106, 107, 109, 111, 112, 114, 119, 120, 122, 124, 128, 129, 130, 133, 136, 137, 138, 139, 140, 141, 142]. However, 11 studies (16%) included adolescents throughout each stage [63, 65, 83, 86, 99, 109, 129, 130, 133, 136, 137, 138, 139, 140, 141, 142]. Conducting the research was the most common stage [55, 56, 58, 60, 61, 62, 63, 66, 69, 70, 71, 72, 73, 74, 77, 78, 80, 81, 82, 83, 85, 86, 87, 88, 90, 92, 93, 96, 97, 103, 106, 107, 111, 112, 113, 114, 116, 117, 118, 119, 123, 124, 128, 129, 130, 131, 132, 133, 136, 137, 138, 139, 140, 141, 142] through methods such as photovoice (27%) [55, 58, 65, 66, 77, 80, 83, 88, 92, 96, 103, 109, 118, 124, 129, 131, 132, 141, 142], Geographic Information System (GIS) mapping (9%) [56, 74, 78, 85, 123, 131], system mapping workshops (4%) [73, 113, 126], or collecting field data through other methods (9%) [55, 74, 77, 86, 96, 99, 106, 111, 119, 120, 127, 130, 142] such as corner store assessments. Twenty studies (29%) involved adolescents as both traditional study participants and throughout the research cycle as co‐researchers [55, 58, 60, 63, 77, 85, 92, 94, 96, 97, 100, 106, 107, 111, 113, 120, 123, 127, 128, 131]. A further 20 studies (29%) only included adolescents as a traditional study participants in a consultative mode through focus groups or surveys [57, 59, 64, 67, 68, 75, 76, 79, 84, 89, 91, 101, 102, 105, 108, 115, 121, 125, 135, 143].

#### 3.2.3 Engagement Frameworks

Approximately half of studies did not report using a youth engagement framework (51%) [55, 57, 58, 59, 67, 68, 69, 70, 74, 75, 76, 77, 79, 81, 89, 90, 91, 94, 101, 102, 103, 104, 105, 108, 111, 115, 119, 120, 121, 122, 123, 125, 128, 131, 135, 143]. Overall, 12 different frameworks were used. Three studies reported a power (4%) [65, 106, 138] or process‐focused framework (4%) [65, 78, 100], two studies utilized a framework that was impact‐focused (3%) [60, 93], and one was equity‐focused (1%) [142]. Twenty‐seven studies (39%) utilized a multi‐focused youth engagement framework such as Youth Participatory Action Research (YPAR) (19%) [56, 62, 66, 72, 73, 97, 107, 118, 124, 126, 127, 128, 132, 136, 141], Community Based Participatory Research (CBPR) (19%) [63, 64, 80, 83, 84, 85, 86, 92, 96, 99, 109, 113, 130, 133], or the Victorian Health Promotion Youth Engagement Evaluation Framework (1%) [117].

### 3.3 Impact of Participation

#### 3.3.1 Individual Level

Thirty‐one studies (44%) reported the impact of participation on adolescents. Common concepts that emerged include improved professional skills, well‐being, and motivated healthy behaviors. It was commonly reported that adolescents had gained confidence in public speaking, advocacy, and taking up leadership roles [61, 67, 69, 70, 76, 78, 85, 88, 92, 95, 106, 107, 111, 113, 118, 138]. Participation also provided valuable work experience enhancing their employability [67, 106, 126, 141]. Adolescents felt empowered that they could make a difference in their community and felt that their voices were being heard, which enhanced their sense of self‐worth, self‐esteem, and self‐efficacy [55, 69, 83, 88, 92, 95, 99, 111, 122, 123]. Some studies reported participants had increased knowledge and awareness of the upstream factors that influence people's diets. This motivated and inspired them to develop policy ideas [123, 126, 142] and healthier behaviors [69, 88, 94, 116, 122, 138]. Most of these studies were reported by adults who provided anecdotal or qualitative evidence, with only six studies (9%) reporting quantitative evidence of impact [88, 92, 107, 113, 114, 116].

#### 3.3.2 Community, National, and International Levels

Forty‐four studies (63%) reported policy‐related impact of adolescent participation. It was commonly reported that adolescents were provided opportunities to present their food environment projects and policy ideas to influential figures, including teachers, school principals, community organizations, and politicians [55, 61, 65, 74, 78, 81, 82, 92, 96, 103, 109, 117, 118, 126, 127]. There were also opportunities for adolescents to present at United Nations meetings [62]. Another concept that emerged was assisting the implementation of policy. Within a school setting, examples included improved menus with additional healthier items [57, 83, 88]. Water fountains and refill stations were also installed to reduce sugar‐sweetened beverage consumption [60, 71]. Community gardens and farmers' markets were also introduced within local communities to increase access to fresh produce [99, 106, 133, 141, 142]. There was also improved availability of healthy food in restaurants [63] and shops [130, 142]. Evidence of impact on a national and global scale was also reported, although less frequently. One study reported that their student council had contributed to the 2006 World Health Organization Regional Committee for the Western Pacific Region, assisting with New Zealand's launch of an obesity prevention initiative [61]. Another study had helped inform the Irish Obesity Policy and Action Plan 2016–2025 [90]. The CO‐CREATE study created a dialogue forum tool that was pilot tested at EAT Stockholm Food Forum and UNICEF's State of the World Children's Report launch [62].

### 3.4 Processes of Participation

#### 3.4.1 Barriers

Forty‐five studies (64%) reported or assessed barriers to participation. Common barriers reported from adolescent perspectives included not being taken seriously. They perceived their input could not make a difference, either due to not knowing how, having past experiences of their ideas not being actioned, or being afraid of sharing their ideas [66, 97, 108, 128, 138]. This was a common experience also reported by researchers [121, 130, 135] and expressed the need to address power dynamics to motivate adolescents to voice their opinions [106, 131]. Other concepts that emerged from researchers' perspectives were limited resources and time needed to build genuine connections with adolescents and community members, as well as ensuring adequate training for adolescents participating [61, 63, 65, 67, 74, 77, 88, 90, 92, 126, 138]. Seeking ethical approval also caused challenges with recruitment [73, 75, 80]. One study also commented on the need to consider whether ethics are required if adolescents are not traditional study participants who are instead research collaborators [126].

#### 3.4.2 Enablers

Enabling factors for adolescent participation were reported by 57 studies (81%). Having a trusted mentor or adult facilitator was considered important to create a safe and supportive environment to empower adolescents to participate and voice their views [62, 69, 72, 74, 85, 138]. Adolescents also emphasized the importance of feeling valued, where they felt their ideas were being heard and actioned. Other factors that influenced participation from an adolescent perspective included being interested or passionate about the topic [55, 97] and using participatory methods that were fun and engaging [80, 92, 99, 111, 118, 123]. Researchers had reported that participatory methods had also facilitated removing power imbalances [73, 77, 78, 98, 103, 108, 117, 123, 127, 128, 142]. Further, most studies reported they delivered training sessions for adolescents about how to conduct ethical research [55, 58, 63, 65, 66, 74, 81, 83, 90, 92, 94, 97, 99, 106, 109, 123, 124, 129, 130, 133, 137, 142]. They also provided reimbursements [55, 56, 67, 74, 79, 83, 89, 96, 97, 100, 111, 142] and resources needed to participate, such as cameras to conduct photovoice [58, 66, 83, 85, 92, 94, 99, 103, 109, 118, 132, 133].

## 4 Discussion

Our scoping review highlights there is substantial evidence of adolescents participating at various modes, methods, and levels of the research cycle to inform food environment policy research, with a greater focus on food provision and retail. However, studies examining the extent of participation were predominantly conducted in high‐income countries and limited by inconsistent reporting of socio‐demographic characteristics. Our review also highlighted the impact of adolescent participation across multiple levels, including studies reporting the implementation of food environment policies. Evidence of impact on an individual level was limited by a lack of robust, empirical evidence. Additionally, we identified common barriers and enablers that are important considerations to ensure supportive environments are fostered for adolescents to meaningfully engage in research so they can be key agents of food environment transformation.

While most studies included adolescent‐led projects (41%), the level of adolescent participation varied. Approximately half of the included studies (49%) reported involving adolescents in the first two formative stages of research, allowing them to have more influence over the research process and outcomes [24]. Only 16% of studies included adolescents across each research stage. Process factors revealed involving adolescents as co‐researchers often requires capacity building, which can be limited by a lack of resources and time, as similarly reported elsewhere [144, 145]. Acknowledging these challenges, it has been recommended for researchers to support adolescents to become co‐researchers to share their unique skills and meaningfully contribute to the decision‐making process [146]. However, a rapid review identified that adolescents felt they could make a meaningful contribution to the research even if they were not involved in all stages [147]. They similarly reported conducting research was the most popular research stage, using methods such as photovoice that suited young people's capabilities [147]. In contrast, other reviews have reported greater adolescent participation in NCD prevention research through consultative modes [26] and throughout the design or dissemination phase in health research [26, 148]. This may be reflective of the growing literature in recent years promoting participatory methods, where our review identified an upward trend with most studies (17%) published in 2023. However, it is important to recognize that some modes of participation may be more appropriate or feasible depending on the policy context.

Our review identified limited representation from LMICs, highlighting disproportionate opportunities available for adolescents to participate in research to inform food environment policies. This may be due to a lack of resources, funding, or competing public health priorities [26, 149, 150]. We identified only one study (1%) that included LMICs [117]. In this study, Fleming et al. consulted 640 adolescents across 18 countries (11 from LMICs) about how to improve sustainable food systems [117]. A key concern from adolescents in LMICs was the affordability of healthy food. Further, adolescents collectively were calling for greater inclusion in food system policy at both a local and global scale. LMICs represent a greater proportion of the world's adolescents that are facing the triple burden of malnutrition [149]. Yet, most evidence on adolescent health and nutrition is generated within high‐income countries, leading to challenges in informing evidence‐based, context‐specific policies that meet adolescents' needs [151]. Therefore, greater investment is needed to improve equitable opportunities for adolescents to be central actors in food environment transformation and achieve the SDGs [10, 146].

Inconsistent reporting of socio‐demographic characteristics, including socio‐economic status (51%), gender (33%) and age (23%), further emphasizes the need to ensure opportunities are accessible for adolescents, particularly from marginalized or underserved communities [146, 147, 152]. Adequate demographic reporting in health research, such as through the PROGRESS‐Plus framework [153], is important when informing food environment policy to support population health, especially minority population groups who often experience greater health inequities [154, 155]. Utilizing multi‐focused youth engagement frameworks, such as YPAR, may enhance reporting of the level of inclusiveness [53, 147]. Self‐selection bias is a common limitation across youth engagement initiatives, further highlighting the need for transparent reporting [25]. Our review additionally identified limited male and gender‐diverse participation. One study (1%) reported challenges recruiting adolescent males despite targeted approaches [126]. Our review highlighted that interest in the topic was a key enabler in participating, which may have influenced low participation rates from males. This may also reflect traditional gender roles, where females historically are responsible for food production, procurement, preparation, and family nutrition across cultures [156, 157]. UNICEF is advocating for a gender‐transformative approach to address underlying determinants of malnutrition, including empowering adolescent girls and women to have increased agency to participate in nutrition decisions impacting them [158]. This reinforces the importance of reporting socio‐demographic characteristics to appropriately monitor and evaluate progress towards attaining equitable and optimal nutrition for all.

Our review revealed that most studies did not adequately plan for robust evaluation to assess the impact of adolescent participation on an individual level. It is well documented across the literature that involving adolescents in research as co‐researchers can have a positive impact on them [33, 144, 159, 160]. This includes self‐development, increased empowerment, self‐esteem, and confidence that they can make a difference, as similarly identified in our review. However, a recent umbrella review highlighted that despite 99 reviews of adolescent involvement in health research, there is limited robust empirical evidence evaluating its positive impact [33]. Our review identified similar findings, with most evidence of impact limited to anecdotal evidence. Robust evaluations are required to ensure meaningful adolescent participation and assess high‐quality policy relevant research that meets adolescents needs [32]. Nevertheless, we identified examples where adolescent participation was associated with community, national, and international level impact. For example, Youth Alliance members from the CO‐CREATE project advocated to EU policymakers to restrict marketing of unhealthy food and drinks for people aged under 18 years [62]. Additionally, we identified studies that reported adolescent participation had helped shape the implementation of food environment policies, such as through healthier food menus, installed water fountains, and community gardens within schools [57, 61, 71, 83, 88, 99, 106, 133, 141, 142].

Our review identified several barriers to adolescent participation in research. Ethical considerations were raised across studies, including lengthy ethics procedures causing delays in recruitment within a constrained project timeline [73, 75, 80]. This barrier is commonly raised across the literature, including a debate on the age at which adolescents no longer require parental consent to participate [126, 145, 146, 147]. Further, it brings into question whether ethical approval is required if adolescents are participating as co‐researchers instead of a traditional study participants, which in turn may limit the reporting of socio‐demographic characteristics. Our review identified that 57% of studies involved adolescents as traditional study participants, with half being consulted about a food‐EPI policy domain, commonly through focus groups or surveys. The other half were also participating as co‐researchers. This highlights the complexity and nuance of ensuring ethical standards are met, especially if the impact of adolescent participation is being evaluated, or their perceptions and ideas on food environment policy are being analyzed [48]. These ethical challenges can often deter researchers from including adolescents to participate as co‐researchers [148]. Further, treating adolescent co‐researchers as subjects of the research may challenge reducing power imbalances and limit adolescents feeling like valued members of the research team [145, 147]. Researcher reflexivity is required to ensure ethical and rigorous research is conducted whilst maintaining adolescent autonomy [147, 161, 162]. This is reflected in the recent guidelines co‐created with young people by Guo and colleagues that provide over 20 recommendations to safeguard youth mental health and wellbeing in youth engagement initiatives [163].

Understanding the process factors that enable meaningful adolescent participation in research is necessary to provide appropriate support and infrastructure required for adolescents to be agents of food environment transformation [156]. Our review revealed the importance of empowering adolescents to voice their perspectives on food‐environment‐related policy as well as having transparent communication about actioning their ideas. Additionally, each mode of adolescent participation can have a purpose in informing food environment policy that meets adolescent needs. However, this can be context‐ and policy‐level specific. It is important to consider the amount of resources available to ensure adolescents can meaningfully engage. Adolescent co‐researcher perspectives reveal the importance of ensuring opportunities to participate are accessible for priority population groups to be included in food environment policies to inform evidence‐based, context‐specific policies that meet adolescents needs (Box 1). Ensuring transparency and a rights‐based approach aligns with United Nations' guiding core principles for meaningful youth engagement in policymaking and decision‐making processes [164].

Box 1 **Recommendations for future research from adolescent co‐researcher perspectives.**

1. Provide opportunities for adolescents from priority population groups to be included and represented in research to inform food environment policies that meet their needs.
2. Through adolescent and community partnerships, conduct long‐term studies that align with the full picture of adolescents' realities to assess the impact and implementation of food policies
3. Clearly define the purpose of adolescent involvement and how they are participating in research, whether as co‐researchers or traditional study participants, to understand power dynamics and plan for robust evaluation to assess impact appropriately.
4. Ensure researchers and research teams have appropriate training to ensure that adolescents in research decision‐making roles, like co‐researchers, have shared power.
5. Employ participatory methodologies that are easily applicable and acceptable for adolescents to transfer into local communities. Use engaging participatory methods such as photovoice and everyday digital platforms to actively engage adolescents from diverse backgrounds in research.
6. Ensure supportive environments are created through mentorship and skill building activities (for, e.g., health literacy, critical thinking, and research methods) to inspire and motivate adolescents to meaningfully contribute to research, inspire collective action, and improve food environments. This may also build the confidence of researchers to involve adolescent co‐researchers.
7. Provide appropriate recognition and leadership opportunities for adolescents participating in research, such as financial reimbursement, co‐authorship, and recommendation letters to support adolescents educational and career aspirations.

To our knowledge, this is the first review to identify how adolescents are meaningfully participating in research focused on improving food environments. A key strength of this review is that adolescents informed each stage, increasing research relevance and dissemination of results [42]. A comprehensive search was conducted, including studies of any language or time of publication and adhered to the PRISMA‐SCr guidelines [46]. However, our search strategy was limited to empirical research due to the large volume of peer‐reviewed studies identified. Therefore, this review does not capture the extent of adolescent participation across gray literature focused on food environment policies, highlighting areas for future research.

Despite a thorough search strategy, it is possible that some studies were missed, likely due to the term “participation” being commonly used to refer to individuals participating in research. Further, the assessment of meaningful adolescent participation can be subjective and not always explicitly reported among studies. This also limited our ability to assess the impact of adolescent participation in relation to the study's intended aims of their involvement. To overcome this, all studies were individually dual assessed, and data extracted were cross checked for accuracy among 20% of included studies. Lastly, the long‐term impact of adolescent participation on policy development and implementation may not be known within short project timelines and restricted research funding, therefore making it difficult to assess or report [165]. Future research should aim to assess whether adolescent participation leads to lasting policy change to improve healthy food environments.

## 5 Conclusion

There is substantial evidence of adolescents participating in various modes, methods, and levels of research to transform healthy food environments through policy, demonstrating that adolescents can be central actors for change. However, evidence was limited by inconsistent reporting and a lack of empirical evidence of impact. Ensuring diverse representation of adolescents from LMICs and culturally and ethnically diverse groups will be necessary to achieve the SDGs and improve the global NCD burden.

## Author Contributions

A.R.T., P.F., A.A.G., S.R.P., and HAPYUS conceptualized the study. A.R.T., P.N.A.D., S.J., P.F., A.A.G., and S.R.P. conducted screening. A.R.T. led data extraction, formal analysis, visualization, and drafted the initial manuscript. HAPYUS led recommendations for future research. P.F., A.A.G., and S.R.P. provided supervision and verified the underlying data reported in the manuscript. All authors contributed to the study protocol as well as revised, edited, and approved the manuscript.

## Conflicts of Interest

The authors declare no conflicts of interest.

## Supporting information


**Table S1** Preferred Reporting Items for Systematic reviews and Meta‐Analyses extension for Scoping Reviews (PRISMA‐ScR) checklist.
**Figure S1.** Medline search screenshot.
**Table S2.** Reasons for the exclusion of articles from full‐text screening (*n* = 222).
**Table S3.** Data extraction of included studies—study characteristics (*n* = 70).
**Table S4.** Data extraction of included studies—extent (*n* = 70).
**Table S5.** Data extraction of included studies—impact and process (*n* = 70).
