# Supplementary material for: How Are Adolescents Participating in the Transformation of Healthy Food Environments? A Scoping Review of Empirical Research
Source: Obes Rev. 2025 Jul 24;27(1):e70002. doi: 10.1111/obr.70002 (PMC12685493; doi:10.1111/obr.70002)
Supplement: Supplementary file 1 — Table S1 Preferred Reporting Items for Systematic reviews and Meta‐Analyses extension for Scoping Reviews (PRISMA‐ScR) checklist. Figure S1. Medline search screenshot. Table S2. Reasons for the exclusion of articles from full‐text screening (n = 222). Table S3. Data extraction of included studies—study characteristics (n = 70). Table S4. Data extraction of included studies—extent (n = 70). Table S5. Data extraction of included studies—impact and process (n = 70). [file OBR-27-e70002-s001.pdf]

Supplementary material for: How are adolescents participating in the transformation of healthy food environments? A scoping review of empirical research

Authors: Allyson R. Todd*[1,2], Putu Novi Arfirsta Dharmayani[3], Sisi Jia[1,2], Rebecca Raeside[1,2], Seema Mihrshahi[3], Katrina E. Champion[4,5], HAPYUS[1], Penny Farrell[2,4], Alice A. Gibson[2,4,6], Stephanie R. Partridge[1,2]

Affiliations:

1. Susan Wakil School of Nursing and Midwifery, Faculty of Medicine and Health, The University of Sydney
2. Charles Perkins Centre, The University of Sydney
3. Department of Health Sciences, Faculty of Medicine, Health, and Human Sciences, Macquarie University
4. School of Public Health, Faculty of Medicine and Health, The University of Sydney
5. The Matilda Centre, Faculty of Medicine and Health, The University of Sydney
6. The Leeder Centre for Health Policy, Economics & Data, Faculty of Medicine and Health, The University of Sydney

HAPYUS authors: Moudasir Jalili, Natalie Ryan, Elena Wang

Corresponding author*:
Allyson R. Todd
Email: allyson.todd@sydney.edu.au
Address: Level 8, Susan Wakil Health Building, The University of Sydney, Camperdown, NSW, 2006 Australia

Table S1. Preferred Reporting Items for Systematic reviews and Meta-Analyses extension for Scoping Reviews (PRISMA-ScR) Checklist

SECTION	ITEM	PRISMA-ScR CHECKLIST ITEM	REPORTED ON PAGE #
**TITLE**			
Title	1	Identify the report as a scoping review.	1
**ABSTRACT**			
Structured summary	2	Provide a structured summary that includes (as applicable): background, objectives, eligibility criteria, sources of evidence, charting methods, results, and conclusions that relate to the review questions and objectives.	3
**INTRODUCTION**			
Rationale	3	Describe the rationale for the review in the context of what is already known. Explain why the review questions/objectives lend themselves to a scoping review approach.	4-6
Objectives	4	Provide an explicit statement of the questions and objectives being addressed with reference to their key elements (e.g., population or participants, concepts, and context) or other relevant key elements used to conceptualize the review questions and/or objectives.	6
**METHODS**			
Protocol and registration	5	Indicate whether a review protocol exists; state if and where it can be accessed (e.g., a Web address); and if available, provide registration information, including the registration number.	7
Eligibility criteria	6	Specify characteristics of the sources of evidence used as eligibility criteria (e.g., years considered, language, and publication status), and provide a rationale.	7-8
Information sources*	7	Describe all information sources in the search (e.g., databases with dates of coverage and contact with authors to identify additional sources), as well as the date the most recent search was executed.	8
Search	8	Present the full electronic search strategy for at least 1 database, including any limits used, such that it could be repeated.	8
Selection of sources of evidence†	9	State the process for selecting sources of evidence (i.e., screening and eligibility) included in the scoping review.	8-9
Data charting process‡	10	Describe the methods of charting data from the included sources of evidence (e.g., calibrated forms or forms that have been tested by the team before their use, and whether data charting was done independently or in duplicate) and any processes for obtaining and confirming data from investigators.	9
Data items	11	List and define all variables for which data were sought and any assumptions and simplifications made.	9
Critical appraisal of individual sources of evidence§	12	If done, provide a rationale for conducting a critical appraisal of included sources of evidence; describe the methods used and how this information was used in any data synthesis (if appropriate).	N/A

SECTION	ITEM	PRISMA-ScR CHECKLIST ITEM	REPORTED ON PAGE #
Synthesis of results	13	Describe the methods of handling and summarizing the data that were charted.	9
**RESULTS**			
Selection of sources of evidence	14	Give numbers of sources of evidence screened, assessed for eligibility, and included in the review, with reasons for exclusions at each stage, ideally using a flow diagram.	10
Characteristics of sources of evidence	15	For each source of evidence, present characteristics for which data were charted and provide the citations.	Table 2, 11
Critical appraisal within sources of evidence	16	If done, present data on critical appraisal of included sources of evidence (see item 12).	N/A
Results of individual sources of evidence	17	For each included source of evidence, present the relevant data that were charted that relate to the review questions and objectives.	10-15
Synthesis of results	18	Summarize and/or present the charting results as they relate to the review questions and objectives.	10-15
**DISCUSSION**			
Summary of evidence	19	Summarize the main results (including an overview of concepts, themes, and types of evidence available), link to the review questions and objectives, and consider the relevance to key groups.	16-19
Limitations	20	Discuss the limitations of the scoping review process.	20
Conclusions	21	Provide a general interpretation of the results with respect to the review questions and objectives, as well as potential implications and/or next steps.	20
**FUNDING**			
Funding	22	Describe sources of funding for the included sources of evidence, as well as sources of funding for the scoping review. Describe the role of the funders of the scoping review.	2

JBI = Joanna Briggs Institute; PRISMA-ScR = Preferred Reporting Items for Systematic reviews and Meta-Analyses extension for Scoping Reviews.
* Where *sources of evidence* (see second footnote) are compiled from, such as bibliographic databases, social media platforms, and Web sites.
† A more inclusive/heterogeneous term used to account for the different types of evidence or data sources (e.g., quantitative and/or qualitative research, expert opinion, and policy documents) that may be eligible in a scoping review as opposed to only studies. This is not to be confused with *information sources* (see first footnote).
‡ The frameworks by Arksey and O'Malley (6) and Levac and colleagues (7) and the JBI guidance (4, 5) refer to the process of data extraction in a scoping review as data charting.
§ The process of systematically examining research evidence to assess its validity, results, and relevance before using it to inform a decision. This term is used for items 12 and 19 instead of "risk of bias" (which is more applicable to systematic reviews of interventions) to include and acknowledge the various sources of evidence that may be used in a scoping review (e.g., quantitative and/or qualitative research, expert opinion, and policy document).

From: Tricco AC, Lillie E, Zarin W, O'Brien KK, Colquhoun H, Levac D, et al. PRISMA Extension for Scoping Reviews (PRISMAScR): Checklist and Explanation. Ann Intern Med. 2018;169:467–473. doi: 10.7326/M18-0850.

Fig S1. Medline Search Screenshot

Search History (33)

View Saved

# ▲	Searches	Results	Type	Actions		Annotations
1	exp Policy Making/	28667	Advanced	Display Results	More	
2	exp Guideline/	38383	Advanced	Display Results	More	
3	Government Programs/	6563	Advanced	Display Results	More	
4	Health Policy/	73081	Advanced	Display Results	More	
5	Government Regulation/	21920	Advanced	Display Results	More	
6	decision making/ or decision making, shared/	107446	Advanced	Display Results	More	
7	Leadership/	48731	Advanced	Display Results	More	
8	political activism/ or stakeholder participation/	2490	Advanced	Display Results	More	
9	exp Community-Based Participatory Research/	6328	Advanced	Display Results	More	
10	Community Participation/	18635	Advanced	Display Results	More	
11	Child Advocacy/	4219	Advanced	Display Results	More	
12	civil rights/ or consumer advocacy/ or right to health/	13937	Advanced	Display Results	More	
13	(decision mak* or policy mak* or policy-mak* or advisory committee* or advisory board* or advisory group* or advisory structure* or steering committee* or steering group* or agenc* or youth engagement* or stakeholder participation* or stakeholder engagement* or adolescent participation or adolescent engagement or politic* or activist* or citizen scienc* or community particpat* or community research* or guideline* or co-design* or codesign* or co-production* or co-creat* or cocreat* or regulat* or strateg*).mp.	5348303	Advanced	Display Results	More	
14	1 or 2 or 3 or 4 or 5 or 6 or 7 or 8 or 9 or 10 or 11 or 12 or 13	5453512	Advanced	Display Results	More	
15	Nutrition Policy/	10826	Advanced	Display Results	More	
16	Nutritive Value/	16197	Advanced	Display Results	More	
17	Food Industry/	6324	Advanced	Display Results	More	
18	Diet, Healthy/	7154	Advanced	Display Results	More	
19	Fast Foods/	3273	Advanced	Display Results	More	
20	Nutritional Requirements/	20137	Advanced	Display Results	More	
21	sugars/ or dietary sugars/	6879	Advanced	Display Results	More	
22	Food Packaging/	6958	Advanced	Display Results	More	
23	Food Labeling/	4657	Advanced	Display Results	More	
24	Taxes/	7759	Advanced	Display Results	More	
25	malnutrition/ or overnutrition/ or overweight/ or obesity/	259077	Advanced	Display Results	More	
26	Eating/	60624	Advanced	Display Results	More	
27	(food environment* or digital food environment* or nutrition polic* or fastfood* or food industr* or junk food* or public health nutrit* or sustainab* or environmental justice or food system* or food systems transform* or sustain* food system* or food security or food insecurity or food label* or energy or nutrition* content or energy intake* or food outlet access* or GIS or geograph* or availab* or area* or spatial distribut* or nutri* composition or qual* or food composition or portion siz* or food marketing* or food promot* or advertis* or food pric* or tax* or afforab* or cost* or food purchas* or food provision or food retail* or food marketing* or food promot* or advertis* or food trad* or food investment*).mp.	6955811	Advanced	Display Results	More	
28	15 or 16 or 17 or 18 or 19 or 20 or 21 or 22 or 23 or 24 or 25 or 26 or 27	7196269	Advanced	Display Results	More	
29	(Young adult participat* or Adolescent Participat* or young people participat* or Youth participat* or Teen* participat* or youth participatory action research).mp.	1045	Advanced	Display Results	More	
30	(("young adult*" or "young people*" or adolesc* or youth* or teen*) adj4 (engagement or participat* or "decision?mak*" or "policy?mak*" or leadership* or advocacy or "advisory committee*" or "advisory board*" or "advisory group*" or "advisory structure*" or "steering committee*" or "steering group*" or "user?generat*" or "activis*" or "co?design*" or "codevelop*" or "co?plan*" or "co?evaluat*" or "co?deliver*" or "co?produc*")).tw.	11366	Advanced	Display Results	More	
31	(("young adult*" or young people* or youth* or teen*) adj3 (Engag* or partcipat* or coproduc* or participatory action* or civic engage*)).mp.	3202	Advanced	Display Results	More	
32	29 or 30 or 31	13290	Advanced	Display Results	More	
33	14 and 28 and 32	1993	Advanced	Display Results	More	

Save Remove Combine with: AND OR

Contract

Table S2. Reasons for the exclusion of articles from full-text screening (n=222).

Article title	Reasons for exclusion
'Nothing Gets Realised Anyway': Adolescents' Experience of Co-Creating Health Promotion Measures in Municipalities in Norway	Wrong intervention
'You are nearly an adult now': Engaging adolescents in nutrition programming is critical	Wrong study design or publication type
"I feel like a kid again": the voices of youth experiencing homelessness in a mobile recreation program	Wrong intervention
"We make a direct impact on people's lives": Youth empowerment in the context of a youth-led participatory budgeting project	Wrong intervention
(Re)thinking (re)connection: Young people, “natures” and the water–energy–food nexus in São Paulo State, Brazil	Wrong intervention
[English Translation: Adolescents' and young people's experiences of health participation during the COVID-19 pandemic in Chile] Experiencias de participacao de adolescentes e jovens na saude em tempos de pandemia de COVID-19 no Chile	Wrong intervention
"I see myself really as a public health activist”: A critical analysis of young people's voices in the National Health Insurance policy submissions	Wrong intervention
Youth Leadership in Action (YLIA): Feasibility of Implementing a Youth Participatory Action Research Program in Inner-City Public Schools and Communities	Wrong study design or publication type
"More Worthwhile than the Paycheck": An Innovative Summer Internship in Northern Manhattan to Engage Youth in Food Justice Promotion	Wrong study design or publication type
Co-Creation of Context-Specific Digital Resources for Empowering Adolescents Health	Wrong study design or publication type
A City for All Citizens: Integrating Children and Youth from Marginalized Populations into City Planning	Wrong intervention
A coordinated school health program approach to adolescent obesity	Wrong outcome
A new global policy framework for adolescent nutrition?	Wrong study design or publication type
A picture's worth a thousand words: engaging youth in CBPR using the creative arts	Wrong intervention
A process evaluation of student participation in a whole school food programme	Wrong study population
A Qualitative Transdisciplinary Exploration of Children and Young People's Responses to High in Fat, Salt or Sugar Food and Beverage Brand Advertising	Wrong study design or publication type

A youth empowerment intervention to prevent childhood obesity: design and methods for a cluster randomized trial of the H2GO! program	Wrong intervention
A Youth Health Leadership Program: Feasibility and Initial Outcomes	Wrong intervention
A youth mentor-led nutritional intervention in urban recreation centers: a promising strategy for childhood obesity prevention in low-income neighborhoods	Wrong study population
Activating Student Voice through Youth Participatory Action Research (YPAR): Policy-Making That Strengthens Urban Education Reform	Wrong intervention
Adolescent nutritional awareness and use of food labels: results from the National Nutrition Health and Examination Survey	Wrong outcome
Adolescent Rights and the "First 1,000 days" Global Nutrition Movement: A View from Guatemala	Wrong study design or publication type
Adolescent women as a key target population for community nutrition education programs in Indonesia	Wrong outcome
Adolescents as multipliers of school health program: Prevention among peers	Wrong intervention
Adolescents encouraging healthy lifestyles through a peer-led social marketing intervention: Training and key competencies learned by peer leaders	Wrong intervention
Adolescents opinions regarding Plato del Bien Comer Maya as nutrition health promotion tool	Wrong intervention
Adolescents' engagement with unhealthy food and beverage brands on social media	Wrong outcome
Adolescents' influence in family meal decisions	Wrong intervention
Adults and children prefer a plate food guide relative to a pyramid	Wrong intervention
Alternative Food Networks in Food System Transition-Values, Motivation, and Capacity Building among Young Swedish Market Gardeners	Wrong outcome
AMP Centers: A Case Study of the Intersection of Critical Urban Education and Action Research	Wrong intervention
An evaluation of the NHS England Youth Forum	Wrong intervention
Are young adults,Äô discussions of public health nutrition policies associated with common food industry discourses? A qualitative pilot study	Wrong study population
Assessment on public perception towards the development of 1Malaysia youth cities	Wrong outcome
Association between junk food consumption and fast-food outlet access near school among Quebec secondary-school children: findings from the Quebec Health Survey of High School Students (QHSHSS) 2010-11	Wrong outcome

Association of the Healthy, Hunger-Free Kids Act With Dietary Quality Among Children in the US National School Lunch Program	Wrong outcome
Associations between the school food environment, student consumption and body mass index of Canadian adolescents	Wrong outcome
Associations of Food Stamp Participation With Dietary Quality and Obesity in Children	Wrong outcome
Balancing Community and University Aims in Community-Based Participatory Research: A Pacific Islander Youth Study	Wrong outcome
Between Rhetoric and Reality: Learnings From Youth Participation in the Adolescent and Youth Health Policy in South Africa	Wrong intervention
Beyond Access and Supply: Youth-Led Strategies to Captivate Young People's Interest in and Demand for Youth Programs and Opportunities	Wrong study design or publication type
Building school-based cardiovascular health promotion capacity in youth: a mixed methods study	Wrong intervention
Cameras in the Hands of Indigenous Youth: Participation, Films, and Nutrition in India	Wrong outcome
Can Asset-Based Community Development with Children and Youth Enhance the Level of Participation in Health Promotion Projects? A Qualitative Meta-Synthesis	Wrong study design or publication type
Can the current youth agricultural groups be sustainable? Experiences from Mid-Western Uganda	Wrong intervention
Caught in a 'spiral'. Barriers to healthy eating and dietary health promotion needs from the perspective of unemployed young people and their service providers	Wrong study population
Change in dietary energy density after implementation of the Teas Public School Nutrition Policy	Wrong outcome
Changes in children's and adolescents' dietary intake after the implementation of Chile's law of food labeling, advertising and sales in schools: a longitudinal study	Wrong outcome
Changes in local school policies and practices in Washington State after an unfunded physical activity and nutrition mandate	Wrong outcome
Changing the food environment: the French experience	Wrong outcome
Chic@s en Accion: Creciendo Sanos	Wrong study design or publication type
Child and adolescent fast-food choice and the influence of calorie labeling: a natural experiment	Wrong outcome
Children and Youth Participation in Decision-Making in Tanzania: A Need for Vibrant Councils at Local Levels	Wrong intervention
Children in All Policies (CAP) 2030 Citizen Science for Climate Change Resilience: a cross-sectional pilot study engaging adolescents to study climate hazards, biodiversity and nutrition in rural Nepal	Wrong intervention

Co-creating public health measures with adolescents in municipalities: municipal actors' views on inhibitors and promoters for adolescent involvement	Wrong intervention
Co-designing Pathways to Opportunities for Young People in the North West of England	Wrong intervention
Co-production of youth advocacy videos on food marketing and promotions	Wrong study population
Community perspectives on the use of regulation and law for obesity prevention in children: A citizens' jury	Wrong outcome
Community-based approaches to reducing health inequities and fostering environmental justice through global youth-engaged citizen science	Wrong study design or publication type
Creating community action plans for obesity prevention using the ANGELO (Analysis Grid for Elements Linked to Obesity) Framework	Wrong intervention
Cultural Connectedness as Obesity Prevention: Indigenous Youth Perspectives on Feast for the Future	Wrong study population
Development and implementation of Australian State, territory, and national policy on the health and wellbeing of adolescents and young adults: An exploration of policy actor perspectives using the Consolidated Framework for Implementation Research	Wrong outcome
Development of Young Adults Eating and Active for Health (YEAH) internet-based intervention via a community-based participatory research model	Wrong study population
Dietary guidelines in Singapore	Wrong outcome
Digital Humanitarians for the Sustainable Development Goals: YouthMappers as a Hybrid Movement	Wrong intervention
Digital media, youth practices and representations of recent activism in Portugal	Wrong intervention
Digital tools for youth health promotion: principles, policies and practices in sub-Saharan Africa	Wrong intervention
Dutch Adolescents' Narratives of Their Citizenship Efficacy "Hypothetically, I Could Have an Impact"	Wrong intervention
Educators and Youth Activists: A Negotiation over Enhancing Students' Role in School Life	Wrong intervention
Effect of the Supplemental Nutrition Assistance Program (SNAP) on Frequency of Beverage Consumption among Youth in the United States	Wrong outcome
eHealth promotion and social innovation with youth: using social and visual media to engage diverse communities	Wrong study design or publication type
Enabling improvements in adolescent nutrition through youth inclusive and gender responsive programs at scale	Wrong study design or publication type
Encouraging greater empowerment for adolescents in consent procedures in social science research and policy projects	Wrong intervention

Engagement of Adolescents in a Health Communications Program to Prevent Noncommunicable Diseases: Multiplicadores Jóvenes, Lima, Peru, 2011	Wrong intervention
Engaging and sustaining adolescents in community-based participatory research: structuring a youth-friendly community-based participatory research environment	Wrong intervention
Engaging communities to develop and sustain comprehensive wellness policies: Louisiana's schools putting prevention to work	Wrong outcome
Engaging Young People as Partners for Change: The UR Community Project	Wrong intervention
Engaging youth as citizen scientists to determine health needs of New Brunswick adults	Wrong study population
Enhancing youth participation in local governance: an assessment of urban and rural junior councils in Zimbabwe	Wrong intervention
Erratum to "Perspectives of the key stakeholders of the KickStart for Kids school breakfast program".	Wrong outcome
Evaluating Project Safe Neighborhoods in Connecticut: a Youth Opportunity Initiative	Wrong intervention
Examining changes in school vending machine beverage availability and sugar-sweetened beverage intake among Canadian adolescents participating in the COMPASS study: a longitudinal assessment of provincial school nutrition policy compliance and effectiveness	Wrong outcome
Experiences of overweight/obese adolescents in navigating their home food environment	Wrong intervention
Exploration of Youth Engagement Practices in Environmental Activities Construct	Wrong intervention
Exploring influences on adolescent diet and physical activity in rural Gambia, West Africa: food insecurity, culture and the natural environment	Wrong outcome
Exploring Models for Youth Engagement in Community Health Planning: The Youth-led Community Health Learning Initiative	Wrong study population
Exploring the opportunities for food and drink purchasing and consumption by teenagers during their journeys between home and school: a feasibility study using a novel method	Wrong outcome
Exploring the perceptions of and experiences with traditional foods among first nations female youth: A participatory photovoice study	Wrong intervention
Exploring the School Nutrition Policy Environment in Canada Using the ANGELO Framework	Wrong outcome
Familias Unidas for Health and Wellness: Adapting an Evidence-Based Substance Use and Sexual Risk Behavior Intervention for Obesity Prevention in Hispanic Adolescents	Wrong intervention
Family influences on health and nutrition practices of pregnant adolescents in Bangladesh	Wrong intervention

Fijian youth entrepreneurs: championing health through sustainable food systems	Wrong study population
Food advertising towards children and young people in Norway	Wrong outcome
Food and Beverage Marketing to Youth	Wrong study design or publication type
Food poverty and youth work ,A community response	Wrong intervention
Food shopping and label use behavior among high school-aged adolescents	Wrong outcome
Food- based dietary guidelines for Filipinos: retrospects and prospects	Wrong outcome
Formative Evaluation to Increase Availability of Healthy Snacks and Beverages in Stores Near Schools in Two Rural Oregon Counties, 2013	Wrong outcome
Fostering social relationships through food rituals in a New Zealand school	Wrong intervention
Fostering Youth-Enabling Environments: A Participatory Affordance-Capability Framework for the Development and Use of Youth-Engaged Environmental Assessments	Wrong intervention
From youth engagement to policy insights: Identifying and testing food systems' sustainability indicators	Wrong study population
Giving young people a voice in their health services: A local initiative	Wrong study design or publication type
Global Kids Organizing in the Global City: Generation of Social Capital in a Youth Organizing Program	Wrong study design or publication type
Guiding, sustaining and growing the public involvement of young people in an adolescent health research community of practice	Wrong intervention
How Do Adolescents View Health? Implications for State Health Policy	Wrong study population
How High Schools Become Empowering Communities: A Mixed-Method Explanatory Inquiry into Youth-Adult Partnership and School Engagement	Wrong intervention
Impact of Engaging Students in Health Survey Data Analysis and as Full Partners in School Change: Results From a Mixed Methods Study	Wrong intervention
Impact of front-of-pack nutrition information and label design on children's choice of two snack foods: Comparison of warnings and the traffic-light system	Wrong outcome
Improving youth engagement by local governments: A Swedish case study	Wrong intervention
In Their Own Words: Engaging Young People in a Youth Research Advisory Group	Wrong intervention
Incorporating youth-led community participatory research into school health center programs and policies	Wrong intervention

Increased School Breakfast Participation from Policy and Program Innovation: The Community Eligibility Provision and Breakfast after the Bell	Wrong outcome
Integrating youth in city planning: Developing a participatory tool toward a child-friendly vision of Eastern Wastani – Saida	Wrong intervention
Integrating Youth Voice in Health Plan Quality Improvement	Wrong intervention
Intentions of Young Farmers Club (YFC) Members to Pursue Career Preparation in Agriculture: The Case of Uganda	Wrong intervention
Involving young people as researchers: uncovering multiple power relations among youths	Wrong intervention
Knowledge, opportunities and barriers to participate in youth support politics	Wrong intervention
Lessons from a peer-led obesity prevention programme in English schools	Wrong intervention
Limiting the consumption of sugar sweetened beverages in Mexico's obesogenic environment: A qualitative policy review and stakeholder analysis	Wrong outcome
Linking health and the environment through education, A Traditional Food Program in Inuvik, Western Canadian Arctic	Wrong intervention
Living Tensions of Co-Creating a Wellness Program and Narrative Inquiry alongside Urban Aboriginal Youth	Wrong intervention
Making healthy food choices using nutrition facts panels. The roles of knowledge, motivation, dietary modifications goals, and age	Wrong outcome
Mobilizing European youth to fight childhood obesity. How data from WHO/Europe COSI study and initiatives from CO-CREATE project are helping to drive policy progress	Wrong study design or publication type
More than a Nuisance: Implications of Food Marketing for Public Health Efforts to Curb Childhood Obesity	Wrong outcome
Multidisciplinary approaches to address food insecurity and nutrition among youth and their families	Wrong study design or publication type
Needs ranking: A qualitative study using a participatory approach	Wrong intervention
Novel school-based health intervention program, A step toward early diabetes prevention	Wrong intervention
Nudging Our Way to a Healthier Population: The Effect of Calorie Labeling and Self-Control on Menu Choices of Emerging Adults	Wrong study population
Nutrition quality and waste associated with the school food system: A pilot, citizen science study in an Irish secondary school	Wrong study design or publication type
Nutritional supplement products: Does the label information influence purchasing decisions for the physically active?	Wrong outcome
Opportunity and Scope of Youth Participation in Spanish Town Planning	Wrong intervention

Overweight Status and Eating Patterns Among Adolescents: Where Do Youths Stand in Comparison With the Healthy People 2010 Objectives?	Wrong outcome
Palm oil and the next generation: exploring Malaysian youth engagement	Wrong intervention
Participation in the National School Lunch Program and the School Breakfast Program	Wrong outcome
Participation, agency, and youth voice in establishing school gardens: comparing cases from Kenya and Papua New Guinea	Wrong study design or publication type
Participatory evaluation and co production-more than the sum of its parts?	Wrong study design or publication type
Participatory Mapping of Holistic Youth Well-Being: A Mixed Methods Study	Wrong intervention
Partnering with youth to map their neighborhood environments: a multilayered GIS approach	Wrong intervention
Perceived climate change risk and global green activism among young people	Wrong intervention
Perception, concern, competency: children's understanding of physical and non-physical aspects of urban environment in Iran	Wrong intervention
Photovoice: Engaging youth in rural Uganda in articulating health priorities through participatory action research	Wrong intervention
Platforming youth voices in planetary health leadership and advocacy: an untapped reservoir for changemaking	Wrong study design or publication type
Population education with rural youth: a regional experience	Wrong study design or publication type
Positive Youth Development Programs Build Civically Engaged Leaders	Wrong study design or publication type
Potato Chips, Cookies, and Candy Oh My! Public Commentary on Proposed Rules Regulating Competitive Foods	Wrong outcome
Power, agency and participatory agendas: A critical exploration of young people's engagement in participative qualitative research	Wrong intervention
Preconceptional Nutrition Interventions for Adolescent Girls and Adult Women: Global Guidelines and Gaps in Evidence and Policy with Emphasis on Micronutrients	Wrong outcome
Prefiguring sustainable futures? Young people's strategies to deal with conflicts about climate-friendly food choices and implications for transformative learning	Wrong intervention
Preventing adolescent obesity through an interdisciplinary game-based mhealth system (TeenPower)	Wrong study design or publication type
Problem Solving as an Active Ingredient in Indicated Prevention and Treatment of Youth Depression and Anxiety: An Integrative Review	Wrong study design or publication type

Promoting climate change transformation with young people in Brazil: participatory action research through a looping approach	Wrong intervention
Prospects for qualitative GIS at the intersection of youth development and participatory urban planning	Wrong intervention
Public attitudes to government intervention to regulate food advertising, especially to children	Wrong study population
Pupil and Teacher Perceptions of Community Action: An English Context	Wrong intervention
Reactions to the U.S. Preventive Services Task Force guidelines for the management of adolescent obesity: A multi-informant qualitative approach	Wrong intervention
Recording of Children and Young People's Views in Contact Decision-Making	Wrong intervention
Reducing childhood obesity through U.S. federal policy: a microsimulation analysis	Wrong outcome
Reducing sugary drink intake through youth empowerment: results from a pilot-site randomized study	Wrong intervention
Regional advocacy workshop on noncommunicable disease priorities among children and adolescents in the Eastern Mediterranean Region	Wrong intervention
Regional Overview on Maternal Nutrition and Examples of Health System Programme and Policy Responses: Asia and the Pacific...International Symposium on Understanding the Double Burden of Malnutrition for Effective Interventions organized by the International Atomic Energy Agency (IAEA) in cooperation with United Nations Children,Äôs Fund (UNICEF) and World Health Organization (WHO)	Wrong outcome
RESEARCH AND PRACTICE. Development of the Policy Indicator Checklist: A Tool to Identify and Measure Policies for Calorie-Dense Foods and Sugar-Sweetened Beverages Across Multiple Settings	Wrong intervention
Restoring Our Roots: Land-Based Community by and for Indigenous Youth	Wrong intervention
Rhetoric, reality and resilience: overcoming obstacles to young people's participation in development	Wrong intervention
Rural Youths' Participation in Agriculture: Prospects, Challenges and the Implications for Policy in Nigeria	Wrong intervention
School Canteens in Selected Areas in Indonesia: A Situation Analysis	Wrong outcome
School Citizenship Education through YPAR: What Works? A Mixed-Methods Study in Italy	Wrong intervention
School Wellness Policies: Opportunities for Change	Wrong study design or publication type
State school nutrition and physical activity policy environments and youth obesity	Wrong outcome

Statewide Policies on Competitive School Foods and Beverages: Broadening the Scope of School-Based Antiobesity Efforts	Wrong study design or publication type
Sugar-Sweetened Beverage, Obesity, and Type 2 Diabetes in Children and Adolescents: Policies, Taxation, and Programs	Wrong outcome
Supporting youth-led community geography on the impacts of neighbourhood social infrastructure on young people's lives: a case study from East Scarborough, Canada	Wrong intervention
Tackling obesity among black adolescents using youth as research partners	Wrong study design or publication type
Talking About Youth Health: An Example of Engaging Youth to Improve Child and Youth Health Indicators in British Columbia, Canada	Wrong intervention
Teacher and youth priorities for education for environmental sustainability: A co-created manifesto	Wrong intervention
Teens as teachers in the garden: Effects on youth vegetable intake and preference	Wrong intervention
Texting for Health: The Use of Participatory Methods to Develop Healthy Lifestyle Messages for Teens	Wrong intervention
The adolescents, life context, and school project: Youth voice and civic participation	Wrong intervention
The BALANCE nutrition education intervention for adolescents with ASD: A formative study in a school setting	Wrong intervention
The Colorado Trust's Healthy Communities Initiative: Results and Lessons for Comprehensive Community Initiatives	Wrong outcome
The community development for healthy children initiative	Wrong intervention
The Conceptual Framework for the International Food Policy Study: Evaluating the Population-Level Impact of Food Policy	Wrong outcome
The diffusion of youth-led participatory research in urban schools: the role of the prevention support system in implementation and sustainability	Wrong intervention
The framing of environmental citizenship and youth participation in the Fridays for Future Movement in Finland	Wrong intervention
The healthy afterschool activity and nutrition documentation instrument	Wrong study population
The impact of the availability of school vending machines on eating behavior during lunch: the Youth Physical Activity and Nutrition Survey	Wrong outcome
The Mauli Ola Study: A Unique Academic-Community Partnership With MA'O Organic Farms to Understand and Address Health Inequities Among Native Hawaiians and Other Pacific Islanders in Hawai'i	Wrong intervention
The Michigan Healthy School Action Tools Process Generates Improvements in School Nutrition Policies and Practices, and Student Dietary Intake	Wrong outcome

The obesity system maps approach by portuguese adolescents- CO-CREATE project	Wrong study design or publication type
The OzHarvest Nourish Program: An evaluation of a hospitality-based program to support employment for young Australians	Wrong intervention
The prepared first responder: Engaging youth advocates at school, home and in the community	Wrong study design or publication type
The Regional Australia Summit: An Inclusive Approach to Policy Development	Wrong intervention
The role and impact of student leadership on participants in a healthy eating and physical activity programme	Wrong study population
The Social Sustainable City: How to Involve Children in Designing and Planning for Urban Childhoods?	Wrong intervention
The unheard voices of youth in urban planning: using social capital as a theoretical lens in Sunshine Coast, Australia	Wrong intervention
The wider health and wellbeing needs of those accessing paediatric care in England: engaging with the hidden voices of children and young people	Wrong study design or publication type
Towards responsive policy and actions to address non-communicable disease risks amongst adolescents in Indonesia: insights from key stakeholders	Wrong study population
Traffic light food labelling in schools and beyond	Wrong outcome
Turning Points: Canadians from Coast to Coast Set a New Course for Healthy Child and Youth Development. The National Goals for Healthy Child and Youth Development = Points Tournants: Les Canadiens et les Canadiennes d'un ocean a l'autre tracent une nouvelle voie pour le developpement sain des enfants et des adolescents. Les objectifs nationaux pour le developpement sain des enfants et des adolescents	Wrong study design or publication type
Understanding how consumers categorise nutritional labels: A consumer derived typology for front-of-pack nutrition labelling	Wrong study population
Using implementation research to improve programs targeting adolescents- experiences from Indonesia and Bangladesh	Wrong study design or publication type
Views of children and parents on limiting unhealthy food, drink and alcohol sponsorship of elite and children's sports	Wrong outcome
Visioning a Food System for an Equitable Transition towards Sustainable Diets, A South African Perspective	Wrong outcome
What determines public support of obesity prevention?	Wrong outcome
Why do the public support or oppose obesity prevention regulations? Results from a South Australian population survey	Wrong study population
With us, not to us Towards policy and program development in partnership with children	Wrong intervention
Young People and Their Engagement with Health-Related Social Media: New Perspectives	Wrong intervention
Young People's Experiences of Non-Broadcast Advertising of Unhealthy Food	Wrong study design or publication type

Youth activists for our health	Wrong study design or publication type
Youth advocacy for obesity prevention: The next wave of social change for health	Wrong outcome
Youth and climate justice: Representations of young people in action for sustainable futures	wrong study design or publication type
Youth and family members make meaningful contributions to a randomized-controlled trial: YouthCan IMPACT	Wrong intervention
Youth as Interviewers: Methods and Findings of Participatory Peer Interviews in a Youth Garden Project	Wrong study population
Youth attitudes towards goals of a new sustainable development agenda	Wrong intervention
Youth Can! Results of a Pilot Trial to Improve the School Food Environment	Wrong study population
Youth e-participation as a pillar of sustainable societies	Wrong intervention
Youth for health: Engaging Indian youth for prevention and control of NCDs	Wrong study design or publication type
Youth Goals? Youth Agency and the Sustainable Development Goals	Wrong intervention
Youth Participation for Sustainable Value Creation: The Role and Prioritization of SDGs	Wrong intervention
Youth Participation in Europe: Evidence, Opportunities and Challenges in Various Settings	Wrong study design or publication type
Youth perspectives on health & wellness: A focus group study	Wrong study design or publication type
Youth Perspectives on Soft Drinks after the Introduction of the Uk Soft Drinks Industry Levy: A Focus Group Study Using Reflexive Thematic Analysis	Wrong study design or publication type
Youth policy monitoring as a tool for developing social sustainability in local municipality	Wrong intervention

Table S3. Data extraction of included studies – Study characteristics (n=70)

	STUDY CHARACTERISTICS				
**No.**	**1st Author + year of publication**	**Country of origin**	**Study title**	**Study design**	**Socio-demographic characteristics reported (age, gender, socio-economic status, ethnicity)**
1	Addis & Murphy 2019	Wales, United Kingdom	'There is such a thing as too healthy!' The impact of minimum nutritional guidelines on school food practices in secondary schools	Qualitative	Sample size n=52<br>Focus groups n=7 with 6-10 participants in each<br>Age: N/A. From years 8 and 10 of mixed gender within 4 secondary schools within the same district in Wales. No further demographic characteristics reported.
2	Akom et al. 2016	United States of America	Youth Participatory Action Research (YPAR) 2.0: how technological innovation and digital organizing sparked a food revolution in East Oakland	Mixed-methods	Sample size n=90 youth over 3 years participated in the program. 30 students participated in interviews, demographics are:<br>Gender: Females n=15 (50%), Males n= 15 (50%)<br>Ethnicity: African American n=18 (60%), Latin American n=11 (37%), Iranian-American n=1 (0.3%),<br>Education: Grades 9-12. Grade 9 n=6 (20%), Grade 10 n=11 (37%), Grade 11 n=7 (23%), Grade 12 n=6 (20%).<br>Age and SES not reported.
3	Altares et al. 2022	United States of America	Cultivating community change to promote food access and healthy eating through participatory action research with youth	Qualitative	Participant demographics provided via email from lead author - not reported in manuscript.<br>Sample size n=20<br>Age: 15-16yrs. 60% Grade 10 (approx. 15yrs) n=12, 40% Grade 11 (approx. 16 yrs) n=8<br>Gender: 60% female (12/20), 5% non-binary n=1, 35% male n=7<br>Ethnicity: 95% Latino/ Hispanic (n=19), 5% Black (n=1)<br>Recruitment area: Globeville Elyria-Swansea (GES) is a urban, low-income neighborhood, predominantly Hispanic/Latinx and categorised as a food desert.<br>Student advisory board at a local high school was formed, consisting of 5 students.

	STUDY CHARACTERISTICS				
**No.**	**1st Author + year of publication**	**Country of origin**	**Study title**	**Study design**	**Socio-demographic characteristics reported (age, gender, socio-economic status, ethnicity)**
4	Anselma et al. 2023<br><br>Anselma et al. 2020<br><br>Anselma et al. 2019<br><br>Anselma et al. 2019<br><br>Anselma et al 2018	Netherlands	How to Evaluate the Effectiveness of Health Promotion Actions Developed Through Youth-Centered Participatory Action Research (2023)<br><br>Not Only Adults Can Make Good Decisions, We as Children Can Do That as Well" Evaluating the Process of the Youth-Led Participatory Action Research 'Kids in Action (2020)<br><br>Co-designing obesity prevention interventions together with children: Intervention mapping meets youth-led participatory action research (2019)<br><br>Kids in Action: the protocol of a Youth Participatory Action Research project to promote physical activity and dietary behaviour (2019)<br><br>Determinants of Child Health Behaviors in a Disadvantaged Area from a Community Perspective: A Participatory Needs Assessment (2018)	Cluster-randomised controlled trial (2023)<br><br>Qualitative - Process evaluation (2020)<br><br>Protocol	2023 paper: Sample size n=700 at baseline/T0 (Intervention n=397, control n=303). T1 n=538 (intervention n=266, control n=272), T2 n=632 (intervention n=384, control n=248)<br>Age Range: 9 to 12 years old, mean age 10.6 years.<br><br>Socioeconomic Background: The children were from a lower-socioeconomic neighbourhood, 4 control schools and 4 intervention schools.<br>School Setting: Primary school children from the three highest grades in both intervention and control schools.<br><br>Year 1 Action teams: total sample n=25. total of 6-7 children in each school (female n=12, male n=13). Year 2: female n=11, male =9, (6 boys were from previous year). 1 school did not participate in year 2 but rejoined in 3rd year. Year 3: youth council total = 13, female n=4, male n=9. 1 school did not recruit any students for final year.<br><br>Process evaluation (2020):<br>Eight focus groups with children (N = 40) and eight interviews with community partners (N = 11) were held at the start and end of the 2nd year.
5	Asada et al. 2017	United States of America	High School Students' Recommendations to Improve School Food Environments: Insights From a Critical Stakeholder Group	Mixed methods	Sample size n= 15 (5 focus groups) from high schools across 9 states. the majority of schools were urban, high free and reduced-price eligible, Hispanic, and located in the western United States. Focus groups were held during the Youth for Healthy Schools conference convened by the Funders' Collaborative on Youth<br>Organizing (FCYO) in Los Angeles, California in July 2015 - a non-profit organisation that aims to increase youth capacity in civic organisation advocacy across several domains including health and wellness<br>Age: not reported<br>Ethnicity: 80% Hispanic, 13% Black, 7% diverse<br>Follow up interviews n=9.<br>Socio-economic status:<br>60% enrolled in Urban population, 0% rural, 40% suburb,<br>Free and reduced-price lunch eligibility (tertiles): 67% high, 33% Middle, 0% Low (Categories defined by National Center for Education

	STUDY CHARACTERISTICS				
**No.**	**1st Author + year of publication**	**Country of origin**	**Study title**	**Study design**	**Socio-demographic characteristics reported (age, gender, socio-economic status, ethnicity)**
					Statistics Common Core of Data (CCD) 2013-2014. National Center for Education Statistics. Common Core of Data (CCD) 2013-2014. https://nces.ed.gov/ccd/ Accessed September 1, 2015
6	Balvanz et al. 2016	United States of America	From Voice to Choice: African American Youth Examine Childhood Obesity in Rural North Carolina.	Qualitative	Sample size n=7<br>Gender: 100% female<br>Ethnicity: 100% African American<br><br>Setting: in a small, low-income, rural community in North Carolina with a predominately African American population.23 Per capita income in the community was less than $12,000, and is located in a county in the lowest quintile of per capita income in North Carolina<br><br>Recruitment: partnered with youth who attended an afterschool program
7	Bosco et al. 2017	United States of America	Food Journeys: Place, Mobility, and the Everyday Food Practices of Young People	Qualitative	Survey total Sample n=38<br>Age: 16-17 years. Mean age not reported.<br>Gender (survey): 26% female, 74% male<br>Ethnicity: 81% Latino, 5% Black<br>Socio-economic status: 78% foreign born parents, 84$ live in rental housing.<br>Recruitment site: students enrolled in a high school GIS class from a urban, multicultural, and lower income community in San Diego, California.<br><br>Photovoice exercise total sample n=19.<br>Study focused on 3 students that participated via pseudonyms to show 'representative' engagement amongst with food in their community. Alicia age 16, ethnicity = Latina, full-time student. Speaks Spanish at home. Miguel aged 16yrs, works part-time, speaks Spanish and English. Carlos is 17yrs old, born in Mexico, works part-time and lives in a 6-person household and has a young child who lives with his girlfriend.

	STUDY CHARACTERISTICS				
**No.**	**1st Author + year of publication**	**Country of origin**	**Study title**	**Study design**	**Socio-demographic characteristics reported (age, gender, socio-economic status, ethnicity)**
8	Breckwich Váquez et al. 2007<br><br>Minkler 2009	United States of America	Addressing Food Security Through Public Policy Action in a Community-Based Participatory Research Partnership<br><br>Linking Science and Policy Through Community-Based Participatory Research to Study and Address Health Disparities	Qualtiative	Sample size: N/A<br>Recruitment: Youth from local Bayview high schools were recruited (about 6 to 8 per year) to join LEJ's Good Neighbor Project as paid interns working 5 to 10 hours per week.<br><br>Ethicity: "youth recruited were largely African American, Asian American, and Pacific Islander American" (p.344).<br>No further demographic details reported.
9	Browne et al. 2020	Republic of Ireland	'We know what we should eat but we don't...': a qualitative study in Irish secondary schools	Qualitative	Total sample of students in focus groups n=54 over 7 peer led focus groups<br>Age: mean 16yrs<br>Gender: male n=27, female n=27<br>Total sample of peer moderators n=17<br>Gender: male n=8, female n=9<br>Recruitment: Six Irish public secondary schools
10	Browne et al. 2023	Republic of Ireland	Nutrition quality and food and packaging waste associated with theschool food system: A pilot, citizen science study in an Irishsecondary school	Mixed methods	Total sample n=11, (focus group n=7)<br>Age: 15 -17 years. Mean age not reported.<br>Gender: 100% males<br>Recruitment: a single-sex urban secondary school in Ireland. Students were eligible if they were in their "Transition year" which is "an academic year that lies outside of the main exam-orientated curriculum in Irish secondary schools, in which students have a more flexible timetable to allow for periods of work shadowing, and project- and community-oriented work"(p.2312)
11	Callaghan et al. 2010	Canada	Healthier Snacks in School Vending Machines: A Pilot Project in Four Ontario High Schools	Mixed-methods pilot study	Total sample (focus groups) n= 40 participants;<br>Age: 60% in year 12, and the others were in grades 9 to 11.<br>Gender: 65% were girls.
12	de Carvalho et al. 2021	Brazil	Participatory Geographic Information Systems (PGIS) to assess water, energy and food availability in a vulnerable community in Guarulhos (Brazil)	participatory mixed methods mapping case study	total sample n=22 that completed the 'Participative Geographic Information System for Environmental Health' course<br>Maptionnaire survey n=16<br>Age: 14–17, no other demographics available<br>Study area: Novo Recreio, Brazil -<br><br>This community has a socioenvironmental vulnerability index (SVI) of 0.374, which indicates higher vulnerability relative to the wider municipality of Guarulhos.

	STUDY CHARACTERISTICS				
No.	1st Author + year of publication	Country of origin	Study title	Study design	Socio-demographic characteristics reported (age, gender, socio-economic status, ethnicity)
					Novo Recreio has limited access to fresh food, including fruit and vegetables.
13	do Valle Santos et al. 2019	Brazil	Vertical Gardens: Sustainability, Youth Participation, and the Promotion of Change in a Socio-Economically Vulnerable Community in Brazil	Qualitative	Total sample n=23 adolescents<br>Age: 11-14 yrs<br>Gender: female n=4, male n= 19<br>Setting: The Youth Centre (YC) serves children and adolescents aged 6 to 14 who face vulnerabilities such as rights violations, disabilities, or dependence on government income. Its mission is to provide social protection, develop potential, support autonomy and citizenship, and strengthen family and community connections. The YC creates a space that addresses the interests and needs of its young participants.<br>'Socio-economically vulnerable adolescents'. Approximately 80% of the population is defined as low-income residents, and most of the remainder lives in extreme poverty.
14	Elliott 2017	Canada	Knowledge needs and the 'savvy' child: teenager perspectives on banning food marketing to children	Qualitative	Total sample n=30 (6 in each focus group)<br>Age: 12-14 years (2x focus groups aged 12, 2x aged 13, 1x aged 14), mean age = 12.8yrs<br>Gender: female n=18, male =12<br>Recruitment: Junior High School in Calgary
15	Elliott et al. 2022	Canada	Food marketing to teenagers: Examining the power and platforms of food and beverage marketing in Canada	mixed-methods observational pilot study	Sample size n=62 (5 excluded due to incomplete data)<br>Age range: 13–17, mean age 14 (out of 57 remaining participants)<br>Gender: Of the 57 participants remaining, girls n=39, boys n=17, gender non-conforming n=1<br>Setting: Participants were recruited from schools, community groups and sports teams in [Calgary, AB, Canada] to take part in the study between January and May 2021. The study was conducted during the Covid-19 pandemic therefore engagement with food marketing likely more common on digital platforms.
16	Elliott et al. 2023	Canada	Tracking teen food marketing: Participatory research to examine persuasive power and platforms of exposure	Mixed method	Total sample n=367, 58 excluded due to invalid data, final sample n=309<br>Age range: 13–17yrs, mean age 15.5yrs<br>Gender: girls n=192/309 (62%), boys n=96/309 (31%), gender non-confirming n=21/309 (7%)

	STUDY CHARACTERISTICS				
**No.**	**1st Author + year of publication**	**Country of origin**	**Study title**	**Study design**	**Socio-demographic characteristics reported (age, gender, socio-economic status, ethnicity)**
17	Findholt et al. 2011	United States of America	Photovoice Engages Rural Youth in Childhood Obesity Prevention	Qualitative	Sample size n=6,<br>Age: 15–18 years of age,<br>Gender: female n=4, male n=2<br>Ethnicity: 100% white<br>Recruitment: one from each community in Union County, recruited through flyers in high schools and school staff informing students about the opportunity<br>Eligibility: student had to have lived in his or her community for at least 8 years and attended the local elementary school. Thus, the students had inside knowledge of what it was like to be a child in their communities.<br>Setting: Union County is an agricultural region in northeast Oregon that, at the time of the study, had a population of 24,753 people (U.S. Census Bureau, 2007). Most of the residents were White (94.0%) and the median household income was well below the state average
18	Fleming et al. 2023	Global - 18 countries (Ghana, Zimbabwe, Sri Lanka, UK, Turkey, Indonesia, Guatemala, China, Nepal, Netherlands, Cambodia, Bangladesh, Palestine, Egypt, Ethiopia, Mexico, Kenya and Nigeria)	Fix my food: An urgent call to action from adolescents on how they experience and want to see change in their food systems	Qualitative	Sample size n= 640 adolescents<br>Age: 10–19 years across the 18 countries.<br>Gender: female n= 383/640 (59.8%), male n=256 (40%), other n=1 (0.16%) with an even spread between age groupings from 10–17 years and smaller number of participants aged 18–19 years (n= 88). China and Indonesia had the largest number of participants and gender representation differed in each workshop.<br>More details provided in table 1 (p. 2300)
19	Freire et al. 2017	Ecuador	A qualitative study of consumer perceptions and use of traffic light food labelling in Ecuador	Qualitative	Sample size n=178<br>Age range: 5-64 years. Focus groups were organised by sex and age group including 10–14, 15–19yrs
20	Frerichs et al. 2012	United States of America	SaludableOmaha: Development of a Youth Advocacy Initiative to Increase Community Readiness for Obesity Prevention, 2011–2012	pre-post study	14 participated in the program through the first 2 phases. Three dropped out for personal reasons.<br>Setting: a high school in Omaha, Nebraska institutionalized a youth

	STUDY CHARACTERISTICS				
**No.**	**1st Author + year of publication**	**Country of origin**	**Study title**	**Study design**	**Socio-demographic characteristics reported (age, gender, socio-economic status, ethnicity)**
	Frerichs et al. 2015		Process and Outcomes From a Youth-Led Campaign to Address Healthy Eating in an Urban High School		advocacy obesity prevention initiative, SaludableOmaha, within a service-learning course. 15 students from the course had an interest in addressing health and social issues in their community.<br>The survey sample size was n = 89 and n = 74 at baseline and post-campaign.<br>No further demographic details available<br>55.8% of the population of Omaha is Hispanic, and poverty rates of the Latino population in Omaha are high (2012 paper)
21	Frerichs et al. 2018	United States of America	Development of a Systems Science Curriculum to Engage Rural African American Teens in Understanding and<br>Addressing Childhood Obesity Prevention	Mixed methods	Total sample n=21 over 4 workshops<br>Age: 13-17yrs, mean age: 16.3 (1.4) years<br>Gender: Female n=13 (66.7%), male n=8 (33.3%)<br>Ethnicity: African American<br><br>Interviews n=7 youth post intervention<br><br>Setting: North Carolina County, in which African Americans comprise 57% of the population. The county is rural, with a population density of 112 persons per square mile. This county also has the highest unemployment rate in North Carolina and 24% of African American residents live in households with incomes below federal poverty levels.
22	Gangrade et al. 2023	United States of America	Examining the feasibility of a youth advocacy program promoting healthy snacking in New York City: a mixed-methods process evaluation	Mixed-methods process evaluation	Total sample n=38, 2 dropped out<br>Focus groups n= 28.<br>Age: 12-18 years, mean age = 14.7yrs,<br>Gender: females, n=20 (55.5%), males n=16 (44.4%)<br>Ethnicity: Black/African American n=22 (61.1%), Hispanic, Latino or Spanish origin n=11 (30.6%), Asian n=2 (2.8%, mixed n=1 (5.6%)<br>Eligibility: spoke English<br><br>Setting: recruited at a Boys and Girls Club in New York City . It's an organisation that offers after-school programing focused on academics, leadership and health to its 2 million youth members who are predominantly from low-income backgrounds
23	Hackett et al. 2015	United States of America	Examining childhood obesity and the environment of a segregated, lower-income US suburb	Qualitative	Sample size n=9<br>Age: 15-17yrs<br>Gender: female n=5, male n=4<br>Ethnicity: African-American n=8, Latina n=4

	STUDY CHARACTERISTICS				
**No.**	**1st Author + year of publication**	**Country of origin**	**Study title**	**Study design**	**Socio-demographic characteristics reported (age, gender, socio-economic status, ethnicity)**
					Recruitment: all were employed by the Roosevelt Community Farmer's Market for one-three years
24	Hermans et al. 2017	Netherlands	Adolescents' responses to a school-Based Prevention Program Promoting healthy eating at school	Mixed method	Total sample focus groups n=42,<br>Age: 13-16yrs<br>Gender: female n=23, male n=17<br>Educational level: Low (VMBO) = 38%, Medium (HAVO) = 29%, High (VWO) = 33%<br><br>surveys n=133<br>Age: 12-19yrs<br>female n=72, male n=61<br>Educational level: Low (VMBO) = 47%, Medium (HAVO) = 23%, High (VWO) = 30%<br>recruited using snowball sampling<br><br>VMBO is equal to vocational training level; HAVO is equal to senior secondary general education level; VWO is equal to pre-university education level.
25	Hinkle et al. 2018	United States of America	How Food & Fitness Community Partnerships Successfully Engaged Youth	Qualitative	Total sample (interviews) n=73 between 2008-2016.<br>Across all Food and Fitness partnerships, a total of 100 youth were involved in core (e.g., planning, implementation, evaluation) partnership activities.<br>Age: 63% were between 16 and 18 years of age, 19.2% were between 19 and 21 years, 9.6% were between 22 and 24 years, and 8.2% were between 13 and 15 years of age<br>Gender: approximately half were male/ female<br>Ethnicity: The majority of the youth identified as African American/Black (32.9%), 21.9% as White/Caucasian, 17.8% as Hispanic, 12.3% as Asian, 12.3% as Native American, and 2.7% as Other.<br>"Over the course of the 9 years, thousands of young people participated in F&F activities" (p.39s)
26	James et al. 1996	United States of America	Using Focus Group Interviews to Understand School Meal Choices	Qualitative	Total sample n=35<br>Age: Ninth grade students<br>Gender: female n=19, male n=16<br>Ethnicity: white n=19, African American n=11, Hispanic n=3, Asian n=2

	STUDY CHARACTERISTICS				
**No.**	**1st Author + year of publication**	**Country of origin**	**Study title**	**Study design**	**Socio-demographic characteristics reported (age, gender, socio-economic status, ethnicity)**
					Setting: focus groups were conducted in five distinct geographical areas in Florida: Jacksonville, Ft. Lauderdale, Sarasota, Quincy, and Gainesville of different socio-economic and ethnic backgrounds
27	Jáuregui et al. 2023	Mexico	Design of a communication strategy to promote the use of warning labels among Mexican children and adolescents	Mixed methods four-phase study: 1) formative research; 2) co-creation workshops with children and adolescents; 3) design of the communication strategy; and 4) pilot testing the strategy.	Sample size: Phase 1: formative research focus groups n=179 (with children, adolescents and parent/caregivers),<br>Adolescents n=75, mean age 14 (1.0)<br>Gender: female = 61.3%, male =38.7%<br>Socio-economic level: low = 42.7%, high = 57.3%<br><br>phase 2 co-creation workshops with children and adolescents<br>Age range 10-14 yrs, mean, age 12.5yrs (1.0),<br>gender: female = 57.6%, male =42.4%,<br>Socio-economic level: low =36.4%, high =23.1%<br><br>Phase 3 pilot test: adolescents n=19<br>mean age = 12.9yr (1.0)<br>gender: female =47.4%, male =52.6%<br>Socio-economic level: low = 47.4%, High = 52.6%<br><br>school children n=10,<br>Age 6-12 years, mean age 10.1 (0.7)<br>Gender: female = 57.9%, male =42.1%<br>Socio-economic level: low =36.8%, high =63.2%<br>phase 2: co-creation workshops with children and adolescents n=33 aged 10-14yrs. stage 4 pilot study n=52<br><br>Recruitment: Low SES participants were recruited at health centres in the city of Cuernavaca and through community leaders in rural areas of the state of Morelos. Those with high SES were recruited through private schools in Cuernavaca.
28	Johnson et al. 2017	United States of America	Food in My Neighborhood: Exploring the Food Environment through Photovoice with Urban, African American Youth	Qualitative	Sample size n=17,<br>Age: 10–13yrs, mean age 11.1 years<br>Gender: female n=10, male n= 7<br>Ethnicity: 100% African American, which is representative of the community<br>Setting: Baltimore City<br>Recruited: Summer camp

	STUDY CHARACTERISTICS				
**No.**	**1st Author + year of publication**	**Country of origin**	**Study title**	**Study design**	**Socio-demographic characteristics reported (age, gender, socio-economic status, ethnicity)**
29	Jones et al. 2012	England, UK	Engaging Secondary School Students in Food-Related Citizenship: Achievements and Challenges of a Multi-Component Programme	Two stage, mixed methods study	Sample size n= 2054 at baseline, follow up n= 1926 students.<br>Study duration 18-24 months.<br>Age: Years 7, 8, 9 and 10<br>The profiles of the two groups were similar in terms of the mean respondents per school (baseline: 71.6, SD: 33.5; follow up 69.4, SD: 29.8); gender (baseline: 54.2% female; follow up: 52.2% female); and take up of school meals (baseline: 30.3%, follow up: 30.1% for purchase of school food 5 days a week).<br><br>Recruitment: 31 secondary schools were recruited to the FFLP flagship programme between September 2007 and September 2008. Of these, 24 were sampled to take part in the evaluation.<br>School size ranged from 401 to 1809 pupils (average: 978; SD: 323).<br>Ethnicity: similar to England School Census figures for "White British" origin (88% compared to 86%).<br>Socio-economic status: Free school meal (FSM) entitlement (an indicator of socio-economic deprivation) suggested that the sample of schools reflected a range of socio-economic contexts: the highest FSM eligibility was 55% and the lowest was 2%.
30	Kebbe et al. 2019	Canada	End-user perspectives to inform policy and program decisions: a qualitative and quantitative content analysis of lifestyle treatment recommendations<br>by adolescents with obesity	Qualitative	Sample size n=19<br>Age: 13-17 years old, mean age 15.1 (SD: 1.7)<br>Gender: female n=11 (57.9%), male n=8 (42.1%)<br>Ethnicity: Caucasian n=13 (68.4%), non-Caucasian n=6 (31.6%)<br>Socio-economic status: 52.6% of parents attended at least college or university, 72.2% earned >$50,000/y CDN<br>Recruitment: two urban multidisciplinary weight management clinics: Anglophones from the Paediatric Centre for Weight and Health (Edmonton) and Francophones from the Centre for Healthy Active Living (Ottawa). All participants lived with obesity n=4 (21.2%) or severe obesity n=15 (78.9%)
31	Kim et al. 2019	United States of America	Native American Youth Citizen Scientists Uncovering Community Health<br>and Food Security Prioritie	Mixed methods participatory action reseach	Sample size n=12 youth<br>The survey concluded with 212 responses from community (33.8% aged 13-18yrs)<br>Age: 13-18 years<br>ethnicity: Indigenous - 100% Native American from the Karuk Tribe<br>Setting: rural/remote northern California.

	STUDY CHARACTERISTICS				
**No.**	**1st Author + year of publication**	**Country of origin**	**Study title**	**Study design**	**Socio-demographic characteristics reported (age, gender, socio-economic status, ethnicity)**
32	Kontak et al. 2022	Canada	Peering in: youth perspectives on Health Promoting Schools and youth engagement in Nova Scotia, Canada	Qualitative	Peer researchers: Sample size n=10,<br>Age: 14–16years,<br>Gender: female n=8, male n=2<br>ethnicity: French Acadian n=2, New Immigrants n=8, white Canadians n=2<br>Youth interviewed n=23<br>Age 12-16 years<br>Year group: grade 7 n=6, grade 8n=3, grade 9n=7 grade 10 n=7. Other demographic information of participants was not collected.<br>Eligibility: A student in grades 7–10 and attending a public school in Nova Scotia, Canada
33	Leung et al. 2017	United States of America	Voices Through Cameras: Using Photovoice to Explore Food Justice Issues With Minority Youth in East Harlem, New York	Qualitative	Sample size n=12<br>Age: 11 -14 years,<br>Gender: female n=7, male n=5<br>ethnicity: Hispanic or Black/African American n=11, White n=1<br>Setting: NYC nonprofit organizations focused on providing programming and assistance for children living in poverty.
34	Lilo et al. 2021<br><br><br><br>Lilo et al. 2023	United States of America	"OMG, I Get Like 100 Teaspoons of Sugar a Day!" Rural Teens' Grasp of Their Beverage Consumption Habits<br><br>Development and Piloting of the Enhanced Integrated Behavioral Model to Frame a Social Marketing Campaigns for Teens	Qualitative - post-hoc analysis as part of a program evaluation<br><br>Mixed methods	Campaign evaluation - Sample size n=27<br>Age: not reported - Author said details reported elsewhere and referenced their thesis but not publicly available.<br>Setting: rural New Mexico high school<br>Level of participation in the campaign:<br>high-participators n=8, low-participators n=9, and non-participators n=9<br>The campaign took place in a small high school (approximately 240 students), in an underserved, frontier community in New Mexico, where 96% of the students were Native American (68%) or Hispanic (28%).
35	Madrigal et al. 2014	United States of America	Health in my community: conducting and evaluating PhotoVoice as a tool to promote environmental health and leadership among Latino/a youth	Qualitative	Sample size n=16 with n=1 dropout<br>Age: 14-18 years<br>Ethnicity: all Latino of Mexican descent<br>Recruitment: members of the Youth Community Council in Salinas, CA (total members)<br>Photovoice project sample size n=16<br>Age: 14-18 years<br>Gender: female n=9, boys n=7<br>Ethnicity: all Latino of Mexican descent

	STUDY CHARACTERISTICS				
**No.**	**1st Author + year of publication**	**Country of origin**	**Study title**	**Study design**	**Socio-demographic characteristics reported (age, gender, socio-economic status, ethnicity)**
36	Mansfield et al. 2024	United States of America	High School Student and Caregiver Preferred Communication Method Regarding School Meals: A Qualitative Approach	Qualitative	Sample size n=47 across 7 focus groups<br>Ages: 14-21, grades 9-12<br>ethnicity: Black or African American n=67%, identified as Hispanic/Latinx n=6.5% , and the remaining 27% were split equally among Biracial or Multiracial, Asian/Pacific Islander, and non-Hispanic white.<br>Setting: 4 high schools in a large, urban city.<br>No further demographics reported.<br>"we focused on schools serving high school students of lower income families" (p.54)
37	Martin et al. 2018	Republic of Ireland	Including the voices of children and young people in health policy development: An Irish perspective	Qualitative	Two consultations were held – one with 48 children between the ages of 8 and 12 and the other with 34 young people aged 13–17 years.<br>"Considerable focus was placed on ensuring the participation of a balanced representation of children and young people from socio-economic, gender, ethnic and geographic perspectives." - However not reported
38	Sprague Martinez et al. 2020	United States of America	Changing The Face Of Health Care Delivery: The Importance Of Youth Participation	Qualititative	Youth researchers n=12<br>Age: 13-18 years<br>Ethnicity: all Black and Latinx youth<br>Survey responders n=93<br>Age: 65% between 10-18yrs. Mean age: 19.86years<br>Gender: female =58%, male 41%, gender non-conforming =1%<br>Ethnicity: Black or African American 73.5%, Hispanic or Latinx = 44%, Asian or Asian American = 14.5%, White =12%
39	Miller et al. 2021	Australia	Adolescents report low opposition towards policy options to reduce consumption of sugary drinks	Cross-sectional survey	Sample size n= 9102,<br>Age: 12-17 years.<br>Proportion of age: 16-17 (32%), aged 15yrs (27%), aged 14 yrs (25%), aged 16 (16%).<br>Gender: 48% female, 52% male.<br>Income: 40% live in level of least disadvantage, 38% mid disadvantage, 22% most disadvantaged<br>Recruitment: schools were stratified by the three education sectors (government, Catholic and independent) and randomly selected for inclusion.

	STUDY CHARACTERISTICS				
**No.**	**1st Author + year of publication**	**Country of origin**	**Study title**	**Study design**	**Socio-demographic characteristics reported (age, gender, socio-economic status, ethnicity)**
40	Millstein et al. 2016 a) &<br><br>Millstein et al. 2016 b)<br><br>Linton et al. 2014	United States of America	A) Development of measures to evaluate youth advocacy for obesity prevention<br><br>B) A pilot study evaluating the effects of a youth advocacy program on youth readiness to advocate for environment and policy changes for obesity prevention<br><br>Youth advocacy as a tool for environmental and policy changes that support physical activity and nutrition: an evaluation study in San Diego County	Mixed methods	Sample size n= 136 across 21 advocacy groups<br>Age: 9-22, mean age = 15.3. 95% aged 11-18 years.<br>Gender: female n=98 (73.1%), male n=36 (26.9%)<br>Ethnicity: white n=19 (13%), Black n=34 (23.3%), Hispanic/ Latino(a) n=52 (35.6%), Asian/ Pacific Islander,/ Native Hawaiian n=32 (21.9%), Other n=22(15%) *participants could click more than 1 response previous advocacy experience =72.1%
41	Mosavel et al. 2018	United States of America	Using Asset Mapping to Engage Youth in Community-Based Participatory Research: The WE Project	Qualitative	Sample size n=13.<br>Age: 16-19 years, mean 16.9 years.<br>Gender: female n=6, males n=7<br>Ethnicity: Black or African American n= 11, mixed race n= 2.<br>Education: completed 10th grade n=5, completed 11th grade n=4, 12th grade n= 3, community college n=1.<br>School attended local Governor's high school n=7, public high school n=5.<br><br>Total graduate students n=9,<br>Age: 22- 40, mean age 29 yrs.<br><br>Setting: Petersburg: A disadvantaged community that experiences health inequities - 43% of families report household incomes of less than $25,000.
42	Muturi al. 2018	United States of America	Examining the role of youth empowerment in preventing adolescence obesity in low-income communities	Cross sectional	Sample size n=410 witha 66% response rate.<br>Age 11-15 years, mean age =12.8<br>Gender: females n=230 (56%), males n=176 (43%), did not disclose n=4 (1%)<br>Ethnicity: Hispanic =40%, White/ Caucasian (22%), Black/African American (16.1%), American Indians (8%), Asian (1%), other or multiple ethnicities =13%.<br>Setting: 3 US states: Kansas, Ohio and South Dakota.

	STUDY CHARACTERISTICS				
**No.**	**1st Author + year of publication**	**Country of origin**	**Study title**	**Study design**	**Socio-demographic characteristics reported (age, gender, socio-economic status, ethnicity)**
43	Necheles et al. 2007	United States of America	The Teen Photovoice Project: a pilot study to promote health through advocacy	Qualitative	Sample size n=13 who served Youth Advisory Board (YAB) of the UCLA/ RAND Center for Adolescent Health Promotion.<br>Age: 13- to 17-year-old<br>Gender: female n=11 male n=2<br>Ethnicity: African American n=9, Mexican American n=3, Asian n=1.<br>Socioeconomic status: They attended both public and parochial schools, and lived in Los Angeles area communities in which 4% to 39% of the families with children lived below the poverty level.
44	Ortega et al. 2015<br><br>Sharif 2015	United States of America	Proyecto MercadoFRESCO: A Multi-level, Community-Engaged Corner Store Intervention in East Los Angeles and Boyle Heights<br><br>Mobilizing young people in community efforts to improve the food environment: Corner store conversions in East Los Angeles	Mixed methods<br><br>Qualitative	Ortega 2015: Stores recruited n=8. Participating corner stores were near local high schools facilitating the inclusion of high school students in our community health education and social marketing activities. Students did not participate in the evaluation of this study.<br>Sharif et al. 2015:<br>Focus groups sample size n= 30 (54% of the total number of students) who were enrolled in an elective course, “Market Makeovers and Social Marketing,” at two public high schools.<br>Age: 16-17 years, mean age 16.9 years<br>Gender: female n=23 (77%), male n=7 (23%)<br>Ethnicity: 100% Latino, 23% born in the US, participants spent a mean of 14.1 years of their life in the United States. Socio-economic status: parents did not complete high school n=14<br>Setting: East Los Angeles and Boyle Heights in California. These are two neighboring, predominantly Latino communities that have high rates of overweight and obesity. Approximately 95 % Latino, almost half (48 %) foreign-born, and with approximately 85 % of residents having Mexican heritage. "These communities are food swamps with high concentrations of fast food restaurants and other venues that serve foods that are high in fat and sugar. While there are a few supermarkets, there are approximately 150 sole proprietor owned corner stores in these communities. These small stores largely sell energy-dense foods, sugar sweetened beverages, alcohol and little, if any, fresh fruits and vegetables." (p.348)
45	Pawlowski et al. 2024	Canada	Youth perspectives on community health in Nunavik: a community-engaged photovoice project	Qualitative	Sample size n=51<br>Age: 12- 18yrs (no mean given)<br>Gender: female n=25, male n=26<br>Ethnicity: Inuit and First Nations<br>Setting: 3 communities in Nunavik, northern Quebec Canada

	STUDY CHARACTERISTICS				
**No.**	**1st Author + year of publication**	**Country of origin**	**Study title**	**Study design**	**Socio-demographic characteristics reported (age, gender, socio-economic status, ethnicity)**
					Photovoice activities took place over 1 week between January and February 2020.
46	Jones et al. 2022	United Kingdom	Adolescents' perspectives on soft drinks after the introduction of the UK Soft Drinks Industry Levy: A focus group study using reflexive thematic analysis	Qualitative	Sample size = 23 across 4 focus groups from December 2018 to May 2019.<br>Age 11-14 years.<br>Gender: 15 females, 8 males<br>Recruitment: A purposive sample of 13 state-funded and fee-paying schools within Cambridgeshire and Essex were approached, with 2 schools agreeing.
47	Pettigrew et al. 2017	Australia	The types and aspects of front-of-pack food labelling schemes preferred by adults and children	Cross sectional	Sample size n = 500 children (10-19 years) + 1558 adults<br>Breakdown of children:<br>Gender: Male n=271, female n= 261<br>Socio-economic status: low SES n= 258, Medium- High SES n=274<br>BMI: underweight n=66, normal weight n=182, overweight/ obese n=109 *not all participants reported BMI data
48	Poplin et al. 2017	United States of America	Transforming Pervasive into Collaborative: Engaging Youth as Leaders with GIS through a Framework that Integrates Technologies, Storytelling, and Action	Mixed methods	Sample size n=32 (spring program n=22, spring n= 10)<br>Age range: The students participating in our study were in 6th, 7th, or 8th grade. They were between 11 and 13 years-old.<br>Gender: female n=22, male n=3 *not all participants were reported. See table 1.<br>Ethnicity: not reported - "Participants were not asked to identify their racial backgrounds; however, the group was ethnically diverse, which is in keeping with the racial demographics of these neighbourhoods." Capitol East, Capitol Park, and MLK Jr. Park neighbourhoods were selected because they have among the highest levels of resource-vulnerable and young populations in the city. In these neighbourhoods, the total population is 8,673 with significant populations of Black and African American residents: 22.7% averaged across the three neighbourhoods and Hispanic or Latino/a residents: 33.3% .<br>These neighbourhoods have a larger percentage of residents whose first language is not English.<br>Socioeconomic status: Nearly 30% of the population is living below the poverty line (for example, $16,240 for a family of two or $24,600 for a family of four).<br>Setting: Youth that attend the Boys & Girls Club in some of the lowest-income neighborhoods in Des Moines, Iowa

	STUDY CHARACTERISTICS				
**No.**	**1st Author + year of publication**	**Country of origin**	**Study title**	**Study design**	**Socio-demographic characteristics reported (age, gender, socio-economic status, ethnicity)**
49	Riggsbee et al. 2018	United States of America	More than Fast Food: Development of a Story Map to Compare Adolescent Perceptions and Observations of Their Food Environments and Related Food Behaviors	Mixed method	Survey sample size n=75<br>Age: 13–16 years, 14–15 years old (86.7%),<br>Gender: female n=34 (45.9%), male n=40 (54.1%)<br>Ethnicity: White (non-Hispanic) n=61 (81.3%), Black (non-Hispanic) n= 4 (5.3%), other (including bi-racial and Hispanic and Latino) n=10 (13.4%)<br>Twelve percent of the sample reported free or reduced lunch status; 29.3% chose not to answer or reported not knowing.<br><br>Focus groups (n = 30 across 5 groups) subset of survey participants. The Prong 2 sample was similar demographically to Prong 1; 80% reported being white non-Hispanic, Freshmen (86.7%), and 14 years old (73.3%). Three overarching themes emerged<br><br>Photovoice (n = 6) evaluated from October 2016 to April 2017. "The Prong 3 sample reported being white non-Hispanic (57.1%), with the remaining participants reporting being biracial and/or Hispanic, all Freshmen students (100%), and 14 years old (71.4%). 57.1% of the sample reported being male."<br>Recruitment: one high school in the southeastern United States.
50	Robledo de Dios et al. 2023	Spain	Qualitative study on food perceptions, dietary practices and healthy lifestyles in the adolescent population	Qualitative	Sample size n= 90<br>Aged 12-14 years<br>Gender: female n= 55, boys n=35 from four Secondary Schools, distributed in four municipalities.
51	Rose et al. 2021	United Kingdom	Pizza every day – why?': A survey to evaluate the impact of COVID-19 guidelines on secondary school food provision in the UK	Cross-sectional survey	252 responses: young people (16-18yrs) n=39 (16%)<br>parent n=152 (60%), staff n=61 (24%)<br>Mean age: not reported<br>Gender: not reported<br>Student ethnicity: White n=32 (83%), Egyptian and Italian n=1 (3%), Black/African/ Caribbean/ Black British n=5 (13%), White and Asian n=1 (3%)<br>Education: Secondary academy n=6 (15%), secondary school n=9 (23%), college n=17 (44%), Post-16 n=7 (18%)
52	Santilli et al. 2011	United States of America	Urban Youths Go 3000 Miles: Engaging and Supporting Young Residents to Conduct Neighborhood Asset Mapping	Mixed methods	7 urban youths (14–19 years) facing socioeconomic or academic barriers to postgraduation employment<br>Ethnicity: "The youths and field captains were predominantly Black and Hispanic, reflecting the demography of the neighbourhoods."

	STUDY CHARACTERISTICS				
**No.**	**1st Author + year of publication**	**Country of origin**	**Study title**	**Study design**	**Socio-demographic characteristics reported (age, gender, socio-economic status, ethnicity)**
					Setting: 6 low-resource neighbourhoods and the perimeters of 12 randomly selected schools in New Haven (urban US)
53	Shah et al. 2023<br><br>Foley et al. 2017<br><br>Shah et al. 2017	Australia	Youth Voices Creating Healthy Eating and Physically Active Environments in Schools<br><br>Evaluation of a peer education program on student leaders' energy balance-related behaviors (2017)<br><br>The Students As LifeStyle Activists (SALSA) program (2017)	Qualtitative<br><br><br>Pre-post study	SALSA Program - Peer leaders n=309 Year 10 students in 2019<br>Age: 15–16 years<br>Setting: 16 high schools in Western Sydney, Australia.<br><br>SYV - peer leaders n=84, 7 schools<br>Leadership Day evaluation survey n=51 (61% response rate, girls = 67%).<br>One school was for girls only, six were coeducational,<br>Socio-economic status: the average Index of Community Socio-Educational Advantage ICSEA of participating schools was 951 (range: 870–1,021), with the total school student enrolments ranging from 476 to 1,075.<br>Foley 2017: 415 peer leaders
54	Shaw et al. 2023	United Kingdom	Youth Voices Creating Healthy Eating and Physically Active Environments in Schools	Qualitative	Sample size n=45, across 13 online focus groups<br>Age: 12-18 years<br>Gender: female n=29, boys n=16<br>Ethnicity: White British n=36 (80%), Indian n=6 (13.3%), Filipino n=2 (4.4%), White other n=1 (2.2%)<br>Socio-economic status derived from Home neighbourhood deprivation (IMD deciles) range: 2-10. missing n=5 (11.1%), 1-4 most disadvantaged n=3 (6.6%), 5-7 n=9 (20%), 8-10 most advantaged n=28 (62.2%)<br>Family Affluence score: Median =11 (IQR 11,12; range 6 and 12)<br>Setting: most participants were based in the south of England due to convenience sampling
55	Sikic et al. 2012	United States of America	Initial Evaluation of a Student-Run Fruit and Vegetable Business in Urban High Schools	Qualitative	Evaluation sample size n=72, across 13 focus groups.<br>Age: Grade 11 or 12<br>No further demographics reported<br><br>Setting: three participating high schools in South Sacramento, a multi-ethnic urban area that was recently identified as a major food desert. Eight focus groups with 54 students at the charter school, three focus groups with 10 students at the traditional high school, and two focus groups with eight students at the continuation school. All three high schools are in low-income neighbourhoods in South Sacramento, with

	STUDY CHARACTERISTICS				
**No.**	**1st Author + year of publication**	**Country of origin**	**Study title**	**Study design**	**Socio-demographic characteristics reported (age, gender, socio-economic status, ethnicity)**
					student bodies that are predominantly African American and Latino. More than 50% of students at each school are eligible based on family income for free or reduced-price meals from the National School Lunch Program.
56	Spencer et al. 2019	Canada	Food in Focus: Youth Exploring Food in Schools Using Photovoice	Qualitative	Sample size n=7,<br>Age: mean 16yrs, Grade 10-12<br>Gender: female n=5, not disclosed n=2<br>Location: rural n=3, urban n=4,<br>Setting: participants were recruited through 2 community organizations in Nova Scotia, Canada, rather than directly through schools. One organization was in an urban centre and 1 was in a rural community. 4 schools under 2 regions of the province represented.
57	Tiedje et al. 2014	United States of America	A focus group study of healthy eating knowledge, practices, and barriers among adult and adolescent immigrants and refugees in the United States	Qualitative	Sample size n= 127 (73 adolescents), 16 focus groups were conducted, with 4 focus groups per community. Adults and adolescents participated in focus groups separately. For each community there was an adolescent male group, an adolescent female group.<br>Age: 11-65 yrs, (Adolescents: 11-18yrs mean age 14.5yrs, adults: 19-65yrs)<br><br>Setting: Four well-established immigrant and migrant communities in Southern Minnesota were involved: Somali, Mexican, Sudanese and Cambodian.<br><br>Ethnicity:<br>Somali adolescents: n=21, 43% female, ages 14.3±1.1<br>Sudanese adolescents: n=15, 53% female, ages 14.3±1.9<br>Mexican adolescents: n=22, 59% female, ages 14.8±1.7<br>Cambodian adolescents: n=15, 60% female, ages 14.6±1.5<br><br>Language most commonly spoken at home (% English): 0% Cambodian adolescents, 27% Mexican, 24% Somali, 33% Sudanese
58	Tsui et al. 2012	United States of America	Engaging Youth in Food Activism in New York City: Lessons Learned from a Youth Organization, Health Department, and University Partnership	mixed method	Sample size n= 373 across 17 cohorts in 3 years<br>Gender: 56% female<br>Age: 14-18yrs<br>ethnicity: data available from cycles 2 and 3 for 240 participants show that 51% were Black, 27% Latino, and 21% reported more than one race or ethnicity.

	STUDY CHARACTERISTICS				
**No.**	**1st Author + year of publication**	**Country of origin**	**Study title**	**Study design**	**Socio-demographic characteristics reported (age, gender, socio-economic status, ethnicity)**
59	Utter et al. 2010* related study<br><br>Utter et al. 2011	New Zealand	Design, Development, and Achievements of a Youth-Led Nutrition and Physical Activity Intervention in a Pacific Community in New Zealand<br><br>Evaluation of the Living 4 Life project: a youth-led, school-based obesity prevention study	quasi-experimental, with comparisons made by two cross-sectional samples within schools.	School students aged 9-13yrs<br><br>Conducted in six schools across South Auckland, NZ. Four schools received the intervention, and two did not (as a comparison). The four intervention schools were based in Mangere, where 60% of the population is from the Pacific Islands, and 40% is under 20 years of age.<br><br>3881 students agreed to participate in the study out of a total 5837 students on the school rolls (66% response rate). Final sample at baseline included 1634 students. At follow-up, 2515 students were asked to participate and 1612 agreed (66% response rate). Only 24 participants were included in both the baseline and follow up measurements.
60	Vecchiarelli et al. 2006	United States of America	Students' Perceptions of the Impact of Nutrition Policies on Dietary Behaviors	Mixed methods	sample n=399 students in 12th grade,<br>gender: 50.5% female, 49.5% male<br>ethnicity: Asian n=9.3%, African American n=6.2%, Hispanic n=33.8%, pacific islander n=2.3%, white n=31.7%, other 7.7%, multiracial n=9%
61	Watts et al. 2015	Canada	A qualitative study exploring how school and community environments shape the food choices of adolescents with overweight/obesity	Qualitative	sample n=22, Age: mean 14.3 (1.9) [11-17], gender: 77.3% female participants had completed an E-health lifestyle modification intervention
62	Wojcicki et al. 2006	United States of America	Healthier Choices and Increased Participation in a Middle School Lunch Program: Effects of Nutrition Policy Changes in San Francisco	Mixed methods	Sample size n= 859 students from Aptos Middle School (grades 6-8). Ethnicity: 21.2% (African American), 34.9% (Asian American), 24.3% (Latino).<br>Before the changes, students were informally polled on their food choices by teachers - total polled not reported<br><br>SFUSD post survey: n= 4448 students in 16 of the 17 middle schools and 12 of the 15 high schools.
63	Wright & Nault 2013	United States of America	Growing Youth Food Citizens	Qualitative	Sample size =9,<br>Ages 13-16yrs,<br>Socio-economic status: low income
64	Yoshida et al. 2011	United States of America	Engaging Youth in Improving Their Food and Physical Activity Environments	Qualitative	sample n=36, gender: female n=24, male n=12. Ethnicity: Latino n=18, Asian n=7, white n=7, African American n=4.<br>92% = high school students (3 were recent graduates)<br>n= 3 (middle school), n= 33 (high school) - Age not reported

	STUDY CHARACTERISTICS				
**No.**	**1st Author + year of publication**	**Country of origin**	**Study title**	**Study design**	**Socio-demographic characteristics reported (age, gender, socio-economic status, ethnicity)**
65	CO-CREATE Project<br>Baillergeau et al. 2024<br>Broeer et al. 2023<br>Budin-Ljosne et al. 2023<br>Klepp et al. 2023	Netherlands, Norway, Poland, Portugal and the United Kingdom	Democratising participatory health promotion: power and knowledge involved in engaging European adolescents in childhood obesity prevention<br><br>Recruiting and engaging adolescents in creating overweight and obesity prevention policies: The CO-CREATE project<br><br>Recruiting and engaging adolescents in creating overweight and obesity prevention policies: The CO-CREATE project<br><br>Ethical considerations in engaging young people in European obesity prevention research: The CO-CREATE experience	Qualitative/ Mixed methods	In total, 199 15–18-year-olds were involved in the 15 CO-CREATE Youth Alliances for Childhood Obesity Prevention located in the Netherlands, Norway, Poland, Portugal and the United Kingdom.<br>Sample size: aimed for approx. 15 adolescents per Alliance (2 alliances x 5 countries - Netherlands, Norway, Poland, Portugal, and the UK) = 150<br>Age: 16-18 yrs<br>Recruitment:<br>Alliance members were recruited in two local geographical areas per country (with a focus on reaching out to underrepresented youth). Recruited through local organizations e.g. Schools, scouts, and with peers<br>Total sample = approximately 300 in the development of policies to prevent adolescent obesity. Recruitment of youth alliances: Youth were recruited from schools, youth organizations, scouts, and a municipality in the Netherlands, Norway, Poland, Portugal, and the United Kingdom and had various socio-economic backgrounds.<br>Socio-economic status: "Approximately 10% of the<br>System mapping workshops - recruited through schools, had various socio-economic backgrounds.<br>youth participating in the Youth Alliances came from families of low socioeconomic status"<br>Gender: "experienced difficulty in achieving gender balance in the project as most young people participating in the Youth Alliances were girls, with only one Alliance having a majority of boys."<br>Dialogue forum: members from the youth alliances and new groups of adolescents of various socio-economic backgrounds also recruited through schools and youth organisations
66	Banik et et al. 2023<br><br>*CO-CREATE, separate study	Poland - unique photovoice study	What policies are there and what policies are missing? A Photovoice study of adolescents' perspectives on obesity-prevention policies in their local environment	Qualitative	Sample size n= 41<br>Age: 16–18yrs, mean age: 16.87; SD = 0.88<br>Gender: 90% female<br>Recruited from three communities in Poland

	STUDY CHARACTERISTICS				
**No.**	**1st Author + year of publication**	**Country of origin**	**Study title**	**Study design**	**Socio-demographic characteristics reported (age, gender, socio-economic status, ethnicity)**
67	Conway-Moore et al. 2023<br><br>*CO-CREATE, separate study	5 EU countries: Netherlands, Norway, Poland, Portugal, and the UK	Co-creating obesity prevention policies with youth: Policy ideas generated through the CO-CREATE project	Qualitative	Total sample n= 199 adolescents across 15 Youth Alliances from five European countries (the Netherlands, Norway, Poland, Portugal, and the United Kingdom) between 2019 and 2021<br>Age: 16-18 years<br><br>No other socio-demographic details reported
68	Herstad et al. 2023<br><br>Grewal et al. 2023<br><br>*CO-CREATE, separate study	5 EU countries: Netherlands, Norway, Poland, Portugal, and the UK	Adolescents' capacity to take action on obesity: A concurrent controlled before-and-after study of the European CO-CREATE project<br><br>Assessing adolescents' readiness for action and attitudes towards obesity prevention: instrument development and psychometric properties	Concurrent controlled before-and-after study<br><br>Process evaluation	Baseline sample n=439 (Alliance members n=159, Comparison n=280) who completed the baseline evaluation questionnaire.<br>Post initiative evaluation questionnaire n=194 included in the analysis (Alliance members n=62 and comparison group n=132).<br>Demographics of sub-sample:<br>Age mean (SD): Alliance members =17 (1.1), comparison = 16.8 (0.9)<br>Gender: Alliance - females n=48 (77%), males n=13 (21%), comparison females n=98 (74%), male n=31 (24%), prefer not say n=3 (2%)<br>Family affluence: Alliance- low n=12 (19%), medium n=32 (52%), high n=18 (29%),<br>comparison- low n=15 (12%), medium n=56 (42%), high n=58 (44%)<br>Country: Alliance - Netherlands n=21 (34%), Norway n=8 (13%), Poland n=19 (31%), Portugal n=12 (19%), UK n=2 (3%)<br>Comparison - Norway n=48 (36%), Poland n=78 (59%), Portugal n=6 (5%)<br>Setting: took place between September 2019 and October 2020. The control groups were recruited through school<br>Two supplementary analyses were also conducted to assess the robustness of their results. To increase the number of participants from the Alliances, they created a sample (n = 248) with all participants from the Alliances who had responded to the questionnaire at baseline and at least once more.<br>Grewal et al. 2023: A total of 328 Norwegian and Polish adolescents from the alliances and control groups participated in the baseline study.

	STUDY CHARACTERISTICS				
**No.**	**1st Author + year of publication**	**Country of origin**	**Study title**	**Study design**	**Socio-demographic characteristics reported (age, gender, socio-economic status, ethnicity)**
69	Nesrallah et al. 2023<br><br>Ulloa et al. 2023<br><br>*CO-CREATE, separate study	5 EU countries: Netherlands, Norway, Poland, Portugal, and the UK - interviews mainly held in Norway	Youth engagement in research and policy: The CO-CREATE framework to optimize power balance and mitigate risks of conflicts of interest<br><br>Designing a youth-led Dialogue Forum tool: The CO-CREATE experience	Qualitative<br><br>Qualitative	extracted from Nesrallah et al. 2023. Study duration: March 2019 to January 2020<br>No details on adolescents participating in youth consultations informing the COI framework reported<br>Ulloa et al. 2023:<br>Interviews included youth representees aged 14-18 years in Norway - total of youth/30 participants not reported. No further personal or demographic information was collected. Workshops were also held with 16 participants, half were aged 16-18years.
70	Savona et al. 2021<br><br>Savona et al. 2023<br><br>*CO-CREATE, separate study	5 EU countries: Netherlands, Norway, Poland, Portugal, and the UK<br><br>6 countries: Netherlands, Norway, Poland, Portugal, and the UK, South Africa	Identifying the views of adolescents in five European countries on the drivers of obesity using group model building<br><br>System mapping with adolescents: Using group model building to map the complexity of obesity	Qualitative	2022 paper:<br>Total sample n=257, aged 16-18years at 18 schools across 5 countries. "the focus is on generating the diagram as a group, so we did not take further demographic characteristics of the individual participants such as exact age or gender."<br>2023 paper:<br>Total sample = 319 Young people aged 16-18 years across 24 separate groups from six countries (the Netherlands, Norway, Poland, Portugal, the United Kingdom, and the Republic of South Africa)<br>**2023 paper references 2021 paper for the workshops but it does not contain South African participants

Table S4. Data extraction of included studies – Extent (n=70)

	EXTENT						
**No.**	**1st Author + year of publication**	**Food-EPI policy domain/s addressed**	**Policy, guideline or intervention details**	**Mode of Participation**	**Methods of participation**	**Research cycle involvement**	**Frameworks, models or theories of adolescent participation used**
1	Addis & Murphy 2019	Food provision	The 'Healthy Eating in Schools' policy was introduced in 2013 which outlines the nutritional requirements and standards of food and drinks obtained from school reduce impact of poverty on diet and improve nutrition by supplying free emails for students from low-income backgrounds. This study aimed to understand adolescent perspectives on the 'Appetite for life' guidelines introduced in Welsh secondary schools that set out minimum nutritional standards.<br><br>Students tended to opt out of free school food provision as the new menu items content and meal type were not popular. They preferred portable snack foods accessed elsewhere. For example, they favoured cold foods over hot meals.<br><br>There was a negative reaction from students as they didn't have a say in the new guideline changes in the food provided at schools and led to resistance where they often opted out and consumed food from home or accessed offsite. Students indicated a preference for fast food and unhealthy snacks	Consultative	Focus groups<br><br>Participatory techniques were used to facilitate discussion. Collective lifestyles approach framework was used to analyse transcripts to understand behaviour in context using three domains: (i) patterns of consumption; (ii) the construction and maintenance of identity; and (iii) power relationships.	Traditional study participant	Not reported.
2	Akom et al. 2016	Food retail	Youth mapped retail outlets in their community to 'ground-truth' i.e. verify the location and correct information of retail stores such as grocery stores, liquor stores, corner stores, gas stations as documented in the regulatory agency (e.g. Alameda County Public Health Department) databases, and add anything missing.<br><br>Ground-truthing revealed majority of retail food outlets were liquor stores and corner stores, rather than grocery stores. The top three non-tobacco or alcohol-related products available at these stores were chips, soda, candy/confection-items (i.e. Snickers, Skittles, Honeybuns), many of which had high sugar, fat, and salt content. This was similar across the 5 communities.<br><br>Youth recommendations to improve food environments through retail stores include increase fresh produce, and healthy staples e.g. bread, milk, at affordable prices e.g. through food stamps, improve food safety, restrict advertising and sales of tobacco products and alcohol. Suggestions to mitigate food insecurity include locating local farmers markets near schools, or other youth community locations (this recommendation was implemented with a farmers' market at Castlemont High School launched during the 2011–2012 school year), as well as identify and locate opportunities to	Adolescent-led	digital community mapping activities - Mapping 2 Mobilize collaborative (M2M) - Local Ground mapping platform and Streetwyze community engagement platform involved collecting written data on paper maps, written field notes, as well as pictures, and audio field-notes.<br>Youth driven community health survey. Interviews shared youth perspectives of their experience of the project.<br><br>"We added an emphasis on a participatory strategy of having youth identify their community concerns, and then plan and engage in social action to change underlying conditions contributing to food apartheid in their communities" (p.1299)<br><br>"Youth uprising (adults and youth) were influential in helping to develop the initial focus on food access and food insecurity." (p.1295)	Identification of topic<br>Design or development<br>Conduct<br>Analysis<br>Dissemination	YPAR 2.0 - adapted from YPAR, includes using location-based technology, new media, and digital organizing to engage youth in 'people powered place-making' to promote health and "enhance civic engagement, democratize data, expand educational opportunity, inform policy, and mobilize community assets". (p. 1288). Further, it allows "deeper multi-directional analysis of power dynamics between race, space, place, and waste." (p. 1288).<br>ground-truthing – an approach in which community members work with researchers to collect and verify 'public' data

	EXTENT						
**No.**	**1st Author + year of publication**	**Food-EPI policy domain/s addressed**	**Policy, guideline or intervention details**	**Mode of Participation**	**Methods of participation**	**Research cycle involvement**	**Frameworks, models or theories of adolescent participation used**
			provide pop-up farmers' market sites, urban farm sites, or future grocery-store locations.				
3	Altares et al. 2022	Food retail, Food provision	The Youth CAN (Change.Activity.Nutrition) project was developed to engage youth to address childhood obesity in their community. It aimed to 1) engage and empower adolescents to become agents of change for health within their community 2) encourage collaboration with stakeholders to improve healthy eating access in Globeville Elyria-Swansea (GES) and assist in planning and implementation of identified projects.<br><br>Youth captured photos highlighting several factors influencing unhealthy food access such as fast-food outlets near each other, limited healthy options at corner stores. Further, food available at school was a barrier to healthy eating e.g. pizza served at lunch. Enablers were also identified in their community such as: community garden and availability of fresh products at a small local marketplace and urban farm, and fruit available at schools. In phase 2: a partnership was formed with the urban farm and marketplace for community work. A photovoice project from the summer program identified some community members had to travel over a mile to find healthy food retail stores. Group ideas included: additional community gardens, mobile food pantry, increased healthy options at fast food outlets, grocery store closer, promotion of healthy eating at school.	Adolescent-led	Phase 1-2: Three art mediums: Photovoice, graffiti-style art, poetry to convey community issues that impact them. Phase 3: A World Cafe platform/ workshop allowed youth to engage in deep dialogue with adults sharing their projects findings. Phase 4: implementation of youth developed projects	Identification of topic<br>Design or development<br>conduct<br>Analyses<br>Dissemination	Youth Participatory Action Research (YPAR). "Youth acted as co-researchers throughout PAR activities, directly involved in identifying facilitators and barriers to healthy eating and physical activity."
4	Anselma et al. 2023<br><br>Anselma et al. 2020<br><br>Anselma et al. 2019<br><br>Anselma et al. 2019<br><br>Anselma et al 2018	Food provision	The Kids in Action project involved activities led by students to improve physical activity and dietary behaviours. One led to a water policy that children can only drink water at school which included the instalment of a water fountain (school 3).<br><br>Students were involved as co-researchers in the development, implementation, and evaluation of actions targeting health behaviours.	Adolescent-led	Involved as co-researchers in the development, implementation, and evaluation of actions targeting health behaviours.<br><br>Children not involved as partners in the process evaluation/ focus groups<br><br>Participatory needs assessment (2019)<br><br>Focus groups<br><br>"some parts of the Kids in Action study were conducted without participation of the child-researchers such as the development of the matrices." (2019)	identification of topic<br>Design or development<br>conduct<br>Analyses<br>Dissemination	2023 - YPAR - Level 6 of Hart's children's participation ladder<br>RE-AIM framework to evaluate the study on: empowerment, collaborations, reach, effectiveness, adoption, implementation, and maintenance -

	EXTENT						
No.	1st Author + year of publication	Food-EPI policy domain/s addressed	Policy, guideline or intervention details	Mode of Participation	Methods of participation	Research cycle involvement	Frameworks, models or theories of adolescent participation used
5	Asada et al. 2017	Food provision	This study examines policy implementation and impacts of the Healthy, Hunger-Free Kids Act of 2010 (HHFKA) policies.<br><br>Participants supported the HHFKA. They also had suggestions to improve its implementation. For e.g. improve quality and presentation of food, engage students in meal reform initiatives, communicate the importance and intentions of school meals.<br><br>Students perceived they and other students were eating more fruits and vegetables due to the new policy, but they wanted more options of fruit and vegetables, including salad bars.	Consultative	Focus groups (approx. 60 mins) and follow up semi structured interviews (30-60mins) via phone	traditional study participant	Not reported
6	Balvanz et al. 2016	Food provision, food price	Study raised community awareness of youth perceived factors contributing to childhood obesity and produced achievable solutions.<br><br>Youth partners found a lack of access to healthy food and lack of safe recreation as primary contributors to obesity within their community. Social support from friends was believed to help prevent obesity. In response to the findings, two projects were envisioned and implemented in the community, a walkability assessment and an intergenerational community garden.<br><br>Barriers reported: choosing unhealthy options at school, feeling powerless to ensure healthy food purchased at home, healthy food more expensive than junk food.	Collaborative	Photovoice, youth advocacy<br><br>"Youth were reliable partners, demonstrated by being present in research sessions and collaborating to disseminate the results" (p.301)	identification of topic<br>Design or development<br>conduct<br>Analyses<br>Dissemination	Community-based Participatory Research
7	Bosco et al. 2017	Food retail, food promotion , food provision	Study aimed to explore young people's engagement with their food environment including food retail. Adolescents documented their interaction with the food environment, including at home, school and community. For e.g. they documented food/drink advertisements on their way to/from school.	Collaborative	Global Positioning System–tagged photography, Photovoice interviews, and surveys<br><br>"They were encouraged to take photographs of any food that caught their attention or was meaningful to them, including food seen in advertising, media, and stores and food consumed by themselves and others" (p.1484)<br><br>"The students also learned to map their own food routines and created online story maps that they presented at the end of the academic year."	identification of topic<br>Conduct<br>Dissemination<br><br>+ traditional study participants in survey	Mobilities framework - not a youth engagement framework
8	Breckwich Váquez et al. 2007<br><br>Minkler 2009	Food retail, Food promotion, Food price food trade and investment	The Literacy for Environmental Justice (LEJ) worked with youth interns to assess food insecurity–related problems and resources in the community and developed a local campaign (the Good Neighbor Program) to reduce tobacco subsidiary food products and tobacco advertisements and replace them with healthier food alternatives at stores. The Good Neighbor Program, works with policy makers and local businesses to expand community accessibility to	Adolescent-led	Data extracted from Breckwich Váquez 2007: 2x Focus groups with youth, 4x semi structured interviews with community and health department partners, and 4x semi structured phone interviews with policy/decision makers.<br><br>The LEJ youth (paid interns) and project staff conducted a brief survey with 130 Bayview residents to assess their needs and	Identification of topic<br>Design or development<br>conduct<br>Analyses<br>Dissemination	Community based participatory approach. Community Action Model to achieve policy-related change (Hennessey-Lavery et al., 2005)

	EXTENT						
No.	1st Author + year of publication	Food-EPI policy domain/s addressed	Policy, guideline or intervention details	Mode of Participation	Methods of participation	Research cycle involvement	Frameworks, models or theories of adolescent participation used
			healthy food.<br><br>Youth led policy research to understand food security such as through mapping shelf space in local corner stores, and interviews. This led to a voluntary policy piloted in 1 corner store, where youth promoted the healthy products sold. They also conducted in-store press events, taste testing, and promotional giveaways of canvas shopping bags and fresh produce. Youth also worked with a local graphic artist to develop and implement an award-winning media campaign to raise awareness about the Good Neighbor Program.		preferences regarding local markets, health behaviours, and daily nutrition. They also explored what incentives might encourage more local shopping. Additionally, the youth mapped the shelf space in 11 central Bayview corner stores to analyze the allocation of space for fast foods, tobacco, liquor, meat, and fresh produce, using a color-coded diagramming method on Quadrille graph paper.<br><br>Good Neighbor Advisory Committee brought together LEJ staff and youth, community organizations and residents, city representatives, and other stakeholders to decide on incentives for the Good Neighbour Program.<br>They also interviewed store merchants.<br>Voluntary policy piloted in 1 corner store. "LEJ youth promoted products in the pilot store by conducting in-store press events, taste testing, and promotional giveaways of canvas shopping bags and fresh produce. Youth worked with a local graphic artist to develop and implement an award-winning media campaign to raise awareness about the Good Neighbor Program in the Bayview (Reed, 2004)." (p. 347)<br>"in-depth interviews with community and health department partners, two focus groups with youth, and four semi structured phone interviews with policy/decision makers. The interviews included questions about the formation and evolution of the partnership; research methods, roles, and findings; policy-related goals, steps, and activities undertaken by the partners; barriers, successes, and lessons learned; and perceived contributions of the partnership's research and policy actions to changes in programs, policies, and practices."<br><br>"TFP leveraged state tobacco funds and funded youth-involved organizations that identified issues of concern to their communities and implemented the five steps of the TFP research-to-action model, the Community Action Model (Hennessey-Lavery et al., 2005), to achieve policy-related change" (p.344-45)		
9	Browne et al. 2020	Food provision, food promotion and food retail	Explores food provided at schools and food choice by secondary school students, teachers and principals.<br><br>Students conducted their own focus groups with peers, and a researcher conducted focus groups with teachers and one-to-one interviews with	Adolescent-led	Students conducted their own focus groups with peers, and a researcher conducted focus groups with teachers and one-to-one interviews with principals.<br><br>"Peer researchers also listened to their respective recording and highlighted/ noted	Identification of topic<br>Design or development<br>Conduct<br>Analysis<br><br>Also included adolescents who	Not reported

	EXTENT						
**No.**	**1st Author + year of publication**	**Food-EPI policy domain/s addressed**	**Policy, guideline or intervention details**	**Mode of Participation**	**Methods of participation**	**Research cycle involvement**	**Frameworks, models or theories of adolescent participation used**
			principals. The discussion guide developed in with adolescents.<br><br>Focus groups revealed their were mixed feelings about the provision of unhealthy foods at school. However overall, students, teachers and principals expressed dissatisfaction with food provision within their respective schools and reported unhealthy dietary behaviours among the student body as the norm. Students believed environmental infrastructures, practices and policies needed to be addressed to improve behaviours.		important dialogue on a hard copy of the transcript, which was accounted for in the next stage"<br><br>discussion guide developed in collaboration with adolescents (supplementary files)<br><br>"An ecological model of health promotion research with student collaboration formed the theoretical framework" (p. 985)	were traditional study participants in focus groups	
10	Browne et al. 2023	Food composition, food labelling	This pilot study examined food and packaging waste and nutrition quality within the school food system. Students collected waste over 1 day and documented data on waste categories. Nutrition labels were photographed for analysis. Recycling bins were contaminated with food waste. Nutrition labels from 132 food packages were analysed, of which 115 items (87%) were low-nutrient, energy dense foods. Confectionary, energy bars and desserts and savoury snacks were the most common packaged food groups.<br><br>Organisational level solutions suggested by students included returning to reusable dinnerware, the introduce biodegradable packaging and a compost at school for food waste disposal.	Collaborative	Waste audit: Students collected waste over 1 day and documented data on waste categories. Nutrition labels were photographed for analysis. Students created a video and participated in a focus group led by researchers. Quantitative data were summarised using descriptive frequencies. A Nutrient Profile Model was applied to summarise nutrition quality. The WHO nutrient profile model was chosen as the tool to categorise foods and beverages<br><br>"An authentic participatory or citizen science research approach was integrated, where consumers involved in food choice and waste generation took an active role in data collection and reflecting on findings."	conduct<br>Dissemination<br><br>Focus groups: traditional study participants	YPAR
11	Callaghan et al. 2010	Food provision	The 2008 Bill 8, Ontario's Healthy Food for Healthy Schools Act, requires school boards to ensure that food and beverages sold in vending machines comply with the nutritional standards in regulations. The Healthy Vending Machine Pilot Project aimed to promote a healthier food environment in schools. School vending machines were supplied (from February to May 2007) with healthier choices and conventional vending products at a 50:50 ratio for each product category<br><br>"Healthier choice" sales ranged from 14% to 17%. In all schools, vending revenues declined from 0.7% to 66%. Price, value, and taste were barriers that led students to purchase healthy products rarely.<br><br>Preference was given to "real" healthy snacks, such as yogurt, fruit, and vegetables, available in schools.	Consultative	Students' preferences were considered when healthier products were selected for the vending machines. Taste-test panels were conducted at the four pilot schools to determine students' product preferences for the healthier choice options. Focus groups were conducted to obtain students' opinions about healthy eating and vending choices.	traditional study participants	Not reported
12	de Carvalho et al. 2021	food retail	Adolescents mapped main markets where they acquire food. Using online mapping survey, places for new infrastructure and establishments could be selected, such as the new school or the fresh food street market proposed in the sketch mapping activity.	Adolescent-led	Participatory Geographic Information Systems (PGIS).<br>"Participatory mapping is recognised as a bridge to include young people´s concerns and knowledge within the dominant scientific and adult knowledge" (p. 526)	Conduct<br>Analysis<br>Dissemination<br><br>Survey: traditional study participants	Not reported.

	EXTENT						
No.	1st Author + year of publication	Food-EPI policy domain/s addressed	Policy, guideline or intervention details	Mode of Participation	Methods of participation	Research cycle involvement	Frameworks, models or theories of adolescent participation used
			Participants marked streets with no access to electricity and water, and the main markets where they acquire food. Then, they mapped places where landslides and flooding had occurred in recent years, according to their knowledge and experience.<br><br>Five community journals were produced - one suggestion was to set up a website for people to find places that sell fresh food. This led to proposals that could be integrated into new policies for Guarulhos and for Novo Recreio. For example, closer fresh food markets, urban garden implementation, renewable electricity sources, and installing trash cans in the community to avoid waste disposal in inappropriate spots. Other suggestions including lowering the prices for healthy food, and an urban garden with affordable prices.		"Adolescents showed how proud they were of being part of a planning discussion about their community and managers enjoyed the experience of listening to them and discovering their proposals"<br><br>"Five community journals were produced (Figure 5) and several suggestions for the mapped issues were discussed... students also included their considerations regarding climate change and their impacts on the community" (p. 524)		
13	do Valle Santos et al. 2019	Food provision	Students developed a sustainability project of their choice- the cultivation of a vertical herb garden within a youth centre. It aimed to increase healthy food access. Researchers facilitated by giving the adolescents garden/herb kits and provided education. Youth chose to use recycled bottles to plant the herbs.	Adolescent-led	1st phase: the research team used didactic play-oriented interventions, using images related to sustainability, in order to foster adolescents' critical reflection on the meaning of sustainability.<br>2nd phase: watched the UN 'Smurfs for the SDG's' and completed a quiz. Then, youth considered how they could enhance their own community environment which led to the idea of the vertical herb garden.<br><br>Researchers facilitated by giving the adolescents garden/herb kits and provided education. Youth chose to use recycled bottles to plant the herbs.	Identification of topic<br>Design or development	not reported
14	Elliott 2017	Food promotion and food labelling	Study focuses on young peoples thoughts on restricting marketing of food and beverages to young people aged under 13yrs.	Consultative	Focus groups	traditional study participants	Not reported
15	Elliott et al. 2022	Food promotion	This study measures teens exposure to food marketing via a smartphone app "GrabFM!" (Grab Food Marketing!) from mainstream and digital media and the built environment over 7 days detailing food brands, product, platform and indicators such as persuasive techniques used.<br><br>collectively, participants reported 339 advertisements, 23 food categories, and 163 distinct brands; the top reported brands were for salty snacks and confectionary products.	Collaborative	GrabFM! is an app on participants phone that tracks participant demographics (age, self-identified gender, postal code) and enables teenagers to capture individual instances of food marketing (via photo or screenshot), and to identify the "indicators" or persuasive appeals they feel make that marketing "teen-targeted". These indicators include: animated characters, teenaged actors, celebrities, language, music, humour, special offers, visual style, and theme.<br><br>Focus groups were also held to provide feedback on the indicators and platforms prior to developing the app.<br><br>Focus groups were held at the end of the study to seek "specific feedback on the appropriateness of the indicators, on the	design or development (process evaluation from focus group informed larger scale study)<br><br>also participated as traditional study participants in focus groups	Not reported.

	EXTENT						
**No.**	**1st Author + year of publication**	**Food-EPI policy domain/s addressed**	**Policy, guideline or intervention details**	**Mode of Participation**	**Methods of participation**	**Research cycle involvement**	**Frameworks, models or theories of adolescent participation used**
					usability and desirable components of the GrabFM! app, and on food marketing more generally. "<br><br>"Teens also participated in follow up online focus group discussions (as part of a larger study), in which they provided specific feedback on the appropriateness of the indicators, on the usability and desirable components of the GrabFM! app, and on food marketing more generally. " This informed a larger scale study		
16	Elliott et al. 2023	Food promotion	Teenagers identified and tagged examples of teen-targeted food marketing in their physical and digital environments over a 7-day period using the "GrabFM!" mobile app.<br>Collectively, the teenagers captured 1825 advertisements for analysis, with each teen submitting an average of six advertisements over the 7 days.	Collaborative	Participants were instructed to capture only the food marketing messages that they felt were specifically targeted at teenagers.<br><br>"Participatory design that means teenagers, not researchers, are both capturing and tagging the examples of food marketing collected."	design or development<br>collate	Not reported.
17	Findholt et al. 2011	Food retail	Rural youth shared their perspectives of community assets and barriers that influenced children's physical activity and diets in their county. The project was facilitated by researchers. Photovoice methods revealed limited access to fresh and healthy foods in local stores.<br>Youth members presented their findings to various leadership groups, including school boards, parent-teacher associations, civic clubs, and county government	Adolescent-led	photovoice<br><br>"we wanted to ensure that those who would be affected by the research outcome— the rural youth—would have an opportunity to participate in the research process and share their concerns and ideas with policymakers and researchers"<br><br>"The students were asked, as a group, to reflect on the patterns or the themes that had emerged in the photographs and stories." (p.188)	Identification of topic<br>conduct<br>analysis<br>Dissemination	participatory action research - the authors "wanted to ensure that those who would be affected by the research outcome— the rural youth—would have an opportunity to participate in the research process and share their concerns and ideas with policymakers and researchers" (p. 187)
18	Fleming et al. 2023	Food retail	Participatory workshops were designed to capture qualitative data on adolescents' lived experiences and perspectives of their food systems, food sustainability, and food security, and how improvements can be made. Three key themes emerged, which included experiences of food, challenges to food systems, and strengthening food systems.	Consultative	Participatory workshops were designed to capture qualitative data on adolescents lived experiences and perspectives.<br><br>Nine workshops delivered online and nine delivered face-to-face, however the activities were the same.<br>This included: food words, food system mapping, concentric circles to show societal levels of influence on food sustainability, food vulnerabilities, postcard to the president, plenary discussion.<br>The UNICEF country officer in each country was the dialogue convenor<br><br>"listening to adolescents and leveraging their agency for solutions is fundamental to the co-creation of a sustainable food system transformation that enables dietary change in adolescents (UNICEF 2022)."	Identification of topic<br>conduct (gather data through participatory workshops)<br>Dissemination	Rights based approach<br><br>Victorian Health Promotion, Youth Engagement Evaluation Framework
19	Freire et al. 2017	Food labelling	The study evaluated how well Ecuadorian consumers understand and use the traffic light label on processed food packages, which	Consultative	Focus groups with consumers (including children and adolescents)	traditional study participants	Not reported

	EXTENT						
**No.**	**1st Author + year of publication**	**Food-EPI policy domain/s addressed**	**Policy, guideline or intervention details**	**Mode of Participation**	**Methods of participation**	**Research cycle involvement**	**Frameworks, models or theories of adolescent participation used**
			indicates levels of added fat, sugar, and salt. Adolescents emphasised the importance of easy-to-understand labels, but they expressed it do not necessarily change their behaviour because flavour and convenience. They also enjoyed more freedom/autonomy to buy snacks with money given by parents, often purchasing from street vendors, local shops, or school cafeterias.		Interviews with representatives of companies that manufacture, or market processed foods.		
20	Frerichs et al. 2012<br><br><br>Frerichs et al. 2015	food provision and labelling	Youth led a cafeteria food labelling and social marketing campaign to improve eating habits. Youth worked on an initiative to promote low-to-no cost changes to the foods available in the high school cafeteria.	Adolescent-led	students asked community partners (Live Well Omaha Kids (a city-wide coalition of health leaders) and the University of Nebraska Medical Center College of Public Health) to assist with a campaign to improve healthy eating in their school.<br><br>Students and health leaders worked together to develop the messaging and graphic design of colour-coded placards with traffic light emblems. The placards were incrementally displayed in the cafeteria service line over a 7-week period (e.g., only the most healthful items were labelled the first week, and all food items were labelled the final week).<br><br>Youth developed the labelling campaign after conducting environmental audits of their school, through which they discovered challenges to identify the cafeteria's healthiest food options.<br>the students posted pictures and stories on the SaludableOmaha Facebook page to promote healthy food choices among their peers.<br><br>Students leading the project distributed the anonymous survey to a convenience sample of 11th and 12th graders in their school. They selected and surveyed homeroom classes pre and post 7-week campaign.<br><br>Cafeteria staff tracked number of fruit and veg being sold at canteen	Design and development<br>Conduct<br>Dissemination	Youth advocacy approach included community mobilization, social cognitive theory and social network theory
21	Frerichs et al. 2018	Food promotion, food retail, food price	The study aimed to develop and pilot test a systems science curriculum to engage rural African American youth perspectives on childhood obesity and enhance their understanding of and support for obesity prevention solutions.	Collaborative	four workshop sessions with youth that engaged them in systems learning activities such as guided systems diagramming activities. Surveys pre-session and post-session assessed participants causal attributions of obesity and support for obesity prevention policies.<br><br>phase 1: "Youth were guided to produce visual diagrams that highlighted system structures related to obesity. Youth produced two types of systems diagrams: (a) graphs over time (Andersen & Richardson, 1997) and (b) causal loop diagrams (Sterman, 2000). "Youth were guided to create their diagrams	Conduct<br>Analysis<br>Dissemination<br><br>Phase 2: traditional study participants completing surveys assessing satisfaction and acceptability of the system diagramming activities	Community-based participatory action research

	EXTENT						
No.	1st Author + year of publication	Food-EPI policy domain/s addressed	Policy, guideline or intervention details	Mode of Participation	Methods of participation	Research cycle involvement	Frameworks, models or theories of adolescent participation used
					by critically considering the data evidence as well as their own perceptions."<br><br>"presented on proposed solutions including a health promotion summer camp led by adolescent youth and fielding a neighborhood petition to collect signatures of community members who would support healthier fast-food options in the community." (p. 429)<br>Youth produced two types of systems diagrams: (a) graphs over time and (b) causal loop diagrams to highlight system structures related to obesity.<br><br>7 youth were also interviewed postintervention with both high and low levels of participation.		
22	Gangrade et al. 2023	food retail	Study aimed to assess the feasibility of the adapted Youth Engagement and Action for Health! (YEAH!) Program to empower adolescents to advocate for the promotion of healthy snacks available in corner stores in New York City. The adapted YEAH! program had the following three phases: (i) lessons on advocacy, nutrition, healthy snacking and food environment; (ii) corner store assessment and (iii) corner store advocacy presentations. The adolescents conducted corner store assessments in groups using photovoice taking pictures of facilitators and barriers to healthy snacking. They presented their findings to three food retail/policy decision makers.	Adolescent-led	Youth advocacy - interviews conducted to inform the feasibility study to understand barriers to healthy snacking and informed the new YEAH! curriculum.<br><br>"The adapted YEAH! program had the following three phases: (i) lessons on advocacy, nutrition, healthy snacking and food environment; (ii) corner store assessment and (iii) corner store advocacy presentations."<br><br>"The adolescents conducted their corner store assessments in groups using photovoice taking pictures of facilitators and barriers to healthy snacking"<br>and discussed during class with the SHOWeD method<br><br>"all the groups presented their corner store advocacy presentations (10min) to three food retail/policy decision makers"<br><br>Focus groups to assess process evaluation	Design or development<br>Conduct<br>Dissemination<br><br>focus groups - traditional study participants	Not reported
23	Hackett et al. 2015	food retail	The study aimed to identify the community's environmental assets and barriers that influence childhood obesity. Youth identified the availability of processed food in corner markets and limited healthy food options, access to fresh, local produce, and community connection. Results of the Photovoice project were presented to local policy makers and community members for action	Adolescent-led	photovoice<br>"Nine local youth took photographs to visually identify the community's environmental assets and barriers."<br><br>"the authors and youth identified themes during the sorting process by grouping the most powerful photos that were selected during the sessions. The students made a final selection of 33 photos that they believed were the best examples of Roosevelt's assets and needs. All participants agreed with the final selection of photos and quotes illustrating the themes." (p. 251)<br><br>"Final results of the Photovoice project were	Identification of topic<br>conduct<br>Analysis<br>Dissemination	Community-based participatory research

	EXTENT						
**No.**	**1st Author + year of publication**	**Food-EPI policy domain/s addressed**	**Policy, guideline or intervention details**	**Mode of Participation**	**Methods of participation**	**Research cycle involvement**	**Frameworks, models or theories of adolescent participation used**
					presented to local policy makers and community members for action."		
24	Hermans et al. 2017	Food provision	This study aimed to get a better understanding of adolescents' food and health perceptions and their willingness to be involved in a specific school-based prevention program, i.e., the Dutch "Healthy School Canteen Program."	Consultative	Focus groups<br>surveys	traditional study participant	Not reported
25	Hinkle et al. 2018	food provision, food retail	The Food & Fitness (F&F) community partnerships, funded by the W.K. Kellogg Foundation (WKKF) between 2007 and 2016, were established to create community-determined approaches to increase access to locally grown food and healthy places for physical activity for children and families in communities with inequities across the United States. Youth conducted school food assessments with the aim to ensure that everyone in their community had access to affordable, healthy, locally grown food and safe places to be physically active. Youth worked on ways to offer healthy food choices in public and school settings. Youth implemented nutrition education projects at their schools and earned school credit for teaching elementary students. These youth-led clubs also promoted local farm-to-school products, participated in national F&F summits, and organized community events like fun runs, walkathons, bike races, and local food dinners through the FEEST program.<br><br>Youth Advisory Boards were active in most partnerships, with varying levels of decision-making authority within the partnerships. Some YABs acted as semiautonomous bodies, doing projects—for example, community assessments, Photovoice, FEEST dinners, and corner store improvement projects.	Adolescent-led	Four models of youth engagement used 1) work with institutional partners, 2) create youth advisory boards (e.g. worked on photovoice community assessments, FEEST dinners and corner store improvement projects), 3) partner with community based youth organisations, 4) host youth engagement summits and conferences<br><br>Interviews to gather youth perceptions on the lessons they had learned, their challenges and successes, and what kept them engaged and motivated in the project.<br><br>Annual project reports, meeting minutes, and partnership notes summarizing youth activities and involvement were also reviewed.<br><br>"Youth advisory boards were active in most partnerships (80%), with varying levels of decision-making authority within the partnerships... These boards gave youth their own space to talk and work on youth-only projects." (p.37s)<br>"In other partnerships, YAB representatives actually sat on the steering committee, having an equal voice with adult members."<br>In one project, youth conducted school food assessments;<br>Youth summits and conferences were held throughout the 9 years	Identification of topic<br>Design or development<br>Conduct<br>Dissemination<br><br>interviews: traditional study participants asking youth about their experience	Positive youth development (Alicea, Pardo, Conover, Gopalan, & McKay, 2012; Lind, 2008) and social justice youth development (Cammarota, 2011; Iwasaki, 2016; Ross, 2011) frameworks, which honour youth voice, take a bottom-up approach to working with youth, and emphasize social change.<br><br>Ladder of Authentic Youth Participation. Authentic Youth Engagement was adopted, "defined as an equitable partnership between young people and adults where young people feel that they are valued, respected, and heard and where there is mutuality in teaching and learning with each group seeing itself as a resource for the other and offering what it uniquely can provide (ACT for Youth, 2011)" (p. 5S)
26	James et al. 1996	Food provision	Study aimed examine adolescents' participation in school nutrition programs specifically the National School Lunch program (NSLP) and School Breakfast Program (SBP), that provides free or reduced priced meals at school. Adolescents shared their opinions about the meals served in these programs, and their suggestions for improving the meals.	Consultative	Focus groups	traditional study participant	Not reported

	EXTENT						
**No.**	**1st Author + year of publication**	**Food-EPI policy domain/s addressed**	**Policy, guideline or intervention details**	**Mode of Participation**	**Methods of participation**	**Research cycle involvement**	**Frameworks, models or theories of adolescent participation used**
27	Jáuregui et al. 2023	Food labelling, food promotion	The four-phase study aimed to design a communication strategy to promote the use of front-of-pack warning labelling and the selection of healthy foods with Mexican children and adolescents.	Collaborative	"The participation of children and adolescents in co-creation workshops allowed them to transition from being research subjects to active contributors in designing the communication strategy, providing key factors that could influence behavior change towards using front warning labels (EFA) and adopting healthier diets"	design or development	Not reported.
28	Johnson et al. 2017	Food retail	Study explores how youth in Baltimore City perceive their food environment—particularly what they classify as its supportive (healthful) and unsupportive (unhealthful) components—and to rally youth to identify and change aspects of their own food environments to promote health. Participants discussed how their photos related to food availability, diets, and overall health in their communities.	Collaborative	Photovoice, focus group discussions<br><br>"One major limitation of the study was that the topic of the Photovoice study was decided a priori" (p.403)	Collate<br>dissemination	Not reported.
29	Jones et al. 2012	Food provision	The Food for Life Partnership is an England wide food programme working to transform food culture in schools by reconnecting individuals and communities with food production through food-related citizenship education. It includes working with school meal caterers to use local, seasonal, organic and sustainable foods, as well as reforms to use freshly prepared foods and advertise ingredient origins. Program also involved, food education e.g. farm visits, cooking, gardening. Surveys assessed youth attitudes towards food, citizenship and sustainability issues within schools.	Consultative	Surveys assessing attitudes towards food, citizenship and sustainability issues, derived from the Food Standards Agency Low Income, Diet and Nutrition Survey [23], the Big Lottery Well-being questionnaire toolkit [24], or were developed specifically for the programme.<br><br>The student questionnaire was administered on two occasions to similar Year groups in the same schools. This means that the study is not tracking longitudinal change in individual student behaviour.<br><br>Students also had the chance to engage in less formal learning through participation in school food policy making, school food reforms and extra-curricular activities.	traditional study participant	Not reported
30	Kebbe et al. 2019	Food price, food provision, food promotion, food retail	The study explored Anglophone and Francophone Canadian adolescents' recommendations to inform policy and program decisions to address obesity.<br><br>Adolescents' recommendations included: improve accessibility and availability of 'healthy foods' by providing economic incentives e.g. lower costs of healthy food to increase motivation for healthy eating, reduce tax on healthy foods and food provision: e.g. provide healthy options in school cafeterias, limit deceptive practices in food marketing by changing the digital food environment e.g. ban tv commercials of unhealthy foods, impact of digital editing of fast food ads to make them look more appealing,	Consultative	Semi-structured interviews. completed a preliminary step consistent with principles of patient-oriented research [15] to engage adolescent patients (as partners) in the design and planning of our study (please see Kebbe et al. (2018) [16] for details).	design or development<br><br>survey:traditional study participant	patient-oriented research to engage adolescent patients (as partners) in the design and planning of our study

	EXTENT						
**No.**	**1st Author + year of publication**	**Food-EPI policy domain/s addressed**	**Policy, guideline or intervention details**	**Mode of Participation**	**Methods of participation**	**Research cycle involvement**	**Frameworks, models or theories of adolescent participation used**
			showcase healthy items compared to unhealthy foods in supermarket isles and near cash registers, removing public vending machines to limit access to SSB's				
31	Kim et al. 2019	food provision, food retail	The youth led a community health and food security assessment survey using a mobile application tool where they uncovered community concerns about the health of residents and healthfulness of food choices in schools, as well as a significant difference related to confidence in making healthy food choices between those who are and are not physically active. The Tribe applied the study findings with youth in alignment with cultural values and practices investing in developing community gardens, improving school food quality, and promoting native food practices that incorporate physical activity	Adolescent-led	Youth-Led Community Health and Food Assessment Survey with photos. Co-creation of all phases of research and equitable access to tools and information.<br>1. the youth determined their research question and selected their method.<br>2. youth-developed survey to understand community attitudes and choices about healthy food and active living.<br>3. the youth also took photos that illustrated the physical environment and context of the community in which the research was conducted.<br>4. youth guided the analysis by identifying which survey questions they were most interested in analyzing first and discussing which variables to consider for assessing associations. the university researchers analyzed the data<br>5. generation of recommendations, and formal presentation of results. the youth interpreted the results and disseminated them to tribal and project leadership and in public presentations.	Identification of topic<br>Design or development<br>conduct<br>Analyses<br>Dissemination	Community based participatory research
32	Kontak et al. 2022	Food provision	The study aimed to understand youth perspectives on Health Promoting Schools activities which was adopted across the province that includes 4 pillars: includes four inter-related pillars: (i) Social and Physical Environment; (ii) Teaching and Learning; (iii) Healthy School Policy and (iv) Partnerships and Services. Study also explored school youth engagement.	Collaborative	Peer led Interviews - Youth researchers were responsible for recruiting peers, collecting consent forms and administering the interviews.<br><br>"Peer researchers provided feedback that was incorporated into the final version of the guide"	Design or development<br>Conduct<br><br>Youth in interviews - traditional study participants	Youth Participatory Action Research<br><br>"Though the purpose of this study was not to specifically categorize responses based on Hart's Ladder of Participation (Hart, 2008) on youth engagement, the findings seem to align with level five of the ladder—consultation and informing. This observation is similar to Larsson et al." (p. 11)
33	Leung et al. 2017	food promotion, food retail, food pricing, food composition	The study aimed to understand youth perceptions related to food justice within their own community and identify solutions to promote positive change, using photovoice. The food justice program was 24 weeks long and comprised three phases. 1st phase involved photovoice. Last 2 phases included planning a youth-led community food justice project and taking action to implement it.	Adolescent-led	Photovoice, community food assessment conducted in groups of three to four. The youth took photos documenting barriers and facilitators of healthy eating in various venues, including street carts/vendors, fast food establishments, and bodegas (convenience stores).<br><br>semistructured interviews and a focus group discussion about selected photos.<br><br>youth participants also interviewed neighborhood residents on the streets to better understand how community members "experience" the food environment to help inform future strategy implementation<br><br>"food justice curriculum was developed through a collaborative process with program staff (Director of Nutrition and Foods	conduct<br><br><br>interviews & focus groups - traditional study participants	Community based participatory research

	EXTENT						
No.	1st Author + year of publication	Food-EPI policy domain/s addressed	Policy, guideline or intervention details	Mode of Participation	Methods of participation	Research cycle involvement	Frameworks, models or theories of adolescent participation used
					Programs, food justice coordinator, and instructors) and academic researchers and research assistants (RAs)."		
34	Lilo et al. 2021<br><br>Lilo et al. 2023	food promotion	The Choose H2O Challenge (Challenge) was an 8-week social marketing campaign on Facebook and at a high school. It was designed to encourage teens to think critically about beverage companies' marketing strategies, to educate teens regarding the health risks of SSBs and the benefits of water, to encourage behaviour change, and to encourage them to become health messengers to family and friends.<br><br>Prior to launch, the school received two water-bottle filling stations providing filtered, chilled water, replacing two standard fountains. The study explored students' perceptions of their habits, and the associated health risks and benefits surrounding water and sugar-sweetened beverage consumption to influence future messaging to change behaviour	Consultative	The researchers consulted regularly during design with a student advisory group, selected by school staff, and with teachers.<br><br>Focus groups informed the social marketing campaign with students, parents and teachers e.g. most participants believed soda was harmful but were unaware of harms from other SSBs (e.g., sports drinks, teas), advertising convinced parents and students sports drinks are necessary for student athletes<br><br>The challenge consisted of 2 parts: week school-wide contest, we invited students to complete weekly "Splash Tasks" on Facebook (e.g., water infusions, sugar calculations) earning points and having chances win prizes Phase 2: students worked in groups to create "QuenchCasts," focused on campaign goals. E.g. students developed promotional messages and strategies to supplement the campaign.<br><br>mixed-method evaluation approach: (1) semi-structured interviews conducted 2–4 months post-campaign; (2) structured observations counting water fountain/filling station use at three points, and (3) qualitative and quantitative analysis of social media engagement and QuenchCasts.	design and development (program)<br><br>interviews: traditional study participants (2021 & 2023 paper)	Not reported
35	Madrigal et al. 2014	food retail	Adolescents captured assets and strengths of their community related to environmental health. Key themes that emerged from their pictures and discussions were poor access to affordable, healthy foods; limited access to safe spaces for physical activity; ease of access to tobacco products; and poor working conditions for farmworkers. Adolescents encouraged stores to sell fresh fruits and vegetables to improve access to healthy foods. They presented their findings on radio, television, at local community events, and to key policy makers. The youth also developed two action plans, a successful 5K run/ walk and a school recycling project, still in progress.	Adolescent-led	Photovoice and questionnaire on self-efficacy. Youth were asked to take pictures of environmental health issues in his or her community, highlighting both problems and assets. The directions were intentionally kept to a minimum to allow the youth to select the themes and topics they wished to address through their photographs.<br><br>Common themes and issues were identified and discussed by the full group following each round of photo shoots using the SHOWeD technique.<br><br>"Participants presented their findings on radio, television, at local community events, and to key policy makers."<br><br>training sessions: "Youth also assisted in the planning and execution of sessions to their peers."<br>"The directions were intentionally kept to a	Identification of topic<br>conduct<br>analysis<br>dissemination<br><br>survey - traditional participants assessing changes in self-efficacy etc.	Community-based participatory research

	EXTENT						
**No.**	**1st Author + year of publication**	**Food-EPI policy domain/s addressed**	**Policy, guideline or intervention details**	**Mode of Participation**	**Methods of participation**	**Research cycle involvement**	**Frameworks, models or theories of adolescent participation used**
					minimum to allow the youth to select the themes and topics they wished to address through their photographs."<br><br>"The youth interpreted their own photographs and decided as a group where they wanted the photographs displayed."		
36	Mansfield et al. 2024	Food provision	This study examined student and caregiver preference for school communication and explored the feasibility and acceptability of a digital tool to assist with communication about school meal program between schools and families (The US National School Lunch Program (NSLP) and School Breakfast Program (SBP)). Three themes emerged around (1) communication preferences, (2) accessibility, and (3) extrinsic motivational factors for engagement in healthy eating behaviours.	Consultative	youth focus groups and caregiver semi-structured phone interviews.	traditional study participant	Not reported.
37	Martin et al. 2018	Food provision, food promotion	The study explored children and young people's views of health and what impacts their ability to live healthily, to help inform Ireland's National Obesity Policy. Participants recognised that eating behaviour was linked not only to individual food preferences but also to parental choice, peer influence, availability of healthy foods in school and in the community, the cost of healthy eating, and strong advertising of junk foods to children.	Consultative	A diverse range of methodological tools (e.g. 'lifelines', 'body maps', world café workshops) were used to collect data and optimise levels of participant engagement. Qualitative visual and written data produced during the consultations was then subjected to thematic analysis.<br><br>The consultation with young people (13–17) took place over one day and involved two main stages: lifelines followed by world café workshops. They discussed the following topics: Parents and Family; Bullying; Stress, Society and Peer Pressure; Magazines, Social Media and Photo Shopping; and finally Eating Disorders.	conduct	Not reported.
38	Sprague Martinez et al. 2020	food retail	A youth-led health assessment was conducted with researchers and local stakeholders. The youth identified health issues in their community such as mental and sexual health, food access, and community safety—and the recommendations they proposed for improving health and addressing inequities. The initiative was designed as a six-week workforce development summer program as part of an initial effort to engage youth voices in health care decision making. They also explore how youth engagement can be used to improve health care for young people, amplify young voices, and potentially shape future health system programming and metrics.<br><br>The initial study proposal aimed to be adult led but was revised to employ a more participatory approach centred on the strengths and knowledge of young people. Youth researchers developed a fifty-four-item survey protocol, conducted surveys, collected field work e.g.	Adolescent-led	The initial study proposal aimed to be adult led but was revised to employ a more participatory approach centered on the strengths and knowledge of young people.<br><br>"identified research topics to include on a survey about community health priorities, including access to mental and sexual health, food security, and community safety. Once these priorities were identified, youth researchers brainstormed a list of research questions that were revised iteratively and integrated in the survey protocol."<br><br>Youth researchers developed a fifty-four-item survey protocol. the youth researchers conducted ninety-three surveys in six Boston neighborhoods in which people of colour represent the majority of the population.<br><br>The youth researchers used Photovoice. They used cameras and their mobile phones to capture images within their neighborhoods	Identification of topic<br>Design or development<br>conduct<br>Analyses<br>Dissemination	Youth participatory frameworks e.g.<br>Wong NT, Zimmerman MA, Parker EA.A typology of youth participation and empowerment for child and adolescent health promotion. Am J Community Psychol. 2010;46(1-2): 100–14.<br><br>Cahill H, D advand B. Re-conceptualising youth participation: a framework to inform action. Child Youth Serv Rev. 2018;95:243–53.

	EXTENT						
**No.**	**1st Author + year of publication**	**Food-EPI policy domain/s addressed**	**Policy, guideline or intervention details**	**Mode of Participation**	**Methods of participation**	**Research cycle involvement**	**Frameworks, models or theories of adolescent participation used**
			photovoice that represented the health priorities they identified.		that represented the health priorities they identified. They selected and analyzed the photos using the SHOWeD framework.<br><br>Drawing on both the survey and Photovoice analysis, the youth researchers identified emerging themes of health priorities. They also developed health priority themes that integrated the multiple perspectives reflected in the data.		
39	Miller et al. 2021	Food price, food labels, food provision	Participants were asked their receptiveness to food policy options about tax on sugar sweetened beverages, nutrition warnings on sugar sweetened beverages, restricting sales at school and how it is promoted.	Consultative	Participants completed the 2018 National Secondary Students' Diet and Activity (NaSSDA) survey from May to December 2018	traditional study participant	Not reported
40	Millstein et al. 2016 a) &<br><br>Millstein et al. 2016 b)<br><br>Linton et al. 2014	food retail, food provision	The Youth Engagement and Action for Health (YEAH!) program (San Diego County, CA), run by the San Diego County Childhood Obesity Initiative (COI) engaged youth in advocacy for school and neighbourhood improvements to nutrition and physical activity environments.<br><br>Youth advocacy groups could select one or more assessments to perform and use the data to prioritise issues and create advocacy plans such as photovoice. They then compiled their findings into an advocacy presentation, identify relevant decision-makers, and present their results in person, in writing, or through other methods. Adult involvement includes training, leadership and funding support.	Adolescent-led	Advocacy groups select one or more assessments to perform and use the data to prioritize issues and create advocacy plans. Youth typically document neighborhood or school environmental problems using assessments or photovoice. They then compile their findings into an advocacy presentation, identify relevant decision-makers, and present their results in person, in writing, or through other methods. (Linton 2014)<br><br>Each group runs independently and led by adult group leaders who have discretion to determine the duration of the project and the frequency and agenda of meeting. Adult involvement includes training, leadership and funding support.	identification of topic<br>Design or development<br>conduct<br>Dissemination	Youth-engagement framework based on a framework for an evaluation of the Statewide Youth Movement Against Tobacco Use - The model recognizes multiple levels of influence leading to outcomes, including predisposing characteristics of participants, extent of participation, external support, and group climate, all taking place in various ecological contexts.
41	Mosavel et al. 2018	food retail	Conducted as asset mapping study to identify existing and potential assets to promote health including community resources to assist with healthy food options and promote physical activity. Youth-identified food asset maps mostly near their homes, schools, neighbourhoods, and the places they frequently visit such as grocery stores, vegetable gardens. They also found evidence of food insecurity.	Collaborative	GIS<br><br>The design of the asset mapping process allowed for a bidirectional, co-learning experience between Urban planning graduate students (outside experts) and youth (inside experts).<br><br>During community mapping walks, the team members would interview willing community members about a particular asset, or take photos and fieldnotes<br><br>analyses - Youth were centrally involved in identifying and categorizing assets.<br>"Friends and family came to hear the youth present the findings."<br><br>"Each youth and student completed an exit survey at the completion of the asset mapping that consisted of open- and close-	conduct<br>Analyses<br>Dissemination<br><br>Traditional study participants	Community-based participatory research

	EXTENT						
No.	1st Author + year of publication	Food-EPI policy domain/s addressed	Policy, guideline or intervention details	Mode of Participation	Methods of participation	Research cycle involvement	Frameworks, models or theories of adolescent participation used
					ended questions" e.g. reasons for participating, new skills learnt etc		
42	Muturi al. 2018	food retail and provision	This study examines the influence of youth empowerment on nutritional and physical activity factors associated with adolescence obesity.<br><br>Nutritional factors examined included efficacy for healthy food choice, efficacy for healthy eating and perceptions on healthy food availability. Their perceptions of healthy food environment were measured with six items examined their views on the availability of fruits and vegetables in their living environment (e.g. School, home, grocery stores)	Consultative	surveys (pencil and paper method)	traditional study participant	Community-based participatory research
43	Necheles et al. 2007	Food provision, food promotion	The study aimed for youth to identify influences on their health behaviours in a process that encourages the development of health advocacy projects. Students involved in developing plans for the project along with parents.<br><br>Photovoice allowed students with the opportunity to collect and interpret qualitative data on what they felt was influencing their health. Youth frequently discussed a variety of topics reflected in their pictures that included unhealthy food choices, inducers of stress, friends, emotions, environment, health, and positive aspects of family. The advocacy projects used social marketing strategies, focusing on unhealthy dietary practices and inducers of stress.G45:G46	Adolescent-led	Photovoice<br><br>The participants selected the issues they wanted to address and made a major contribution to understanding their data<br><br>"Our goal was to give youth the opportunity to express and explore their perspectives on what influences health in their communities"<br><br>Students involved in developing plans for the project along with parents<br><br>"This photovoice experience provided students with the opportunity to collect and interpret qualitative data on what they felt was influencing their health through the lens of a camera"<br><br>"The project was successful in voicing its findings to a larger audience through the exhibition, media coverage, community presentations, continued presence of the posters in participants' schools and community centers, and its influence on the center's pursuit of CBPR funding"	identification of topic<br>Design or development<br>conduct<br>Analyses<br>Dissemination	Community-Based Participatory Research
44	Ortega et al. 2015<br><br>Sharif 2015	food retail, food promotion	The project used a community-engaged approach to select, recruit, and convert four corner stores, so that they could become healthy community assets in order to improve residents' access to and awareness of fresh and affordable fruits and vegetables in their immediate neighbourhoods. High school students developed posters, flyers, and calendars that promoted healthy eating in the community as well as in the converted stores.<br><br>This study aimed to determine whether there were changes in community-level availability, purchasing, preparation and consumption of fresh fruits and vegetables, as well as to determine the profitability and sustainability of the corner store intervention.	Adolescent-led	In collaboration with local high schools, the YMCA, and an arts and civic engagement organization, we developed and implemented a variety of youth-led activities including outreach, health education and social marketing in the community. Students not only learned about community food issues and intervention strategies but they also developed the skills necessary to participate in all intervention activities, including designing the new store layouts, physically converting the stores, assisting in short- and long-term store maintenance, and developing and disseminating social marketing and health education materials. Students developed the project name, logo, and	Design or development<br>Conduct<br>Dissemination<br><br>Focus groups (Sharif et al. 2015): traditional study participant	Not reported

	EXTENT						
**No.**	**1st Author + year of publication**	**Food-EPI policy domain/s addressed**	**Policy, guideline or intervention details**	**Mode of Participation**	**Methods of participation**	**Research cycle involvement**	**Frameworks, models or theories of adolescent participation used**
			Sharif et al. 2015 aimed to understand young people's perceptions of their food environment and describe and examine the students 'experience with the corner store conversion to inform future campaigns to improve the community food environments.		promotional materials. They named the project Proyecto MercadoFRESCO (Fresh Market Project).<br><br>The social marketing and community activities included designing and distributing health education and promotional materials. For example, the students developed posters, flyers, and calendars that promoted healthy eating in the community as well as in the converted stores. These materials were prominently displayed in the stores and were widely distributed throughout the community.<br><br>Videos that were created by the students on healthy eating and that promoted the converted stores were played on city buses and shown at community events and schools. Students also went door-to-door distributing store flyers and coupons to incentivise patronage and garner support for the stores.<br><br>"In addition to leading the social marketing campaign, young people were actively involved in the stores' physical transformation." (Sharif et al. 2015)<br>Photovoice and focus groups (Sharif et al. 2015)		
45	Pawlowski et al. 2024	food retail and food provision	The study aimed to understand the perspective of Inuit youth aged 12–18 in Nunavik, northern Quebec. It focused on identifying the community conditions they believe support health and exploring their concept of a healthy community. Access to food was a key concern e.g. empty shelves in stores. Their dream community would be to have a bigger store that sold country food and food that was not expensive. School was considered an important place to provide food. They requested more fruit in breakfast club and country foods (fish, seal and berries) at lunch. Country foods were considered healthy as it allowed them to share food, which is part of their culture. They also requested a new affordable restaurant.	Collaborative	1 week participatory photovoice activity during regular class time.<br><br>Involved in analysis "Key words and phrases associated with each photo were written on sticky notes by the participants and facilitators and served as initial coding. The youth were given stickers to vote on the concepts most important to them" (p.S33)<br><br>Researcher conducted thematic analysis of transcripts of group discussions and fieldwork. 6 youth responded during validation check of data interpretation	conduct<br>analysis	Community-Based Participatory Research<br><br>Strength based approach
46	Jones et al. 2022	Food price, food promotion, food labelling	The study investigated how adolescents perceive the UK's Soft Drinks Industry Levy (SDIL), after it was implemented in 2016. This levy targets manufacturers, importers, and bottlers of sugar-sweetened beverages (SSBs) with two tiers: 18 pence per litre for drinks with 5-8 grams of sugar per 100 ml, and 24 pence per litre for drinks with more than 8 grams of sugar per 100 ml. Certain beverages are exempt, including those with over 75% milk, over 1.2% alcohol, alcohol substitutes, 100% fruit juice, and powdered drinks. Additionally, manufacturers who sell fewer than 1	Consultative	Focus groups	traditional study participant	Not reported

	EXTENT						
**No.**	**1st Author + year of publication**	**Food-EPI policy domain/s addressed**	**Policy, guideline or intervention details**	**Mode of Participation**	**Methods of participation**	**Research cycle involvement**	**Frameworks, models or theories of adolescent participation used**
			million litres of drinks annually are also exempt, provided their products are not otherwise excluded.				
47	Pettigrew et al. 2017	Food labelling	2058 Australian consumers (1558 adults and 500 children) assessed preferences between different front-of-pack labels (FoPLs) a daily intake FoPL, a traffic light FoPL, and the Health Star Rating FoPL	Consultative	Survey - Items included early in the survey required respondents to view mock packs of four different food products featuring various FoPLs and rate the products on multiple criteria including perceived healthiness, tastiness, and value	traditional study participant	Not reported
48	Poplin et al. 2017	food retail	The study aimed to engage youth in urban planning and map food availability near their schools. The youth identified multi-cultural food restaurants available, with less grocery stores available. They also had suggestions to improve the community garden to have co-benefits, including improving a sense of well-being.	Collaborative	Geographic information systems (GIS)-based participatory activities designed to engage youth in urban planning.<br><br>Authors aimed to foster their “community agency” but also, through their use of GIS technologies, enhance their potential to conduct and lead local change<br><br>The program focused on several key aspects: the youth’s own leadership skills, the use of technologies, and the school’s community garden that would form the center of the action projects<br><br>"The youth experimented with GIS, imagined the next chapter of the garden’s story, devised action projects, and conducted community partner meetings with other (adult) community leaders." (p. 190)	conduct<br>dissemination	Authors propose an engagement framework consisting of participatory technologies combined with action projects. It aims to illustrate the link between the use of novel technologies in the process of co-creation combined with the implementation of the ideas through action projects. The fundamental components of the framework are: online GIS, volunteered geographic information, agent-based modelling, serious games for civic engagement, action projects.
49	Riggsbee et al. 2018	Food retail	The study aimed to better understand the perceptions of adolescents’ food environments and related food behaviours using grounded visualization and story mapping. Focus group and Photovoice findings indicated the need for healthier convenience food items in all environments.	Collaborative	Surveys - aimed to "understand the dietary behaviors and food environments of the school overall."<br><br>Focus groups - "Participants were asked to elaborate on three food environments (school, community, and home), including facilitators and barriers to making desired food choices."<br><br>Photovoice -"were asked to take pictures of their community and home food environments during two different weeks using their cell phones; they were instructed to take pictures of anywhere they acquired food items, any foods they commonly eat, any meals, and depictions of the different types of food environments they encounter"<br><br>Story maps were developed using ArcGIS Online informed by each data collection phase	conduct<br><br>traditional study participants	Not reported
50	Robledo de Dios et al. 2023	food retail, food provision, promotion	The study aimed to identify and analyse the barriers and facilitators to healthy eating and physical activity. Further it aimed to understand youth perspectives in the participation of various obesity policies. Youth highlighted barriers and	Consultative	Ten discussion groups were held, with seven to eleven participants per group, and two ninety-minute work sessions per group. They were followed by two deliberative sessions.	traditional study participant	Not reported

	EXTENT						
**No.**	**1st Author + year of publication**	**Food-EPI policy domain/s addressed**	**Policy, guideline or intervention details**	**Mode of Participation**	**Methods of participation**	**Research cycle involvement**	**Frameworks, models or theories of adolescent participation used**
			enablers in their food environment to healthy eating.				
51	Rose et al. 2021	Food provision	Aimed to evaluate the impact of COVID- 19 school guidelines on secondary and post- 16 (16–18 years) food provision for pupils aged 11-18years	Consultative	An online survey was posted on 8th October to 1st December 2020, targeted at young people, parents and staff of secondary/post-16 education establishments in the UK	traditional study participant	Not reported
52	Santilli et al. 2011	food retail	CARE's 2009 initiative (Community Alliance for Research and Engagement at Yale University) aimed to improve chronic disease prevention by collecting and mapping environmental data, implementing targeted interventions, and engaging local youth as community health workers. They employed youths as community health workers to conduct asset mapping within their neighbourhoods and assess availability of healthy food in local stores.	Collaborative	They employed youths as community health workers to conduct asset mapping within their neighbourhoods<br><br>They found "engaging youths as community health workers is a successful model for collecting scientifically rigorous asset map data."<br><br>Three adult field captains mentored and oversaw safety of their teams of 2 to 3 youths.<br><br>Mapping involved collecting "geocoded coordinates and survey data on pharmacies; convenience, grocery, and liquor stores; fast food and sit-down restaurants; parks; gardens; and recreational facilities. Teams mapped the information environment (e.g., billboards) related to risk behaviors and documented marketing message"<br>They also did "a neighborhood street scan, rating each of 6 neighborhoods on 15 items regarding street safety, walkability, condition of streets and sidewalks, bike lanes and paths, adherence to traffic laws, and public transportation."	conduct<br>dissemination	Not reported
53	Shah et al. 2023<br><br>Foley et al. 2017<br><br>Shah et al. 2017	food provision	The Students As LifeStyle Activists (SALSA) programme provides an opportunity for students at schools to build leadership skills while motivating high school students to increase physical activity and improve their diet. The program was highly acceptable to students and had the potential to improve healthy food and physical activity environments at their schools. Youth peer leaders planned and devised actions, which included installing water refill stations, fitting the school gymnasium with new equipment, redesigning girls' sports shorts, and other strategies to engage girls in physical activity.	Adolescent-led	A key component of the SALSA programme requires students to identify and develop School Action Plans to improve access to physical activity and/or healthy eating in their schools.<br><br>SALSA Youth Voices (SYV), was developed in response to feedback from peer leaders in schools, who cited needing more time to develop and implement their School Action Plans<br><br>"The peer leaders considered many factors to suit the purpose of 'their School Action Plan' which was ultimately to install a water refill station. The factors they considered included accessibility for all students and staff, cost, placement, installation and filtered water. They did all the research for the water refill stations and decided on two options. The students sourced and corresponded with the suppliers to seek quotes. With these quotes,	identify topic<br>Conduct<br>Dissemination	Not reported

	EXTENT						
**No.**	**1st Author + year of publication**	**Food-EPI policy domain/s addressed**	**Policy, guideline or intervention details**	**Mode of Participation**	**Methods of participation**	**Research cycle involvement**	**Frameworks, models or theories of adolescent participation used**
					they organised a meeting with their principal and gained funding support."<br><br>"Students from all seven schools presented their actions to receive feedback and expressed confidence that their actions were sustainable." (Shah et al. 2023)		
54	Shaw et al. 2023	Food promotion, food labelling, food retail	The study aims to understand the interplay of social and food environments influencing adolescent food choice to help aid the development of interventions and policies. Some participants noted that nutrition information, such as calorie labelling, was difficult to interpret. Some thought if companies are willing to provide health claims on packaging, the item must be healthy. Participants described being aware of restrictions on the sales of energy drinks but were critical that these policies were not implemented in all store types. E.g. found loopholes where there aren't age limit restrictions (under 16yrs) imposed to buy energy drinks at convenience stores. Many spoke about using deals and promotions to get the most of their money.	Consultative	Focus groups. Data were collected between October 2020 and April 2021.	traditional study participant	Not reported
55	Sikic et al. 2012	food retail	This study evaluates the impact of Fresh Producers among students in the three high schools. Fresh Producers is a non-profit school-based program that provides high school students with an entrepreneurial experience and empowers them to become the source of convenient, affordable fresh fruits and vegetables in their communities. The program has the dual objectives of increasing fruit and vegetable consumption in communities with low access to fresh produce, while providing participants with experience running their own business. Students acted as co-owners and managed various business functions including distribution, product marketing, and point of purchase sales.	Adolescent-led	Evaluation - Focus groups.<br>"This study was designed as an initial evaluation of the acceptability, feasibility, and potential impact of Fresh Producers among students"<br><br>Student run fruit and vegetable distribution program<br><br>"Students function as co-owners would, managing various functions of distribution, product marketing, and point of purchase sales. They take weekly orders for fresh fruits and vegetables throughout the school year, and then receive seasonal, fresh produce through a wholesaler, which they repackage and arrange for customer pick-up" (p.1592)<br><br>Fresh Producers facilitated student activities in the community.	traditional study participants - focus groups, however program is adolescent led	Not reported
56	Spencer et al. 2019	Food provision	The study used photovoice to explore students' perspectives on school food. It aimed to let students capture and reflect on the strengths and issues related to their food environment, foster critical discussions, and present their experiences and concerns to decision makers through visual methods	Collaborative	Photovoice using the SHOWeD method, focus groups<br><br>Participatory process of analysis took place at each follow-up focus group through a process of selecting, contextualizing, and codifying. Participatory thematic analysis allowed participants sorted and grouped photographs and discussed themes and trends.	conduct<br>analysis	Study was informed by Wang et al. theoretical approach to photovoice i.e. participatory action research.

	EXTENT						
No.	1st Author + year of publication	Food-EPI policy domain/s addressed	Policy, guideline or intervention details	Mode of Participation	Methods of participation	Research cycle involvement	Frameworks, models or theories of adolescent participation used
57	Tiedje et al. 2014	food retail, food provision and food prices (briefly touches on food labels as a source of knowledge for healthy eating)	Aimed to explore the food preferences, beliefs and practices of dietary patterns, food environments and barriers to healthy eating of immigrant communities in America. Common barriers identified include easy access to junk food, cost. They also highlighted the importance of food labels to inform healthier eating choices.	Consultative	Focus groups	traditional study participant	Community-based Participatory Research
58	Tsui et al. 2012	Food provision, food retail, food pricing	Intervention aimed to increase the capacity of urban youth to take action to identify and reduce food-related health disparities. The intervention included 3 stages, hosted in partnership with local youth organisations:<br>1. Interactive workshops on neighbourhood health disparities<br>2. Youth-led food-focused research projects<br>3. Small-scale action projects designed to change the local food environment, including a school lunch campaign to advocate for healthier and more appealing food to be available in school canteens and a healthy value meal project where youth worked to develop a healthy version of a value meal (e.g. McDonalds Happy Meal) as an alternative offering to the fast-food alternatives readily available in the community. 10 youth-led action projects were completed, with most of them on a local scale.	Adolescent-led	Surveys, focus groups - study participant<br><br>Youth and staff developed local research projects. The youth participants defined a food-related problem, then carried out activities to document and analyze the scope and causes of the problem in thier community.<br><br>"9 of the 17 cohorts chose to assess food availability and preferences in the neighborhood food environments near their sites, 3 cohorts assessed both neighborhood food environments and school food, 2 assessed school food only, 1 cohort did not assess food, and 2 did not complete assessments" (p. 823<br><br>In the community-oriented research projects, youth identified a target area and criteria for assessing the quality, healthfulness, and price of selected food items. participants could choose the methods to answer research questions.<br><br>"Youth leadership in the design of research projects was largely determined by previously described partnership characteristics such as the amount and length of time trainers were able to spend working with youth and their attendance patterns. In longer cycles, trainers used a variety of strategies to move students through the curriculum efficiently while allowing them freedom to decide research topics and action projects for themselves."(p. 824)	Identification of topic<br>Design or development<br>conduct<br>Analyses<br>Dissemination<br><br>traditional study participant	Community-based Participatory Research
59	Utter et al. 2010* related study<br><br><br>Utter et al. 2011	food provision	The intervention (Living 4 Life) youth-led, school-based obesity prevention study that aimed to improve nutrition and increase physical activity among a population of ethnically diverse, predominantly Pacific Islander students. Specific areas addressed were sugary beverage consumption and breakfast habits, and improve the quality of foods sold at school.<br><br>Numerous activities were launched during the intervention, including breakfast clubs, Health Week, and rebranding of the school canteen. Water fountains were also installed in schools, and in one school there were improvements	Adolescent-led	Student health councils led initiatives within the schools to reduce obesity. "Full participation ensures that young people have control over the activities designed for them and are actively engaged... Early on, the student health councils created a vision and discussed roles and expectations of membership, objectives of the Living 4 Life project, and ideas for interventions/ actions. Once established, the student health councils created new activities and progressed and resolved issues with ongoing interventions. Students were responsible for setting the meeting times and agenda, securing	Identification of topic<br>design or development<br>conduct (responsible for delivery of intervention)<br><br>Utter 2011: traditional study participants via surveys.	Aligns with the principles reported in the Youth Development Aotearora Strategy, a government policy, which specifies that youth development happens when young people fully participate, when they are connected and have quality relationships, when the approach is strengths-based, and when it is shaped by the "big picture"

	EXTENT						
No.	1st Author + year of publication	Food-EPI policy domain/s addressed	Policy, guideline or intervention details	Mode of Participation	Methods of participation	Research cycle involvement	Frameworks, models or theories of adolescent participation used
			made to the canteen and eating area. Changes to canteen and eating areas include redesigning canteen menus to include healthier options, planting edible gardens on school grounds, and building a new covered outdoor seating area where students could sit to eat.		necessary resources to ensure productive meetings, and allocating responsibilities." (Utter et al. 2010 p. 1834)<br><br>Utter 2011: "Intervention activities were designed and implemented by Student Health Councils (SHCs) within each school." adult co-ordinator was employed to help facilitate implementation e.g. funding, supervision, and ensure capacity to achieve objectives.		
60	Vecchiarelli et al. 2006	Food provision	The study aimed to document the development, implementation and impact of the nutrition policies on student dietary behaviours at school. Over a half of students indicated that the policies positively impacted the foods and beverages they consumed at school whereas only about one fifth indicated the policies positively impacted what they ate or drank at home/outside of school.	Consultative	survey	traditional study participant	Not reported
61	Watts et al. 2015	Food Provision, promotion, price, retail	The study explored how school and community environments shape the food choices of adolescents with overweight or obesity through photovoice and interviews. Similar themes emerged from the school and community environments with food/beverage availability emerging most frequently, followed by peer influence, accessibility/convenience, price, classroom practices, marketing and online influences. Students described an obesity-promoting environment and perceived very limited healthful options	Consultative	Photovoice and interviews (phenomenological inquiry)<br><br>Each camera came with a card that instructed adolescents to take photographs showing what makes it easier or harder to make healthy food/drink choices at school and in other places they spend time in their community	conduct<br><br>traditional study participants	Not reported
62	Wojcicki et al. 2006	Food Provision	The study aimed to review the nutritional standards policy implemented in 2003 by the San Francisco Unified School District (SFUSD) The policy aimed to address problems associated with childhood overweight by eliminating the sale of unhealthy foods, snacks, and beverages from all lunch line, snack bar, and vending machine locations in all schools. Students were informally polled on their food choices. They suggested adding fresh deli sandwiches, pasta, and ethnic-specific foods such as sushi, and this was relayed to the SFUSDNutrition Committee. Student responses informed the new snack bar menus.	Consultative	Survey, data<br><br>Authors retrospectively compared school revenue and lunch participation data from the 2002–2003 school year (before implementation of the nutritional changes) with data from the 2003–2004 school year<br><br>a district-wide survey commissioned by the SFUSD Nutrition Committee to assess student eating practices. The survey was designed and administered by the SFUSD Research, Accountability and Planning Department.<br><br>"students were instrumental in the process of changing the menus in the snack bar at Aptos. Before the changes, students were informally polled on their food choices by teachers, and their input, which included the addition of fresh deli sandwiches, pasta, and ethnic-specific foods such as sushi, was relayed to the SFUSDNutrition Committee. Student responses formed the basis for the new snack bar menus"	traditional participants	Not reported
63	Wright & Nault 2013	Food retail, food price	The study aimed to help youth understand the complexity of the food system and develop the skills to assume rights and responsibilities of food	collaborative	Thematic GIS mapping. Youth also surveyed adults to identify the top 11 fresh produce items routinely purchased	conduct	participatory action research

	EXTENT						
No.	1st Author + year of publication	Food-EPI policy domain/s addressed	Policy, guideline or intervention details	Mode of Participation	Methods of participation	Research cycle involvement	Frameworks, models or theories of adolescent participation used
			to their community. They learnt about community food security, food environments, and food desserts and how to collect field data. The youth then collected data on the availability and cost of foods at local supermarkets and convenience stores.		"Youth were recruited as research assistants, stressing they would be collecting data to learn more about the Riverton food system"<br><br>"We instructed the youth on how to create maps using Google Maps and had them identify the retail food outlets in Riverton"		
64	Yoshida et al. 2011	food retail and food provision	This article describes youth's perceptions of their food and physical activity environments; involvement in community and policy change activities; and leadership development. Easy availability of unhealthy food (i.e., pizza and/or nachos) and candy and/or sodas in vending machines, short lunch periods, and poor role modelling by teachers in schools and in afterschool programs were cited as factors that discouraged healthy eating. Fast-food restaurants, gas station convenience stores, liquor stores, and food vendors and/or carts near their schools also discouraged healthy eating because of easy access to unhealthy food.	Consultative	focus groups<br><br>"Youth conducted community assessments, such as walkability audits, photovoice, and school mapping projects, which increased their awareness of the need for improvements in their communities:"<br><br>Youth were also involved in media work (e.g., created a documentary on fresh produce availability in the community) and taught nutrition to their peers."	Conduct<br>Dissemination<br><br>focus group - traditional study participant	Not reported
65	CO-CREATE Project<br>Baillergeau et al. 2024<br>Broeer et al. 2023<br>Budin-Ljosne et al. 2023<br>Klepp et al. 2023	food promotion, food retail, food pricing, food provision	The CO-CREATE project aimed to reduce the prevalence of overweight and obesity in adolescence. Youth Alliances allowed adolescents and adults to collaborate to develop policy ideas related to obesity prevention. When young people in the Youth Alliances were asked their interested area for policy, the most common themes included: mental health, self-esteem (both linked to social media), price and availability of healthy foods, access to gym and sport facilities, transport and time management.	Adolescent-led	Jan - May 2019: System mapping workshops: aimed to identify key drivers of obesity and generate policy ideas via group model building (Budin-Ljosne et al. 2023)<br><br>Summer 2018/ Summer 2020: Youth Alliances: activities involved developing and refining policy ideas using policy forms, photovoice, surveys and interviews. 10 meetings, each meeting lasting 1–3 hrs.<br>*Youth alliances are adolescent led but "the topic of focus was already decided (development of obesity prevention policies)... young people could decide on how often to meet, how to organize the alliances, and how to work with policy development, for instance deciding on the kind of activities to conduct, and which prevention strategies to adopt" (Budin-Ljosne et al. 2023 p.3)<br><br>June 2020/ March 2022: Dialogue forums - finalised policy ideas and discussion with stakeholders in dialogue forums using a dialogue tool to facilitate the discussions and could be moderated by young people Budin-Ljosne et al. 2023	Identification of topic<br>Design or development<br>conduct<br>Analyses<br>Dissemination	YPAR
66	Banik et al. 2023<br><br>*CO-CREATE, separate study	food retail, food promotion, food labelling, food pricing, food provision	A Photovoice exercise was designed to capture obesity-related public policies that were either present or absent in young people's local environments (with a focus on diet and physical activity)	Adolescent-led	Photovoice - photos were taken by the end of Feb 2020 (prior to COVID-19 pandemic lockdowns).<br><br>"During the fourth meeting, the adolescents participated in a group discussion where they presented two of their own photographs and discussed their content. They were encouraged to use photovoice reflections to	Identification of topic<br>Conduct<br>analysis<br>dissemination	YPAR<br>The photographs (n=213) were coded and mapped according to the policy themes they illustrated, using the MOVING and NOURISHING frameworks.

	EXTENT						
**No.**	**1st Author + year of publication**	**Food-EPI policy domain/s addressed**	**Policy, guideline or intervention details**	**Mode of Participation**	**Methods of participation**	**Research cycle involvement**	**Frameworks, models or theories of adolescent participation used**
					further discuss policy ideas, prioritize which policies they wanted to discuss with local stakeholders, and propose details for the content and implementation of those policies."		
67	Conway-Moore et al. 2023<br><br>*CO-CREATE, separate study	Food retail, food price, food provision, food promotion	The CO-CREATE project used a youth-led participatory action research approach to generate policy ideas towards the reduction of adolescent overweight and obesity across Europe. The authors analysed the youth-generated policy ideas to determine how many take a systems approach	Adolescent-led	Youth alliance meetings encouraged youth to (1) define the problem they sought to address and why it was important; (2) outline the main goal of their policy and its specific objectives; (3) identify the target group for the policy and relevant stakeholders; (4) provide a summary of their policy and the steps required to implement it; (5) develop a schedule for implementation; (6) prepare a detailed budget; and (7) conduct a risk assessment for their policy.<br><br>Policy ideas were then taken forward to a subsequent stage within the CO-CREATE project in which they were discussed in Dialogue Forums<br><br>"Alongside completion of the policy form, activities such as advocacy training, Photovoice and surveying were used by the Youth Alliances to understand the different contexts within which their policies would operate and the local needs involved"<br><br>Not included in analyses (researchers only for this paper) "Three researchers (KCM, CK, and HR) began by independently assigning each policy idea generated by the Youth Alliances to one of the five levels of the ILF (i.e., Paradigm, Goals, System Structure, Feedback and Delays, and Structural Elements)." (p.3)	identification of topic, Design or development, conduct	Youth Participatory Action Research - Intervention level framework to assess policy ideas. This framework "ascribes actions to one of five system levels, from Structural Elements, the least engaged with system change, up to Paradigm, which represents the system's deepest-held beliefs and is thus the most difficult level at which to intervene"
68	Herstad et al. 2023<br><br>Grewal et al. 2023<br><br>*CO-CREATE, separate study	Food retail, food promotion	Herstad et al. aimed to evaluate the effect on reported readiness for action and attitudes toward obesity prevention among adolescents who took part in the CO-CREATE Youth Alliances compared with a comparison group that acted as controls.<br><br>Grewal et al. outline how the process evaluation questionnaire was developed and to test inter-reliability of the baseline measurements.	Adolescent-led	Youth alliances - youth led policy. Alliance members contributed to adapt and implement these activities depending on the Alliances preference and need, thus making it more relevant to the local context.<br><br>Online questionnaire to measure readiness for action and attitudes toward obesity prevention<br><br>The questionnaire was translated, back trans-lasted, and pretested for time and comprehensiveness by adolescents from five countries - Grewal et al. 2023	identification of topic, Design or development Conduct<br><br>traditional study participants in evaluation survey	Youth Participatory Action Research

	EXTENT						
No.	1st Author + year of publication	Food-EPI policy domain/s addressed	Policy, guideline or intervention details	Mode of Participation	Methods of participation	Research cycle involvement	Frameworks, models or theories of adolescent participation used
69	Nesrallah et al. 2023<br><br>Ulloa et al. 2023<br><br>*CO-CREATE, separate study	Nesrallah et al. 2023 - N/A<br><br>Ulloa et al. 2023: food price, promotion	Nesrallah et al. 2023: This framework provides a road map to ensure that youth participation in the formulation of policies is safe and empowering. Nesrallah et al. 2023r presents a novel framework to mitigate conflict of interests in intergenerational policy dialogues, with a focus on power imbalance.<br><br>The dialogue forum tool was informed by the 23 insights, and developed in parallel to the COI framework, to integrate relevant COI measures into the design of the tool, materials, and five-step process<br><br>Ulloa et al. 2023 focuses on the development of a dialogue tool intended to tackle this issue, engaging, and eliciting insights on the theme of obesity prevention, by young people and for young people.<br><br>The dialogue forum was tested at the Stockholm food forum in Sweden June 2019 and was found to support meaningful youth engagement and provides guidance for stakeholders. Young people identified and elevated four obesity prevention policy domains to EU policy makers through a youth declaration 1) stop marketing of unhealthy food products to children under 18yrs, implement a sugar beverage tax "The aim of the Dialogue Forum tool was established before the start of the design process as part of the project's grant agreement and goal of engaging directly with young people to develop, make use of, and evaluate a model for multi-actor dialogue forums, bringing together adolescents, policymakers, and businesses to action commitments and policies to enable healthy nutrition and physical activity habits for obesity prevention" (p.3)	Adolescent-led	In spring 2019, a series of youth consultation and ideation activities were conducted in an effort to understand how youth and youth organizations engage in policy processes, multi-stakeholder dialogues, and civic engagement. The activities specifically aimed to address the following two questions: (1) what makes multi-stakeholder dialogues meaningful to youth and (2) what is typically not considered when multi-stakeholder dialogues with youth are designed.<br><br>"Principles have been adapted from best practices for meaningful youth engagement and policy co-creation in a multi-stakeholder dialogue setting, as well as youth insights from the ideation workshops."<br><br>Though the project was adult-initiated, youth were engaged in decision making and involved in the planning and implementation of various activities, including the Dialogue Forums.<br><br>Ulloa et al. 2023: 30 Interviews and two workshops with members of the CO-CREATE project, expert representatives, academic, business, political, non-governmental organizations, and youth representatives of youth organizations in Norway.<br><br>"Ethics approval was not sought for the interviews as they did not collect health or personal data and were not involved in health research, but rather consisted of consultations on youth involvement." (p.3)	Ulloa et al.:<br>Design or development<br>conduct<br>Analysis<br>Dissemination<br><br>"The aim of the Dialogue Forum tool was established before the start of the design process as part of the project's grant agreement and goal of engaging directly with young people - to develop, make use of, and evaluate a model for multi-actor dialogue forums, bringing together adolescents, policymakers, and businesses to action commitments and policies to enable healthy nutrition and physical activity habits for obesity prevention" (p.3)<br><br>"two additional evaluation workshops were organized with the project's Youth Task Force members, EAT staff, and other CO-CREATE consortium partner" to inform final prototype. not defined if youth task force members are young people	Youth Participatory Action Research
70	Savona et al. 2021<br><br>Savona et al. 2023<br><br>*CO-CREATE, separate study	Food promotion, Food price, food retail	As part of the CO-CREATE project, they conducted Group Model Building sessions with young people in six countries to create causal loop diagrams. These diagrams explored the drivers of adolescent obesity, as perceived by young people and situated them within a system. Adolescents mapped the commercial drivers of unhealthy diets including advertising of unhealthy foods, power of big food companies and accessibility of low-cost unhealthy foods.	Adolescent-led	Group model building workshops to create casual loop diagrams to represent young people's perceptions of the drivers of obesity.<br><br>"GMB provides a novel way of eliciting from young people the system-based drivers of obesity that are relevant to them"<br><br>used software called STICK-E (Systems Thinking In Community Knowledge Exchange)<br><br>20 maps generated by adolescents across 5 countries that were then merged by the research team<br><br>2023 paper: workshops were facilitated by staff. Group model building workshops. "Participants then work as a group to identify causal relationships between the variables on	conduct<br>Analysis (although adult researcher led)	systems thinking approach/ Youth Participatory Action Research

	EXTENT						
**No.**	**1st Author + year of publication**	**Food-EPI policy domain/s addressed**	**Policy, guideline or intervention details**	**Mode of Participation**	**Methods of participation**	**Research cycle involvement**	**Frameworks, models or theories of adolescent participation used**
					the circle, and these too are captured in STICKE (Figure 3) with the facilitator eliciting the direction (positive or negative) of the relationship between the two variables."<br>"participants were asked to spend some time coming up with action ideas"<br>Youth participated in the mapping workshops		

Table S5. Data extraction of included studies – Impact and Process (n=70)

		IMPACT		PROCESS			
**No.**	**1st Author + year of publication**	**Individual outcomes of adolescent participation**	**Policy related outcomes at a community, national or global**	**Barriers reported by adolescents**	**Barriers reported by adult researchers**	**Enablers reported by adolescents**	**Enablers reported by researchers**
1	Addis & Murphy 2019	Not reported	Not reported	Students in focus groups said they felt that their ability to influence school food provision was limited. A lack of information and consultation exacerbated the feeling that they were unable to influence these processes. Age was considered a barrier receiving information and the opportunity to participate in decision making.	The top-down approach gave students limited ability to negotiate food provision changes and was constrained by the institutional nature of school dining. Some reported they were not given information to make informed healthy decision and unhappy that "resented their choices being controlled" (p. 36). Diets in schools are influenced by peer norms.	Not reported	Not reported
2	Akom et al. 2016	The project led to an increase in young people's self-esteem, environmental stewardship, academic engagement, and positioned urban youth to become community leaders and community builders who are connected and committed to health and well-being of their neighborhoods and they felt inspired to take action.<br>Students had increased knowledge and awareness of social and environmental determinants of health.	The initiative sparked a 'food revolution' in East Oakland by raising awareness and influencing local action against 'food apartheid'. The success of a pilot store-mapping project garnered interest for expansion, with community and school partners noting that research and media efforts helped influence decision-makers. In Fall 2011, Castlemont High School launched a weekly farmers' market with produce from its student-run urban farm. In 2012, the Oakland Unified School District established a central Food Commissary in West Oakland, featuring a 44,000-square-foot kitchen, a 4,000-square-foot healthy food education center, and a 4-acre urban farm for students.	Not reported	Not reported	YPAR methods can help underserved communities, increase empowerment, skill development.	During the summer, participating youth were paid an honorarium for project participation and were asked to commit to summer work, college credit. Youth were trained to increase their understanding of food issues in East Oakland (e.g. food insecurity) as well as methodology, theories used in the Youth Uprising project via workshops. Authors stressed the importance of "starting where the youth are, participation, and the importance of creating environments in which individuals and youth become empowered as they increase their community understanding and problem-solving abilities" (p. 1299).<br>community-generated data was effective at improving accuracy of existing government official data. YPAR 2.0 model allowed for capacity building and responding to community needs
3	Altares et al. 2022	phase 1: built advocacy skills. internship opportunities with local agencies, including a summer youth leadership program, food education camp, and local redevelopment agency. Youth leadership development. engaging and empowering youth through PAR is fundamental in promoting positive youth development and enabling youth to become advocates for equitable food access in their communities. Phase 2: 8-week Summer leadership development program (n=30), facilitated youth leadership development and an increased awareness of community change, urban agriculture and nutrition. Entrepreneurship and workforce development is being facilitated through a local community family resource center.<br>- promoted self- efficacy, self-confidence, and advocacy skills	Project outcomes:<br>- A learning garden was installed in Spring 2021<br>- 'garden to cafeteria' program - produce grown by students at school sold in cafeteria<br>- school piloted an Introduction to Agriculture' course<br><br>Entrepreneurship and workforce development is being facilitated through a local community family resource centre.	Not reported	Not reported	Not reported	Mentorship was provided by the research team, and networking opportunities available through stakeholders. Weekly meetings were held with community stakeholders and research team which allowed them to "understand the challenges that youth face today and how to better connect with students to participate in Youth CAN". (p. 383).<br>Authors report that "taking the time to listen to youth, through student advisory board meetings and the World Café, promoted self- efficacy, self-confidence, and advocacy skills among youth" (p.390)

		IMPACT		PROCESS			
**No.**	**1st Author + year of publication**	**Individual outcomes of adolescent participation**	**Policy related outcomes at a community, national or global**	**Barriers reported by adolescents**	**Barriers reported by adult researchers**	**Enablers reported by adolescents**	**Enablers reported by researchers**
		among youth, increased their community health literacy. Evaluation methods not reported.					
4	Anselma et al. 2023<br><br>Anselma et al. 2020<br><br>Anselma et al. 2019<br><br>Anselma et al. 2019<br><br>Anselma et al 2018	Significant beneficial intervention effects were found on self-reported consumption of energy/sports drinks at T2 versus T0, and on total time and ≥5-minute bouts of moderate-to-vigorous physical activity at T1 versus T0.<br>Process evaluation: Empowerment improved in children who actively participated in the study. Developed critical awareness and thinking, self-confidence and research skills.<br>"At the end of the year, all groups felt that children were more involved in community decision making, especially through the activities they had organized themselves. Also non-Action Team members felt they had a voice in decision making and were satisfied with the participation of children in the community [AT2]."	process evaluation - the water policy was well supported at school because the idea came from the students. For e.g. a participant "was proud of the water policy that was successfully implemented at her school, despite children not being happy with this policy in the beginning. Also one of the boys who helped to implement the policy did not always adhere to it. But when the teachers told him *"Hey, this was your idea*!", he acknowledged that and without discussion threw his juice away."	Process evaluation: participants reported that they weren't taken seriously and participation in school decision making was low. For e.g. "They ask our opinion, but then they don't do what we asked. Then I just become angry." (2020)	"Due to the participatory approach the exact focus of the cocreated actions was unknown at the start, complicating the choice of optimal outcome measures" dynamic cohort, low participation rate e.g. "not all children who participated in the action development or in the implemented actions, participated in the measurements. Also, not all actions were accessible to all children, nor was participation in actions consistently registered. This creates the challenge of linking the participatory approach to the health behaviors outcomes." (2023, p.204).<br><br>"In Kids in Action, children mainly developed actions related to sports and play, and were less interested in developing actions to improve their dietary behavior. " (2023, p.207)<br><br>There were limited resources to enhance recruitment. Not all students that participated in activities were registered or recorded. Further, not all partners were engaged in all phases of the project and most actions focused on the school and neighborhood environment and less on the home environment and parents, therefore they suggest a systems approach. (2023)<br><br>Balance in sharing responsibility with children (2019)<br><br>"It was helpful to organize meetings during school hours, as children tend to be more focused. However, this does ask a lot of schools because the work is outside of the required curriculum" (2019)	The same researcher worked on the project over 4 years and allowed the children to get to know her and helped them stay involved. Students felt confident in their ability and could seek help from adult facilitator.<br>Community empowerment - "At the end of the year, all groups felt that children were more involved in community decision making, especially through the activities they had organized themselves." (2020)<br><br>Positive collaboration with researchers	Authors "recommend future studies to discuss with children their desired level of power sharing on each of the research topics, to make sure all topics are covered, and children participate on the level of their choosing" (2023, p. 207).<br>Process evaluation (2020): Action Teams were trained in research skills and developed, implemented, and evaluated actions.<br>Process evaluation (2020): Collaboration with school principal and community partners "ensured the continuation of child participation in the policy of community partners after the research project"<br>community networks<br><br>"It was helpful to organize meetings during school hours, as children tend to be more focused. However, this does ask a lot of schools because the work is outside of the required curriculum" (2019)
5	Asada et al. 2017	Not reported	"Students also advocated for policymakers, researchers, and child health advocates to increase opportunities for students to be involved in all stages of policy development, implementation, and efforts to sustain the practices that impact them the most. Also included in the Local Wellness Policy Final Rule is a requirement that students be included as a stakeholder that should participate in the	Not reported	"students were frustrated that their positive support for school meal reform was not accurately portrayed in popular media". Students also reported several ongoing issues, suggesting that the implementation of revised school meal standards has not yet been matched by strategies to improve quality and presentation of meals. Students prior to focus groups had "advocated for change	Not reported	Not reported

		IMPACT		PROCESS			
No.	1st Author + year of publication	Individual outcomes of adolescent participation	Policy related outcomes at a community, national or global	Barriers reported by adolescents	Barriers reported by adult researchers	Enablers reported by adolescents	Enablers reported by researchers
			‘’development, implementation, periodic review, and update of the local wellness policy.’’ (p. 848)		surrounding these issues prior to the implementation of school meal reform, including transitioning to scratch-cooking, improving cafeteria infrastructure, and increasing lunch period time" (p.848)		
6	Balvanz et al. 2016	Not reported	received a small grant to fund the community garden, "As of this year, seniors are still actively involved in the community garden, but current CBO youth are involved in other activities" (p. 300).	Not reported	Not reported	Not reported	youth received cameras<br>photovoice training sessions<br>graduate student partners facilitated a group discussion<br><br>walkability assessment: both youth and seniors dismissed preconceived notions they had of each other, remarking on their enjoyment of working with and learning the life perspective of each other.<br><br>"youth proved to be powerful research partners in identifying contextual determinants at multiple levels that were perceived to contribute to or deter obesity in their lives" (p.300)
7	Bosco et al. 2017	Not reported	Not reported	Not reported	"We were aware that young people’s participation in research projects is often a form of emotional labor that reflects uneven relations of power at multiple scales and potentially imposes burdens on them (Bosco and Joassart-Marcelli 2015). Thus, we did not ask for a specific number of photographs or demand a set level of effort"	Not reported	The qualitative data from photography, interviews, mapping, and surveys revealed that young people's perceptions of food and health are shaped by social relationships within their food environments. For instance, many young people viewed meals as healthy if they were home-cooked or shared with family, irrespective of the meal's actual nutritional value or ingredient quality.<br><br>Their participatory methods demonstrate the "capacities of young people as they negotiate their knowledge, consumption, acquisition of, and desires for food" (p.1495)
8	Breckwich Váquez et al. 2007<br><br>Minkler 2009	The partnership’s impact on the involved youth has been detailed elsewhere (Breckwich, Lanza, Hennessey-Lavery, & Minkler, 2005 - not published and unable to find online).	Data extracted from Breckwich Váquez 2007: Over 3 years, outcomes included: " youth-led research on the understudied problem of food insecurity, a successful pilot Good Neighbor store intervention, community outreach and education to influence community knowledge and behavior change, the development of a city- and foundation-sponsored initiative to expand the Good Neighbor Program, and state legislation to support similar efforts throughout California. "<br><br>By sharing their research findings with key local partners, the LEJ partnership was able to shape a local policy agenda focused on food security. They piloted a " voluntary policy targeted at corner stores and involving working with merchants to	Not reported	When youth were collecting field data "Merchants were often reluctant to speak with youth who, in the words of an LEJ staff person, “did not look any different than the youth who hang around in front of the store and are often associated with stealing or causing a nuisance." (Breckwich Váquez 2007: p. 345).	Not reported	Data extracted from Breckwich Váquez 2007:<br>LEJ youth were taught research skills e.g. conduct GIS mapping and produced maps highlighting locations of corner stores, supermarkets/grocery stores, transportation routes, and relevant demographic characteristics of the community.<br><br>"Community and TFP partners told us that the combination of research and media coverage both raised awareness of the issues and influenced policy makers to address it in this preliminary way." (p.347).<br><br>"A local policy maker commented that the partnership’s research findings and

		IMPACT		PROCESS			
No.	1st Author + year of publication	Individual outcomes of adolescent participation	Policy related outcomes at a community, national or global	Barriers reported by adolescents	Barriers reported by adult researchers	Enablers reported by adolescents	Enablers reported by researchers
			improve community food security rather than beginning with strong-arm regulatory strategies" (p.346).<br><br>Corner stores were to make several health-promoting changes in exchange for incentives, including:<br>food retail: Stocking at least 10% fresh produce, prioritizing organic and locally grown options, adding at least 10% healthy foods, excluding brands like Kraft or Nabisco,<br>Food price: offering products at affordable prices, articipating in food stamp and related programs,<br>Food promoiton: limiting tobacco and alcohol advertising and sales, including indoor height restrictions for displays and removing outdoor advertising. These changes and incentives were formalized through a memorandum of understanding developed with guidance from the Advisory Committee.<br><br>After pilot testing in 1 store, it "was seen as a local success with broad interest in replication and expansion." (p.347). Within the 7 months, fresh food sales had increased from 5% to 15%. At time of publication, 5 mores stores had signed up to the Good Neighbour Program<br><br>"Efforts are currently underway to evaluate the Good Neighbor Program, explore ways to expand it throughout the city, and develop state policy to support similar efforts throughout California"				LEJ's youth involvement "shed a lot of light" on the nature and urgency of the food security problem among city policy makers and decision makers." (p.348).<br><br>Youth were allowed to collect data in stores, which likely would have not been allowed if they were adults (Minkler 2009).
9	Browne et al. 2020	Not reported	Not reported	They identified commercial factors hindering school food policy action. One participant said, 'the school won't get rid of vending machines cause vending machines are the money makers, the school are stingy' is an example of how they as consumers interacted with food businesses within school' (p. 989)	Different priorities amongst teachers and principals to students e.g. education focused vs school food policy	Not reported	Provided unique insights of school food environments that differed to students and principles.<br><br>Students conducted their own focus groups without the presence of an adult, which represents a valued participatory method that can remove the power imbalances inherent in child/adult research and school scenarios<br><br>Ethics: Students who moderated focus groups attended training, and along with focus group participants, signed a confidentiality agreement. The researcher was available for advice at each stage of the peer-led research. Students and their parents signed informed assent and consent forms in advance of participation.

		IMPACT		PROCESS			
**No.**	**1st Author + year of publication**	**Individual outcomes of adolescent participation**	**Policy related outcomes at a community, national or global**	**Barriers reported by adolescents**	**Barriers reported by adult researchers**	**Enablers reported by adolescents**	**Enablers reported by researchers**
10	Browne et al. 2023	"Impacts for students included increased awareness and understanding of waste practices and clear judgements about their capabilities, influence and intentions for change." (p, 2319)	Adolescents demonstrated agency and critical thinking in their approach to solutions, identifying key actors needed for change at each level of the school community. This included addressing students' knowledge and behaviors, disseminating their findings among influential figures such as the student council, principal, parents, food service providers, and board of management, and improving environmental infrastructures to support change.<br>"Organisational level solutions suggested by students included returning to reusable dinnerware, the introduction of biodegradable packaging and a compost at school for food waste disposal" (p. 2317)	Not reported	Not reported	Not reported	Following recommendations for youth participatory research, participants were given space and time to reflect, communicate, and identify barriers, enablers, and solutions for a healthy and sustainable whole-school food system.
11	Callaghan et al. 2010	Not reported	Replacing 50% of vending stock with healthier snacks resulted in a decline in vending revenues. The cafeteria was identified as a better venue than vending machines for supporting and promoting healthy eating.	Not reported	Not reported	Not reported	Not reported
12	de Carvalho et al. 2021	Adolescents showed how proud they were of being part of a planning discussion about their community and managers enjoyed the experience of listening to them and discovering their proposals<br><br>"The process of participatory mapping associated with other participatory tools has made it possible for youth to become aware of the surrounding environment and problems that they face daily, stimulating dialogue and co-creation of knowledge and solutions and also reflecting their daily lives, since the map products were made by them." (p. 576)<br><br>the course helped build "empowerment, socioenvironmental awareness and even action towards overcoming routine problems, like mobility and waste"	material produced was disseminated to the community and to Guarulhos society through workshops, articles and other publications, such as blog posts on the project website (www.comunidadesvivas.com.br) and social media. A booklet was also developed, containing the research experience and materials developed, in a step by step process, with the main goal of dissemination and replication among other communities (free download): http://www.livrosabertos.sibi.usp.br/portaldelivrosUSP/catalog/book/429.	Not reported	Limited time was a reported barrier - "Due to restriction of time resources, it was not possible to teach the participants to digitalise the mapped information using the QuantumGIS software"	The process of mapping was reported as an enabler for adolescent participation as opposed to other participatory methods such as verbal expression of opinions: "mapping was a process that facilitated their engagement better than other participatory instruments that required opinions of expression by speech".	The course built capacity in youth, giving them skills to aid the mapping process and collaborative planning concepts in an easy, engaging and intuitive way.<br><br>Participatory maps can be used and have been used as an instrument to improve dialogue with city planners and other stakeholders
13	do Valle Santos et al. 2019	sense of empowerment by allowing them to consider “what they eat as it impacts their health”<br><br>participants also learnt how to plant and look after herbs for the first time, which ingited their curiosity<br><br>The participants felt a sense of pride	Not reported	Not reported	Not reported	Not reported	Not reported

		IMPACT		PROCESS			
**No.**	**1st Author + year of publication**	**Individual outcomes of adolescent participation**	**Policy related outcomes at a community, national or global**	**Barriers reported by adolescents**	**Barriers reported by adult researchers**	**Enablers reported by adolescents**	**Enablers reported by researchers**
		to learn that the herbs they grew were used in meals by the YC kitchen					
14	Elliott 2017	not reported	Not reported	not reported	"the teenagers accepted the food environment 'as is' (i.e. marketers lie, but there is nothing you can do about it) suggests that a more radical form of media literacy is necessary – one that also encourages teenagers to recognize that the current environment is not 'a given' and in fact can be changed." (p. 440)<br><br>"Given that the consumer information logic articulated by the teenagers is far removed from the public health logic of advocates of a ban, any successful public health initiatives need to consider how to bridge this gap." (p. 440)	Not reported	Focus groups were separated by gender and age to create an environment where participants are more likely to be at ease and to engage in the discussion
15	Elliott et al. 2022	Not reported	Provides interesting insights on how adolescents perceive what ads are targeted towards them. WHO is calling for greater monitoring of food marketing to young people	Not reported	Not reported	Not reported	Not reported
16	Elliott et al. 2023	Not reported	This research aims to support policy and monitoring initiatives in Canada	Not reported	Not reported	Not reported	Mobile app resonated with the teen participants
17	Findholt et al. 2011	leadership experience<br><br>One student co-presented the findings with two of the project facilitators at a professional conference.<br><br>Student evaluations revealed that participants gained significant benefits from the project, including increased understanding of their communities and factors affecting children's physical activity and food choices. Additionally, students developed research skills, public speaking, and confidence speaking to large audiences. One student decided to pursue a health career due to their involvement.	A newspaper article about the photovoice project findings led to significant exposure for the project, resulting in numerous invitations for the students and U.C. Fit Kids coalition members to present their findings to various leadership groups, including school boards, parent-teacher associations, civic clubs, and county government. Additionally, they were invited to present at a local college research symposium and a statewide meeting of the Oregon Commission on Children & Families. The project was also featured in several regional and statewide newsletters and websites.<br><br>The photovoice methodology was also then used in a project on highlighting the needs and experiences of homeless youth in Union County.	Some students indicated they wished for a longer study period to refine their photos	Not reported	"Students reported that they liked taking the pictures and sharing their stories with others, and enjoyed being part of an effort to improve the health of their communities." (p. 190)	participants were provided disposable cameras and paid an honorarium. Recruitment: youth interviewed by the Principal Investigator and their high school principal or teacher. The intent of the interview was to explain the project and participant responsibilities, and assess each student's ability and motivation to commit to the project. Parental consent and student assent were obtained following the interviews.<br><br>"The students clearly enjoyed their involvement in the project and felt a sense of ownership in the outcome" (p.189).<br><br>Methodology - provided a way to obtain input from local youth, thereby enhancing the quality and credibility of our research findings.<br><br>community members were highly interested in hearing from the youth. Thus, the method was very useful for increasing public awareness of the conditions in Union County that affect children's physical activity and diets.<br><br>authors "recommend a thorough project orientation that goes beyond a discussion of cameras and photographic ethics and

		IMPACT		PROCESS			
**No.**	**1st Author + year of publication**	**Individual outcomes of adolescent participation**	**Policy related outcomes at a community, national or global**	**Barriers reported by adolescents**	**Barriers reported by adult researchers**	**Enablers reported by adolescents**	**Enablers reported by researchers**
							helps the youth to understand how community context can affect health. The students in our study unanimously reported that the interactive discussion about how social changes have contributed to the obesity epidemic and the opportunity to view photographs taken by other youth as part of a community assessment— both of which occurred during the first photovoice session—were highly useful in preparing them for the project" (p.191)<br><br>Working with older youth is an important consideration when recruiting youth to photograph environmental influencers on health behaviours - previous studies had reported this as a barrier when working with younger children<br><br>Running a short term project over the summer meant that students did not have to attend school, meaning attrition was low
18	Fleming et al. 2023	Not reported	Presented the collective voice of young people at the UN Food Systems Summit 2021	Not reported	COVID-19 restrictions	Not reported	The Young and Resilient Research Centre team developed a comprehensive workshop manual to work with young people to explore their food systems. Authors recommendations to ensuring that adolescents' insights and perspectives are heard, acknowledged, and actioned in food system transformation:<br>- Leverage social media, including dedicated spaces on platforms for adolescents voices<br>- Establish and fund child-led bodies such as student councils and youth representatives<br>- Ensure there are people inside government and relevant organisations and institutions who are assigned to listen to adolescents and young people<br>- Use workshops, open forums, panels, conventions, meetings, drawing competitions, and other formats to capture adolescents needs, rights, ideas and aspirations<br>- Newspapers, journalism and televised public discourse (which involve adolescents)<br>- Promote opportunities for adolescents to share thoughts via letters, email campaigns, websites and respond to their demands<br>- Use research methods such as consultations, surveys, questionnaires

		IMPACT		PROCESS			
**No.**	**1st Author + year of publication**	**Individual outcomes of adolescent participation**	**Policy related outcomes at a community, national or global**	**Barriers reported by adolescents**	**Barriers reported by adult researchers**	**Enablers reported by adolescents**	**Enablers reported by researchers**
							and interviews to find out what adolescents think<br>- Visit schools to hear directly from adolescents
19	Freire et al. 2017	not reported	not reported	Not reported	Not reported	Not reported	Not reported
20	Frerichs et al. 2012<br><br>Frerichs et al. 2015	healthy eating - empirical evidence	Not reported	Not reported	"Youth in South Omaha were largely disconnected from community health leaders and were unfamiliar with the concept of leadership" (2012 paper)<br><br>"such projects also require more time and development to effectively address the disconnection" (2012 paper)	Not reported	Not reported
21	Frerichs et al. 2018	The youths' mean (SD) of the research and action participatory behaviour's scale significantly increased from 9.9 (3.8) at baseline to 14.5 (5.3) after the final session (p < .05; Table 2). Sociopolitical skills and motivation to influence, however, did not significantly change.	Not reported	Not reported	Not reported<br><br>"Some of the diagramming concepts appeared challenging for the youth"	Not reported	Increasing time for diagramming, the number of trained facilitators, or using a group format that facilitates youth to learn from each other should be considered for future implementation of the strategies.
22	Gangrade et al. 2023	Improved public speaking<br><br>Improved advocacy knowledge and efficacy - "I was always nervous about being an advocate and just like handing out stuff, you know, getting the word out, but now I know that I can do it different ways, and now I know that I can actually be an advocate'."	Not reported	the adolescents wanted more time and resources for the corner store improvement projects, such as more information about 'exact people or branches [of government]' that could support their improvement plans, as well as lectures from food advocates.	Not reported	In the focus groups / process evaluation, participants reported the sessions were fun	Received compensation for their time: $50 voucher for data collection as baseline and follow up and $360 stipend for participation in the program - may have increased retention and participation rate.<br><br>They ran the program over summer which may have also contributed to higher attendance and retention rates. Relationship-building fostered during the program and opportunities for youth to socialise and connect, especially since COVID-19 pandemic.
23	Hackett et al. 2015	Not reported	Final results of the Photovoice project were presented to local policy makers and community members for action.<br><br>"Two years after its completion, the academic institution, participating youth, and community-based organizations, found that the Photovoice project was a useful and effective research method that contributed to making positive changes in this underserved suburban community." (p. 255) - no further details provided	Not reported	Not reported	Not reported	An initial two-hour orientation session was held by the academic partner to inform participants about the Photovoice method and process, camera use and photography tips, as well as ethical considerations when taking photographs in the community.<br><br>The students used donated flip video cameras or their own cell phones to take photographs in the community. Since most students were able to use the cameras on their personal smart phones, everyday photo opportunities were more easily available.
24	Hermans et al. 2017	not reported	"it is recommended that greater efforts are made by schools on the implementation of their students' ideas"; by applying "a three step approach (1) survey student	Adolescents were not willing to participate themselves in the organisation of a healthy food environment in schools. This was mostly because they were sceptical	not reported	not reported	"Our advice would be to provide them with recognition and/or rewards to praise their time and effort. But most importantly: listen to them seriously and

		IMPACT		PROCESS			
**No.**	**1st Author + year of publication**	**Individual outcomes of adolescent participation**	**Policy related outcomes at a community, national or global**	**Barriers reported by adolescents**	**Barriers reported by adult researchers**	**Enablers reported by adolescents**	**Enablers reported by researchers**
			experiences and views at local school level, (2) involve young people in decision making by an action group comprised from students and staff, and (3) appoint an external facilitator or "critical friend" to ensure students voices are heard and to monitor progress (52). We recommend formalizing these steps in healthy food school interventions, such as The Healthy School Canteen Program" (p. 9)	about their ideas being heard and put into action by their schools			follow-up on their ideas in concrete and visible actions." (p. 9)
25	Hinkle et al. 2018	Examples of long term changes: (p.40s)<br>• Career identification through F&F Initiative experiences<br>• Personal lifestyles changed<br>• Youth are connected to regional programs with opportunities for growth through local food systems, built environments, and education<br>• Permanent, paid positions for youth (via community institutions, e.g., FoodCorps, health departments, tribal governments)<br>• Youth view themselves as change agents<br>• Youth presenters at national summits and meetings<br>• Prior youth members become elected officials on advisory boards<br>• Cultivated advocacy, leadership, and policy skills so that youth can be employed in food and policy change organizations	Examples include:<br>• Curricula changed at local schools to include food justice, food system issues<br>• Creation of culinary classes, expansion of school gardens, student-led evaluation activities<br>• School district food services has institutionalized youth advisory panels<br>• Neighbourhood plan for built environment now a regional model<br>• Youth now part of school wellness policy review<br>• School wellness teams formed with youth voice as team members	Not reported	Some partners experienced challenges with coordinating activities and transporting youth over long distances.<br>Communication differences between youth and adults required efforts to understand each other's language and adapt complex concepts.<br>Power dynamics also needed addressing, with adults needing to step back and allow youth leadership, while youth had to be encouraged to speak up.<br>Practical issues like meeting logistics and transportation were complicated by the availability of school-aged youth and adult preferences for meeting times.<br>The constant turnover of youth due to graduation and relocation necessitated ongoing recruitment and training of new participants.	youth reported that the key to keeping them engaged and motivated in the work was to be involved in "hands-on" projects; seeing, first-hand, the small changes underway in their communities; receiving stipends or incentives; and being allowed to take on leadership roles within their partnerships.	As part of the funding requirements from WKKF, F&F communities were required to actively involve community members in developing the vision and action plan. This approach ensured that communities with inequities, and the youth within them, felt a sense of ownership, had roles in the process, and were included in decision-making.<br><br>When adequately trained and mentored, these youth can fully participate in decision-making. e.g. pre-conference orientation session to educate common jargon, and practise networking skills with adults. they were also given responsibilities during the conference, so they felt they had a meaningful role.<br>Gender-balanced team of facilitators, with a good mix of young adults (e.g., 20- to 30-year-olds) closer in age to youth participants, with 1 or 2 older people as advisors.<br>Taking the time to train adults as well to be true allies to youth paid off in the level of quality of YE and input.<br><br>Working with established youth organizations was more effective than forming their own youth groups, due to the constant turnover of young people.<br>They realized the importance of a systems approach and sustainable structures to support youth engagement (YE). As a result, they decided to collaborate with a university cooperative extension system, like 4-H, to provide ongoing institutional support for their YE strategies.<br><br>For meaningful youth engagement to occur, "it is critical to have a framework to work from (e.g., Supplemental Appendix 1) and to think beyond project-based activities and instead envision a pathway for youth to acquire skills and

		IMPACT		PROCESS			
**No.**	**1st Author + year of publication**	**Individual outcomes of adolescent participation**	**Policy related outcomes at a community, national or global**	**Barriers reported by adolescents**	**Barriers reported by adult researchers**	**Enablers reported by adolescents**	**Enablers reported by researchers**
							experiences that can lead them to successful careers" (p. 44s)
26	James et al. 1996	Not reported	Not reported	Not reported	Not reported	Not reported	Not reported
27	Jáuregui et al. 2023	Not reported	Not reported	Not reported	Not reported	Not reported	Not reported
28	Johnson et al. 2017	not reported	Youth participants showcased their work at camp closing ceremonies and presented findings to Baltimore stakeholders, including local nonprofit and city officials. The project was also featured in the Global Obesity Prevention Center's newsletter, reaching over 500 subscribers.	not reported	not reported	not reported	Photovoice is an engaging and effective method to engage youth in efforts to improve food environments and diets.<br><br>Provided with a digital camera to use during the study and to keep.<br><br>4 sessions: icebreaker activity followed by a content component including short photography lessons and guided group discussions. At 2 of the sessions, participants chose 2 to 3 of their favourite photographs to discuss.<br>Used a storyboarding activity, to make the discussion more engaging for younger adolescents.<br>Strong collaboration between academic and community partners.
29	Jones et al. 2012	not reported	**Food leadership:**<br>School food policy and food action plan covering sustainability issues n=22 schools,<br>Student representation on school food action group or similar group n=22 schools,<br><br>Food quality and provenance of school food:<br>School menus are seasonal and highlight in-season produce n=19 schools,<br>Meat is farm assured and eggs are from cage-free hens n=14 schools,<br>Menu includes a range of locally sourced items n=14 schools,<br>Poultry, eggs and pork conform to Freedom Food scheme or 10% of ingredients are from a certified organic source n=10 schools	not reported	Organizational challenges of implementation in a school setting. Main challenge themes reported by staff:<br>Staff time, staff costs associated with developing management, development of school food policy and delivery of activities n=13 schools<br>FFLP criteria: meeting the criteria, paperwork n=11 schools<br>Caterer commitment n=9 schools	not reported	Main themes of successful aspects of the programme reported by staff:<br>Promotion of whole school food culture n=15 schools<br>Promotion of a healthy lifestyle / healthy eating n=14 schools<br>Development of cooking education n=10 schools<br>Promoting pupil participation and enthusiasm in school life n=8 schools need supportive school-level leadership.
30	Kebbe et al. 2019	Not reported	Not reported	Not reported	Not reported	Not reported	$25 (CDN) gift voucher for their time participating in interviews
31	Kim et al. 2019	Youth becoming more positive and confident about their ability to take action on their findings. One youth said, "I never thought that I would be trying that—to change the communities' health." Another youth mentioned, "I really like it. I feel like it's making you do more for the community."(p.84)<br><br>A third theme was increased awareness of their own health	"the youth identified more community gardens as a priority intervention. As a result, the tribe with community partners developed gardens at two elementary schools, a senior center, and the Native Plant Garden at the Karuk People's Center in Happy Camp, CA." (p. 87)<br><br>"The tribe secured a uSDA Farm to School grant to work with local school districts in order to increase the amount of fresh, healthy, locally produced foods served to	Not reported	Not reported	several participants mentioned that learning research skills required them to work past their initial discomfort to try something new. One person reported that the practice of approaching people made the interview experience easier each time. Another youth reported that the preference of taking pictures for photos	Youth Research and Leadership Training to develop research skills and offer leadership opportunities to apply those skills in the community - aligned with the CBPr-CS process: 1. Asking research questions and introduction of four common research methods (interview, survey, graffiti wall, photo-voice). the youth determined their research question and selected their method. 2. In-depth training on the chosen study design, survey with photos. the youth

		IMPACT		PROCESS			
**No.**	**1st Author + year of publication**	**Individual outcomes of adolescent participation**	**Policy related outcomes at a community, national or global**	**Barriers reported by adolescents**	**Barriers reported by adult researchers**	**Enablers reported by adolescents**	**Enablers reported by researchers**
		through the process of collecting community data. "It's taught me that I'm not that healthy, my family's not that healthy, but there is some aspects that are healthier than other people, but it's opened my eyes to see that everyone's not really that healthy even if they say they are" (p 85-6).<br><br>"The tribe convened workshops and demonstrations with youth and families to build and improve food production, processing, and storage skills, including canning, baking, butchering, building a chicken tractor, gathering and processing acorns, installing drip irrigation, and organizing seed swaps, and smoking locally caught salmon" (p. 87)	Klamath Basin children and to develop new culturally relevant "Native Health" lesson plans that emphasize the connections between traditional foods, physical health, and diet-related disease prevention for local K-12 students."			as a source of data collection was because ". . . pictures definitely a lot easier than the surveys cause like—you don't like actually to approach the people and ask them to take the survey. Like I kind of got nervous like asking people for surveys."	created and refined their survey instrument and prepared to conduct the research. 3. Overview of data analysis. the youth directed the analysis. 4. Detailed analysis, generation of recommendations, and formal presentation of results. the youth interpreted the results and disseminated them to tribal and project leadership and in public presentations.<br><br>the survey was implemented using a free iOS/ Android mobile data collection application (Magpi. com), and implemented on iPod touch. this application and device were selected to accommodate the lack of internet access and mobile phone service in the region.
32	Kontak et al. 2022	"When listened to, youth indicated that the feeling of empowerment and importance reinforced and inspired them to be part of engagement opportunities" (p. 10)	Not reported	"Fear of sharing their opinions and not knowing how to be involved made it harder for youth to be part of decision-making."<br><br>"youth indicated that there were not enough engagement opportunities within the school community. Not all students may be interested in being part of a club or would like to take a leadership position. It was indicated that differing levels and types of opportunities for engagement may aid in recruiting a diverse group of youth to be part of these initiatives."	Not reported	Key factors for youth engagement were: (i) safe and supportive spaces, (ii) passion and interest, (iii) using their voice, (iv) power dynamics, (v) accessibility and (vi) awareness.<br><br>Youth shared that teacher support and personal interest helped them be involved in decision-making. Further, it was vital to have a safe and supportive environment where they were comfortable to voice their opinions.<br><br>feeling like their voices were heard and listened to encouraged them to continue to take part in engagement initiatives;	2 days of in-person training in the summer of 202 for peer researchers.<br><br>Peer researchers received a stipend or their participation in the role to recognize their contribution at $13.50/h (CAD), which was slightly above the provincial minimum wage at the time of the interviews. Interviewees were provided a $15 (CAD) gift card for their time.
33	Leung et al. 2017	Not reported	Participants shared their photos with community members at a celebration/photo exhibit	Not reported	Not reported	Photovoice was an engaging and effective method to learn. "As noted by the instructors and youth themselves, it enhanced the overall food justice program and became an engaging process by allowing participants to be the researchers and advocates within their own communities." (p.217)	On completion of each home photovoice assignment, participants received a $5 store gift card.<br><br>Introductory sessions about photovoice methods<br><br>Participants were provided digital cameras

		IMPACT		PROCESS			
**No.**	**1st Author + year of publication**	**Individual outcomes of adolescent participation**	**Policy related outcomes at a community, national or global**	**Barriers reported by adolescents**	**Barriers reported by adult researchers**	**Enablers reported by adolescents**	**Enablers reported by researchers**
34	Lilo et al. 2021<br><br>Lilo et al. 2023	Lilo et al. 2023:<br>As the campaign progressed, EIBM constructs guided students to increase their feelings of self-efficacy and behavioural control to embrace changing norms about enacting targeted behaviours.<br><br>Students also demonstrated capacity to critically evaluate media influences, as predicted/influenced by normative beliefs based on media models.<br><br>Behaviour outcomes: "Most students reported quite drastic changes, doubling or tripling their water consumption, and significantly curtailing their SSB consumption, sustained at least three months post-Challenge; and supported by water bottle filling station observations data" (p.361)<br><br>Empowered to advocate - "Many students felt empowered to advocate for changes with friends and family because they had the knowledge and resources to feel like experts: they had tested the new behaviors themselves and were now modeling them for others." (p.361)<br><br>"Most people interviewed felt that increased knowledge, awareness, and the “coolness” factor of the campaign were catalysts for behavior change, suggesting that access alone is insufficient, but without a comparison intervention we cannot confirm" (p.362)	Not reported	Not reported	Not reported	Not reported	Not reported
35	Madrigal et al. 2014	perceived ability to make presentations, leadership, and self-confidence,<br><br>Youth were given the opportunity to present their findings to community leaders. creating conditions in which the youth could experience increased sense of empowerment.<br><br>Questionnaire assessed: Action self efficacy, sociopolotical skills, leadership efficacy, motivation and influence, participatory behaviour. significant improvements were seen on five questions capturing key	Participants presented their findings on radio, television, at local community events, and to key policy makers. The youth also developed two action plans, a successful 5K run/ walk and a school recycling project, still in progress.	Conflicting time schedules<br>Actioning their suggestions e.g. “[getting] to talk to people about what we are doing and our [action] project.” (p.325)<br><br>Transportation to meetings or to complete photovoice assignments<br><br>In general within their community, youth reported few opportunities for positive youth development. Further, stigmatizing media reports and attitudes about local youth, compounded these problems.<br><br>group dynamics e.g. "Having other people	"Training youth from a low resource community in the field of environmental health science presented several challenges"<br><br>Gaining access to people in power. E.g. "the slowness of change, particularly when government or other bureaucracies is involved" (p.327)	"combination of skill building and being able to make a difference in their community, while “having fun” in the process was a major strength leading them to recommend the program to others." (p.325)	Training sessions e.g. introductions to photovoice methods, research ethics and safety-related concerns, education on environmental health<br>Each participant was given a digital camera.<br>Each YCC member received an incentive of a $20 gift card at each session he or she attended, and snacks were served<br><br>“Research Ethics Jeopardy Game" helped youth understand research ethics.<br><br>Finally, the YCC youths’ identification of community strengths (e.g., helpful community-based organizations and

		IMPACT		PROCESS			
**No.**	**1st Author + year of publication**	**Individual outcomes of adolescent participation**	**Policy related outcomes at a community, national or global**	**Barriers reported by adolescents**	**Barriers reported by adult researchers**	**Enablers reported by adolescents**	**Enablers reported by researchers**
		domains of interest, such as perceived sociopolitical skills, ability to make effective presentations, and confidence in one's ability to work effectively with other students to try to help address a problem in their city.<br><br>"Significant changes over this period were noted on a few items in three of the five domains (action self-efficacy, sociopolitical skills, and participatory behavior) with no changes reported under motivation and influence or leadership efficacy" (p.323)<br><br>"“If I want to improve a problem in my city, I can work effectively with other students on this issue” (p = 0.01)." “I have spoken with adults in my school about issues that I want to improve in the school” (p = 0.03).<br><br>“a great opportunity for all of us to see how we can detect good and bad things and actually do something about it little by little. It's showed me how to speak to people about the problems we need to solve and how to actually make things happen.” (p.325)		talk and get out of the conversation” was distracting, while another similarly commented on the problem of sometimes “getting off track with a subject we were discussing.” (p.325)			adult role models) and their commitment to bringing their findings to the attention of civic leaders. YCC staff members maintained a strong and regular communication system and were flexible in scheduling sessions and other community events.
36	Mansfield et al. 2024	Not reported	Not reported	Not reported	Not reported	Not reported	Not reported
37	Martin et al. 2018	Not reported	The consultations with children and young people informed the Irish Obesity Policy and Action Plan 2016–2025, with specific actions to develop a whole school healthy lifestyles programme "(including, but not limited to, the curriculum, on nutrition, physical activity, smoking, alcohol and mental wellbeing), incorporating knowledge, skills and greater understanding of environmental factors that influence children and young people. National food standards for primary schools will be developed." (Department of Healklth 2016 p.37: https://www.gov.ie/en/publication/c778a9-a-healthy-weight-for-ireland-obesity-policy-and-action-plan-2016-202/)<br><br>The Action Plan also acknowledges the children and young people's complex and multifaceted constructions of health	Not reported	"Policymaking is one of the most challenging arenas in which children's participation rights are implemented and there is limited literature on children's participation in public-decision-making processes"	Not reported	A strict policy of confidentiality and anonymity was adhered to throughout the consultation process.
38	Sprague Martinez et al. 2020	Not reported	Youth researchers shared their research findings and recommendations at a culminating gala event at the end of their	Short time frame of the program.<br><br>Youth participants also identified limited	Although youth researchers were able to successfully conduct the youth-led health assessment, there was limited time to	Adults need to be ready to share decision-making power with them through listening	The team hired a young adult facilitator to assist with implementing several foundational training and curriculum

		IMPACT		PROCESS			
**No.**	**1st Author + year of publication**	**Individual outcomes of adolescent participation**	**Policy related outcomes at a community, national or global**	**Barriers reported by adolescents**	**Barriers reported by adult researchers**	**Enablers reported by adolescents**	**Enablers reported by researchers**
			six-week summer program, as well as during small-group meetings held at both the School of Social Work and Boston Medical Center's family medicine department.<br><br>"Since the project ended, family medicine physicians and social work researchers have focused on establishing a youth health advisory board as a strategy to involve young people in both decision making and developing health promotion materials."	resources, capacity, and adult support as challenges.<br><br>Community centre had limited internet access, and the support staff of the centre suffered from turnover during the course of the six-week program. In addition, the small number of youth researchers limited their ability to reach all six neighbourhoods (as initially proposed) with their health survey.<br><br>Youth noted the importance of authentic relationships with adults and wondered how institutional hierarchy might influence the creation of a meaningful youth board.	deepen relationships among youth and adults and engage in more comprehensive dissemination and action initiatives.<br><br>The initial framing of this study prioritized the importance of youth as experts, therefore diminishing the encouragement and support of adults. Although the leadership and growth of the youth researchers were significant over the course of the study, the youth identified the importance of continued adult engagement and support in future work.	and developing relationships, as well as by providing leadership opportunities for them. To achieve these goals, adults need to engage in ongoing reflection on power dynamics. (p. 1780)	sessions to support the project.<br><br>In the first week, youth researchers participated in an in-person training that were designed to help youth work toward three major goals: draw on lived experiences to identify health research priorities; build a strong understanding of racial health inequities, social determinants of health, system theory, and research methods; and strengthen social cohesion among youth researchers.
39	Miller et al. 2021	Not reported	This work complements national Australian data on adults, and demonstrates overall low opposition towards policy options to reduce consumption of SSBs among Australian adolescents which contributes to the political feasibility of such options. (ref: Miller C, Dono J, Wakefield M, et al. Are Australians ready for warning labels, marketing bans and sugary drink taxes? Two cross-sectional surveys measuring support for policy responses to sugar sweetened beverages. BMJ Open. 2019;9:e027962.).	Not reported	study shows a high degree of neutrality towards SSB policy options; "Adolescents may be somewhat less personally invested in health policy implications at this young age, and many may not have established views on such issues"	Not reported	framing policy options around educative and supportive measures may improve levels of support from adolescents.
40	Millstein et al. 2016 a) &<br><br>Millstein et al. 2016 b)<br><br>Linton et al. 2014	The program led to improvements in self-efficacy, advocacy ($p < .001$) participation ($p < .01$). Additionally, it led to improvements in knowledge and skills. For e.g. assertiveness ($p < .01$), health advocacy history ($p < .001$), resource knowledge ($p < .01$), social support for health behaviors ($p < .001$), and physical activity. Four factors—optimism for change, enjoyment of sports, roles and participation, and advocacy activities—were linked to advocacy readiness. Overall, the program enhanced youths' advocacy skills and meeting physical activity recommendations (Millstein 2016b)<br><br>Meeting physical activity recommendations increased from 3.62 (1.87) to 4.0 days/week (1.57), a 10.5 % increase from baseline ($p < .05$). (Millstein 2016b)<br><br>reported on improvements in youth's advocacy, knowledge of resources, and advocacy behaviour	Advocacy resulted in changes such as the addition of a salad bar at a high school.	Not reported	For some groups, the duration of the advocacy program was constrained by resources and time<br><br>"leaders often explained that their groups had completed assessments but were not clear about the next steps in engaging decision makers. More proactive technical assistance may be indicated so that leaders benefit from guidance about political decision-making structures (e.g., identifying who decides whether or not a school can get a joint-use field agreement for an adjoining city-owned property)."	Not reported	Gift cards were given to youth and adults who completed the baseline ($10) and post project survey ($15).<br><br>Technical assistance provided as needed<br><br>Evaluation led COI to enhance the YEAH! training by adding videos, more assessment tools, expanded resources, advocacy planning information, and case studies. Additionally, the project was scaled up to provide groups with an $800 stipend.<br><br>The YEAH! program's key elements were: strong partnerships with youth-serving organizations, dedicated adult leaders trained in policy and environmental change, and continuous expert support for advocacy. The COI initiative efficiently supported the program through its established community connections and experienced staff knowledgeable in local politics and obesity-related policies.

		IMPACT		PROCESS			
**No.**	**1st Author + year of publication**	**Individual outcomes of adolescent participation**	**Policy related outcomes at a community, national or global**	**Barriers reported by adolescents**	**Barriers reported by adult researchers**	**Enablers reported by adolescents**	**Enablers reported by researchers**
41	Mosavel et al. 2018	Increased communication skills related to talking to residents and professionals	The asset mapping data itself also serves as an ongoing resource for community partners. For instance, the data was shared with various local organizations to be used to inform their complementary efforts to enhance activity or health food choices.	Not reported	Not reported	Youth reported that they valued the help, skills, and ideas of the students (62%) as well as their personalities (46%).	the youth and college students jointly received three 3-hour training sessions at the community partner site facilitated by the lead researchers<br><br>Youth who completed the eligibility questions received a $5 gift card.<br><br>Youth received $400 for their participation, which lasted from June to the end of August.<br><br>Youth and students reported that the collective process enabled them to see assets and the community with new perspective.<br><br>Reported by uni students: Being able to find the balance between offering advice or encouragement and letting the youth discover on their own.<br><br>"Because they were considered research participants providing data, youth and students provided consent"
42	Muturi al. 2018	Not applicable	Not reported	Not reported	Not reported	Not reported	Not reported
43	Necheles et al. 2007	this work inspired the participants to continue voicing their concerns about youth obesity to the Center, which responded by successfully competing for an NIH CBPR grant to advance work on this issue.<br><br>several participants have continued to develop their community advocacy interest through center-organized advocacy-related skill building activities, involvement in community organizations, and college classes that address this topic.	Every group was concerned about obesity and encouraged the centre to do more research using participatory methods and led to "the center's success in partnering with the Los Angeles Unified School District on a new community-based participatory research (CBPR) project." and received funding.<br><br>One student took photos of unhealthy food sold in her school cafeteria and asked the school's Director of Food Services why there were no healthy options. She reported that her school cafeteria subsequently offered more nutritious food options for a 2-week period. The cafeteria added granola, yogurt, and whole fruits to its menu, and placed these foods physically in front of the less healthy options to encourage selection by the students	Not reported	Not reported	Not reported	Students were given digital cameras and received training.<br><br>The research team "developed relationships with the youth and their parents by participating in YAB, CAB, and community partner activities. This step was important for establishing trust between the research staff and participants, and developing the project structure and dissemination."<br><br>"This project supports anecdotal reports from previous studies about participants' enthusiasm for the photovoice experience. Photovoice can actively engage students in a process of identifying community-level health influences and creating advocacy approaches to promoting health."
44	Ortega et al. 2015<br><br>Sharif 2015	"developed the skills necessary to participate in all intervention activities, including designing the new store layouts, physically converting the stores, assisting in short- and long-term store maintenance, and developing and disseminating social marketing and health education materials. They also increased their media literacy by	Youth spoke to a variety of media, including local news, National Public Radio (NPR), British Broadcasting Corporation (BBC) radio, and CNN en Espan ˜ol.	Not reported	Not reported	Sharif et al: Identify young people who want to take charge and improve their community<br><br>Garner support from adults<br><br>"I like to be involved in projects and I like for people to make me feel like I am	"Having strong community involvement throughout all phases of the intervention strengthens community trust and awareness and allows for tailoring the intervention specific to community needs and contexts."<br><br>Working with an experienced professional in the corner store industry helps build trust with store owners and

		IMPACT		PROCESS			
No.	1st Author + year of publication	Individual outcomes of adolescent participation	Policy related outcomes at a community, national or global	Barriers reported by adolescents	Barriers reported by adult researchers	Enablers reported by adolescents	Enablers reported by researchers
		learning about the ways large food corporations market unhealthy and junk foods."<br><br>Increased knowledge e.g. learned about community food issues and intervention strategies<br><br>Youth spoke to a variety of media, including local news, National Public Radio (NPR), British Broadcasting Corporation (BBC) radio, and CNN en Espan ˜ol.<br><br>Sharif et al. 2015: built self esteem and self efficacy, leadership and public speaking skills, camaraderie, sparked interest in public health career, experience impacted personal and family dietary behaviour e.g. informing parents grocery purchases to be healthy<br><br>students were motivated to improve their own eating habits. The project implemented various strategies to maintain youth engagement after graduation, including internships and paid opportunities, which helped sustain and expand marketing and community nutrition education efforts.				important to the project and not just one of the workers." (Sharif et al 2015, p.410)<br><br>Focus on fun, educational opportunities outside the classroom	boosts their confidence in participating in the project both in the short and long term. Additionally, this expert facilitates communication between researchers and business owners.<br><br>"The youth were committed partners and brought a tremendous amount of enthusiasm to the work. Their involvement also generated much community interest and buy-in." (p.356)<br><br>"An elective course was implemented at two public high schools, one in East LA and one in Boyle Heights, to build the capacity of local students to lead the community social marketing campaign" (Sharif et al. 2015). They were also provided training on how to initiate behaviour change among their peers and relatives
45	Pawlowski et al. 2024	Not reported	Not reported	Not reported	Lengthy ethical approval processes combined with funding deadlines meant that relationship-building with the youth prior to arrival was difficult and made it unfeasible to engage one youth from each community as a research assistant as originally planned. Covid-19 pandemic limited planned research activities e.g. sharing their photos to the communities, following up with teachers. Fieldwork was limited to one week to reduce time spent away from school.<br><br>The researchers also planned for youth to validate thematic analysis but were restricted by COVID-19 travel and gathering restrictions and poor internet connection in this community. Instead, they gave a handout for teachers to share and make comments	Not reported	Photovoice was considered a fun and effective method of engaging youth<br><br>Five students did not return their parental consent forms. However, these students were not excluded from participating in the photovoice activity since it was conducted during class time (only excluded for the analysis)
46	Jones et al. 2022	Not reported	Participants were open to population-level interventions. These findings could help counter 'nanny-state' arguments against paternalistic government intervention on sweetened drinks, for example, that people	Not reported	The cognitive dissonance and othering (where participants discussed that 'people' should moderate their consumption of SSBs as opposed to 'I' or they should personally moderate	Not reported	Focus groups were conducted on school premises at a time and location most convenient for the participants and the hosting school.

		IMPACT		PROCESS			
**No.**	**1st Author + year of publication**	**Individual outcomes of adolescent participation**	**Policy related outcomes at a community, national or global**	**Barriers reported by adolescents**	**Barriers reported by adult researchers**	**Enablers reported by adolescents**	**Enablers reported by researchers**
			do not want unnecessary intrusion into their lives		consumption).<br><br>Covid-19 pandemic impacted running 4 more focus groups in 2020.		"Participants aged 11–14 were selected as the SDIL was included as a policy within the government policy: 'Childhood Obesity. A Plan for Action'"
47	Pettigrew et al. 2017	Not reported	"a stronger preference for the HSR across diverse subgroups indicates that it could be an effective population-level intervention of comparable utility to different categories of consumers" (p.120)<br>"Health Star Rating system that has been recently introduced in Australia and New Zealand appears to excel on two of these three criteria (ease of use and interpretive content), with the potential to also become more visually salient in the future if the nutrient icons are colour-coded" (p.121-22.)	Not reported	Not reported	Not reported	The inclusion of children in the study reflects their critical importance as both consumers and purchasers of food products, as well as powerful influencers on their parents' food purchase decisions.<br><br>Recruitment strategy: adults who registered for the survey who were known to have children in the target age range were contacted and asked to forward the survey link to their children if they were interested in participating<br><br>Panel members receive small financial incentives for participating in surveys<br><br>Questions in the survey were informed by focus groups and the instrument was initially soft-launched to assess respondents' (especially children's) ability to answer the questions. No adjustments were needed.
48	Poplin et al. 2017	reflections from program facilitator: "They had truly taken OWNERSHIP of this experience and were excited about the impact they could make. … I could see a difference from beginning to end with the group. Their mindset didn't just stop at this program either, I saw how overall they took their newfound role as a "LEADER" and used it throughout Club and school." (p.199)	10/12 (91% participants reported having shared their ideas with city or community leaders once or twice, a significant increase from 12% at the start.<br><br>A representative of the Community Growers group presented on the group's leadership and partnership at the City of Des Moines's Council meeting.<br><br>"This project has had substantial impact in three key areas: 1) gathering this community around the Hiatt Garden, 2) raising local leaders' recognition of the youth as co-creators in healthy, sustainable city-making, 3) empowering the youth as future leaders through technology."	Not reported	addiction - "increased use of the mobile devices and the applications installed on these devices."(p.200)<br><br>usability "GIS applications that are difficult to use or need a substantial time invested into learning may not be well adopted by the youth." (p.200)<br><br>"the youth had difficulties using the paper map and getting oriented with the help of this map... the use of the digital interactive online map served better for the main spatial orientation on the map." (p.201)	Not reported	"Our work seeks not only to integrate the voices of youth into city and neighborhood decision-making but also to empower these participants as community leaders who are learning to use technologies as collaborative tools that can help them work with community partners and take action." (p. 184)<br><br>Incorporated the use of technologies through a lens of storytelling<br><br>Established a relationship with staff at the Baker chapter of the Boys & Girls Club prior to project. Aligned interests e.g. focus on community leadership<br><br>"Novel devices and accessibility. GIS are increasingly available on a variety of devices such as smart phones, tablets"(p.200)<br><br>"communication & connection - These online maps enable novel ways of communication, sharing stories with the help of online maps and can even lead to empathy. They can be combined with pictures and can enable the youth to communicate in a novel way. They also enable them to express their

		IMPACT		PROCESS			
No.	1st Author + year of publication	Individual outcomes of adolescent participation	Policy related outcomes at a community, national or global	Barriers reported by adolescents	Barriers reported by adult researchers	Enablers reported by adolescents	Enablers reported by researchers
							environmental and neighborhood concerns and communicate with other neighborhood leaders and activists."(p.200)
49	Riggsbee et al. 2018	Not reported	Not reported	Not reported	They were unable to interview participants after completing the photovoice activity. Therefore, the research team allowed participants to provide comments when submitting pictures and following the creation of the story map.	Not reported	The survey was administered via an online platform and offered during class times and lunch periods.<br><br>Instructions, a guide for ethical photography, and a written prompt were provided in the classroom<br><br>GIS is used in "community settings to allow stakeholders and community members to better understand their shared experiences, story maps embed photos, videos, comments, and other information in an online, interactive map. Story maps provide context and socially constructed information beyond objective assessments"<br><br>"Use of grounded visualization and story mapping may be useful tools when evaluating adolescent food environments and related food behaviors." (p.11)
50	Robledo de Dios et al. 2023	"During the deliberative sessions, the participants show high leadership and initiative capacities, as well as a very participatory attitude with a tendency to listen and reach consensus, generating a fluid dynamic of teamwork and spaces for collective reflection."	Not reported	Not reported	Gender differences e.g. more girls participated, but boys were more likely to speak up.<br><br>Shyness in focus groups	Not reported	Discussion sessions took place during school hours (90 mins), deliberative sessions held after school hours.<br><br>Humour and jokes used to facilitate discussions
51	Rose et al. 2021	Not reported	The national survey data indicates that many secondary schools were not fully meeting the School Food Standards before the COVID-19 school closures in March 2020, despite the requirement for compliance. Specifically, the data suggest that numerous schools were failing to provide the recommended portions of vegetables, salad, and fruit each day. The Department for Education's COVID-19 guidance emphasized the importance of providing hot lunches to ensure all students receive at least one hot meal daily	Not reported	ethics and time-restraints: "Due to ethics requirements and the rapid response time required to collect the data, it was only possible to obtain views from students aged 16– 18 years"	Not reported	Not reported
52	Santilli et al. 2011	Proud "I am proud to be a part of this project because my family actually suffers from diabetes... I am proud because I see the potential in each community, and I hope that we start a chain reaction to make people look around and see what is wrong and to take action; that we are trying to break this cycle that we are in; to be	The interns also produced a documentary. A film production team affiliated with The Color of Words followed teams and interviewed residents about creating a healthier city. The film, 3000 Miles (http://www. vimeo.com/12392274), is a powerful advocacy tool for CARE.<br><br>CARE has initiated a Healthy Corner Store pilot program in several stores adjacent to	Not reported	Availability of geographical information system software was a challenge; low-cost software could not accommodate data complexity and thus limited youth involvement in mapping the collected data.<br><br>ArcView 9.2 (ESRI) software produced attractive and useful maps but required user expertise.	"The bottom line is that youth can't do it alone. We need people of all ages, of all types, to come together as equals and listen."	"We overcame mistrust and obtained access to businesses by hiring youths who came from and reflected the racial/ethnic makeup of the communities mapped."<br><br>CARE provided preliminary and on-the-job training and mentorship to prepare interns for conducting asset mapping and ensuring data quality, as well as to

		IMPACT		PROCESS			
**No.**	**1st Author + year of publication**	**Individual outcomes of adolescent participation**	**Policy related outcomes at a community, national or global**	**Barriers reported by adolescents**	**Barriers reported by adult researchers**	**Enablers reported by adolescents**	**Enablers reported by researchers**
		a leader and start something beautiful."	schools and intends to scale up citywide.<br><br>"We are working with the city to develop a comprehensive food policy to address access to healthier foods." (p. 2209-10.) - no reference				increase research capacity. This approach imparted professional skills in a city where research is a major industry and nurtured a cadre of future leaders who understand community engaged research.<br><br>Interns were paid \$8 to \$9 per hour (25 hours/week).<br><br>"User-friendly, open-platform geographical information system software is needed so that youths and other community research partners can access and contribute to the mapping process for their own advocacy purposes."<br><br>"Key elements to the project were partnering with youth leadership development organizations, focusing on youth capacity building and mentorship, supporting youths through safe data collection with field captain supervision and team deployment, employing a comprehensive advanced outreach strategy, and using handheld computers to facilitate efficient data collection and management." (p. 2207)<br><br>"We were able to efficiently and effectively conduct scientifically rigorous mapping while gaining entry into some of New Haven's most research-wary and sceptical neighborhoods"
53	Shah et al. 2023<br><br>Foley et al. 2017<br><br>Shah et al. 2017	Peer leaders reported their involvement with the SYV programme as positive, with 68% rating it as 'very valuable'. Skills gained by the peer leaders included teamwork (90%), communication (85%), leadership (77%) and confidence (65%). 25% of peer leaders indicating they also developed advocacy skills. Students from all seven schools presented their actions to receive feedback and expressed confidence that their actions were sustainable.<br><br>Foley: improved nutrition outcomes: "There were significant increases in the proportion of Year 10 peer leaders (n = 415) who reported eating ≥2 serves fruit/day fruit from 54 to 63% ($P < 0.01$); eating ≥5 serves vegetables/day from 8 to 12% (P <	One school has since installed a total of five water refill stations.<br><br>The SALSA programme has demonstrated capacity to be translated internationally with trials undertaken in the Middle East (Jordan) and China.2 The programme has been cited by the United Nations Inter-agency Technical Task Team on Young People as 'good practice in adolescent and youth programs in the Middle East and Africa (Shah 2017)	The most common challenges peer leaders encountered while developing and implementing their actions were communicating the activity to other students (36%) and lack of time (29%)	One school reported that their intervention was meant to be implemented the following year but was disrupted by COVID-19.<br><br>"While we invited all 16 schools to participate in SYV, only the first 7 schools who responded were invited, due to the limited capacity and availability of the facilities where the training occurred." (p.551)	Not reported	"The School Action Plans developed by students were realistic and achievable, with the potential to positively impact the whole school community." (p.551)<br><br>Important to strengthen partnerships with external organisations that can contribute funding towards the actions, for example, local medical associations.<br><br>Effective communication strategies and building advocacy skills should be included in future workshops.<br><br>The SYV programme showed that providing peer leaders with ownership of their actions was successful in creating healthy school environments, while building leadership, teamwork, communication skills and confidence in adolescents.

		IMPACT		PROCESS			
**No.**	**1st Author + year of publication**	**Individual outcomes of adolescent participation**	**Policy related outcomes at a community, national or global**	**Barriers reported by adolescents**	**Barriers reported by adult researchers**	**Enablers reported by adolescents**	**Enablers reported by researchers**
		0.01); and drinking <1 cup/day of SSBs from 56 to 62% ($P < 0.01$). Change in ≥60 min MVPA participation/day depended on gender ($P < 0.01$): Boys increased 14% while girls decreased −2%."					
54	Shaw et al. 2023	Not reported	Adolescent support of policy to promote healthy eating - "Adolescents in this study expressed expectations for the government to help people their age stays healthy and demonstrated an awareness of existing strategies that are intended to encourage healthier dietary behaviours. " (p.9)<br><br>Implications for planned policy implementation:<br>"Participants in this study described meal deals (sandwich, snack and drink promotions) as a commonly purchased promotion among people their age because they provide ready-to-eat food in a small-scale, affordable promotion. The promotions regulation, due to be implemented in October 2025, excludes these types of deals because they are categorized as ‘lunch options for adults to consume on the go"<br><br>"study suggests that the UK Government restrictions on advertising, now scheduled for introduction in January 2025 are important because many participants reported seeing adverts for unhealthy foods in online content such as videos and social media feeds that directly targeted their age group... these advertising restrictions will be based on HFSS foods defined using the UK's Nutrient Profile Model (Department for Health, 2011). However, no limits will be placed on the advertising of food branding provided no specific HFSS products are identifiable in the advert; for example, the globally recognized KFC brand can be included in an advert as long as no HFSS products are shown" (p.10)<br><br>loopholes in convenience stores e.g. "Government regulations to restrict the prominent placement and multibuy promotions could have a beneficial impact on adolescents’ food choices. However, these regulations exclude small retailers with under 50 employees or stores that are smaller than 185.8 m2 (Department of Health and Social Care, 2021b). These small out-of-scope stores can continue to market	Not reported	Not reported	Not reported	Not reported

		IMPACT		PROCESS			
**No.**	**1st Author + year of publication**	**Individual outcomes of adolescent participation**	**Policy related outcomes at a community, national or global**	**Barriers reported by adolescents**	**Barriers reported by adult researchers**	**Enablers reported by adolescents**	**Enablers reported by researchers**
			unhealthy foods and it is therefore possible that adolescents will not receive the full benefit offered by these policies. This gap in the policy coverage may potentially widen inequalities as populations known to have poorer diets (i.e. young people, older adults and socioeconomically disadvantaged families) often rely on convenience stores to purchase food"				
55	Sikic et al. 2012	gained valuable work experience from Fresh Producers. Being part of a team and working in a sales environment were identified as important factors, and students self reported improved nutrition and professional skills e.g. interpersonal, time management, and team building skills.<br><br>"Professional training was also an important part of the program, as the majority of students at all three schools reported gaining real-world job skills, including interpersonal and organizational skills and an improved ability to work as part of a team"	Not reported	Students observed that some teachers lacked familiarity with Fresh Producers' goals and the integrated curriculum, leading to insufficient faculty support. At the traditional high school, students in the business academy faced challenges in finding customers and suggested more student-friendly marketing for the produce. At the continuation high school, students highlighted the absence of an organized financial reimbursement system, making it hard to track earnings and leading to irregular distribution of gift cards, which all focus group participants identified as the primary issue.	challenges reported at each site differed, reflecting differences in how the programs were organized at each school. "At the charter school, faculty involvement in an ambitious integrated curriculum was variable and inconsistent. At the traditional high school, marketing fresh produce to other students was difficult to organize. At the continuation high school, the financial part of the program was the great motivator but also the greatest challenge, as students said they were not compensated adequately or in an organized manner. "	Not reported	financial incentives: Students were paid for their work with gift cards at the continuation high school, earning an income was a major motivation for all the students in the focus groups.<br><br>"improved coordination of the program could overcome some of these challenges, possibly by providing more professional development and resources for participating teachers, and on-site support to teachers and students, perhaps in the form of a youth coordinator. "<br><br>Partnerships with local organizations such as 4-H clubs or community health agencies could provide additional expertise and resources
56	Spencer et al. 2019	Not reported	Not reported	The main barriers identified were:<br>- Lack of Feedback Opportunities: Students felt there were insufficient ways to provide feedback on school food.<br>- Doubt About Impact: There was scepticism about whether their input would be taken seriously or make a difference.<br>- Busy Schedules: Many students were too busy to engage actively in food-related changes, despite a desire for more involvement.	Not reported	Not reported	3 meetings were held with the youths: an initial training workshop and 2 follow-ups focus group. initial 4-hour workshop included an introduction to the project, developing familiarity with ethical procedures for photo-taking, instruction from a photographer, and time for practice.<br><br>Most youth used their mobile phones to take photos but 3 borrowed camera from their community organisation
57	Tiedje et al. 2014	Not reported	Not reported	Not reported	"Not all participants contribute equally in all focus groups, potentially leaving shy or withdrawn participants out"<br><br>"not all FGs were conducted by native language speaker moderators. This resulted in longer sessions for one Mexican and two Cambodian FGs, contributing potentially to loss in time to go deeper into the different topics."	Not reported	"Adults and adolescents were interviewed separately to encourage open conversation that was developmentally appropriate"
58	Tsui et al. 2012	"Youth often reported being surprised by how receptive local residents and food store owners were to their ideas and perceived these interactions as empowering"<br><br>"Youth and staff reported several	The HEP curriculum serves as a replicable tool for health departments to engage young people to engage in food activism.<br><br>The Healthy Value Meal program worked with a local Latino restaurant and pizza shop to promote a 'healthy option' meal,	Not reported	Working with youth organisations that don't understand the HEP rationale and are short-staffed<br><br>Youth organisations where the mission was more individual-focused rather than community-minded	Not reported	Active input and investment from youth organisation partners, including resources and staffing<br><br>Working with youth organisations that shared the values of the HEP, particularly activism

		IMPACT		PROCESS			
No.	1st Author + year of publication	Individual outcomes of adolescent participation	Policy related outcomes at a community, national or global	Barriers reported by adolescents	Barriers reported by adult researchers	Enablers reported by adolescents	Enablers reported by researchers
		positive outcomes from the action projects. These included more active discussion of food environments within participants' peer group, family and community; greater awareness of the influence of economic factors and policy on local food options; and a realisation on the part of many participants that reducing food-related health problems required community and municipal action as well as individual change"	including a healthier pizza and a chicken salad sandwich, each with a bottle of water		Time of year and duration of the HEP programs offered, there was some competition with other compensated programs/ employment opportunities available to youth, particularly in Summer<br><br>When the curriculum components were not adequately matched to the developmental level of participants		Having a stable contact person with each youth organisation with whom to organise the programs and workshops<br><br>Having strong youth-staff rapport and relationships<br><br>taught skills to help youth become advocates/ community leaders
59	Utter et al. 2010* related study<br><br>Utter et al. 2011	Students involved in the student health councils received training in nutrition, activity and health and opportunities for building leadership skills and planning intervention activities.	"The student health councils created and maintained several successful interventions within their schools"<br>Installation of water fountains at schools.<br>The student health councils (who ran the Living 4 Life project in their schools) contributed to events including the 2006 WHO Regional Committee for the Western Pacific region to help the New Zealand government launch new initiatives for obesity prevention.<br>There was an implementation of a school-wide nutrition policy.	Not reported	3 schools were unable to make changes to school canteens as they were run by separate businesses.<br>securing funding to sustain initiative.<br>breakfast club - "The burden of responsibility is ,at times, too much for students."<br>healthy canteen: "Contractual obligations and profitability of the privately run canteens can hinder the work in this environment. Major improvements came about when national legislation was introduced requiring the foods provided at school to be healthy. This legislation was repealed less than a year later"	Not reported	School administration staff who are very supportive of the initiative<br>Providing opportunities for meaningful participation, quality relationships, a strengths-based approach, and creating opportunities for student training and development.<br>Barriers to participation in the student health councils such as transportation and cost were removed.<br>strengths-based approach
60	Vecchiarelli et al. 2006	Not reported	Not reported	Not reported	Not reported	Not reported	Not reported
61	Watts et al. 2015	Not reported	"the emphasis placed by these adolescents was on all the "junk food" that was available, likely reflecting the high visibility, value and promotion of these foods, and/or their salience to these adolescents... We know from principal-reported surveys that BC schools reduced the availability of less healthful foods in 2011e12, yet many schools continued to offer less healthful options" (p. 364-5).	Not reported	Not reported	Not reported	Adolescents were provided with a digital camera, instructed on its use, and engaged in discussion on the ethics and sensitivity of this research technique
62	Wojcicki et al. 2006	Not reported	The success of this program led to major changes<br>in nutrition standards throughout the district. Expanded incorporation of healthier meal choices in the revamped SFUSD nutrition standards further affected and improved the food choices in the snack bars as well as the federal school lunch program menus.	Not reported	Concern for lost revenues led to initial reluctance by the School Nutrition Services to implement any changes	Not reported	Not reported
63	Wright & Nault 2013	"Youth continually attributed personal eating habits and the food environment to issues of personal choice. They had difficulty seeing correlations between diet-related health disparities, differences in food cost and availability, and differences	Not reported	Not reported	They had difficulty seeing correlations between diet-related health disparities, differences in food cost<br>and availability, and differences in the local environment that might shape life behaviours compared to personal choice	Not reported	youth paid $100 for their participation<br><br>Parents/guardians granted participation permission by signing consent forms.<br><br>Training sessions e.g. curriculum

		IMPACT		PROCESS			
**No.**	**1st Author + year of publication**	**Individual outcomes of adolescent participation**	**Policy related outcomes at a community, national or global**	**Barriers reported by adolescents**	**Barriers reported by adult researchers**	**Enablers reported by adolescents**	**Enablers reported by researchers**
		in the local environment that might shape life chances. Our experience with the lack of civic responsibility among youth aligns with other studies that demonstrate declining levels of civic participation (Galston, 2003). Such perceptions may be due to an over-emphasis in civic curricula on freedoms and rights to the exclusion of social obligations (Avery & Simmons, 2001)."			"Our experience with the lack of civic responsibility among youth aligns with other studies that demonstrate declining levels of civic participation (Galston, 2003). Such perceptions may be due to an over-emphasis in civic curricula on freedoms and rights to the exclusion of social obligations"		
64	Yoshida et al. 2011	"This project made me more aware and passionate about changing what's in our community."<br><br>Overall, through policy work, youth believed that they gained confidence and a great sense of pride because their voices were heard:<br>"It'll give me experience for the future – to change the community, it gives me pride and dignity."<br><br>Experience in public speaking helped them develop research and with organisation and writing skills. Youth felt a sense of accomplishment from sharing their opinions, having their voices heard and being acknowledged for their work.<br>Built leadership confidence	Youth helped shape their school wellness policies and had opportunities to talk with local and state legislators.<br><br>Youth in one community collaborated successfully with their local government on a moratorium against drive through fast-food restaurants.	Not reported	Not reported	"This project made me more aware and passionate about changing what's in our community."<br><br>"It'll give me experience for the future – to change the community, it gives me pride and dignity."	Participants received a healthy snack and a $20 gift voucher.<br><br>Several youth participated in a statewide leadership program which provided training in leadership and environmental and/or policy change strategies and an opportunity to network with their peers on a statewide level, which allowed them to share their concerns and feel supported by one another
65	CO-CREATE Project Baillergeau et al. 2024 Broeer et al. 2023 Budin-Ljosne et al. 2023 Klepp et al. 2023	see below	see below	see below	see below	see below	see below
65a	Baillergeau et al. 2024	Not reported	Not reported	Some adolescents reported against policy interventions to restrict advertising unhealthy food products and that it was up to the individual and/or their parents.	being invited to explore their own perception of what matters with childhood obesity led some adolescents to views that were less in line with systemic assumptions and led to debate across youth alliance groups and members e.g. stigma on overweight.<br><br>The COVID-19 pandemic restricted in person workshops for the Dutch Youth Alliance and were unable to develop a policy proposal	Referring to existing policies and how these measures were evaluated helped adolescents creating new policy ideas. "The research done [by adolescents] about other policies and initiatives, already in place, was crucial to further develop and strengthen the policy ideas. The Q&A sessions [with experts the young people invited], that helped to put in practice the conversational interview training, were also	By analysing youth participation in the EU CO-CREATE project, it highlights that effective democratisation in health promotion requires incorporating diverse and potentially conflicting forms of knowledge through inclusive risk negotiation.<br><br>Some young people were inspired by discussing system maps made by other young people as part of CO-CREATE.

		IMPACT		PROCESS			
No.	1st Author + year of publication	Individual outcomes of adolescent participation	Policy related outcomes at a community, national or global	Barriers reported by adolescents	Barriers reported by adult researchers	Enablers reported by adolescents	Enablers reported by researchers
						very important to give new and different insights to the youth alliance members, helping them to define some aspects of their policy ideas. [Facilitator Survey May 2020]" (p. 9).<br>Drawing on peer knowledge to enhance their knowledge of obesogenic environments.	
65b	Broeer et al. 2023	CO-CREATE process evaluation questionnaire was developed to measure adolescents' readiness for action and attitudes toward obesity prevention	Created policy proposals - Adolescents focused on local obesity challenges at school, using their own experiences and participatory research. Simultaneously, they were encouraged to address broader, complex factors contributing to obesity when designing policy proposals. This approach acknowledges the challenge highlighted by the Lancet Commission on Adolescent Health and Wellbeing: balancing local engagement with addressing systemic issues. CO-CREATE navigates this by fostering collaborative learning and helping youth to balance practical activities with a broader perspective on obesity.	Not reported	Not reported	Not reported	Fieldwork preparation activities e.g. recruiting interested adolescents, identifying gatekeeper organisations<br><br>Youth Alliance Activity Handbook was created based on YPAR principles, project goals, and initial fieldwork by CO-CREATE researchers and PRESS, a youth organization skilled in promoting youth participation. They created a series of activities for adolescents and staff to use in the Alliances as a guide.<br><br>Each Alliance included at least two adult staff members: a facilitator from the CO-CREATE consortium and a younger cofacilitator from a local youth organization. The cofacilitators acted as a bridge between the research team and the adolescents, helping to transfer knowledge and sustain activities. They attended a 2-day training workshop to learn about the project, and key research skills such as assisting with recruitment, taking meeting minutes, vlogging and using social media<br><br>Capacity building activities given to adolescents e.g. advocacy training, budgeting, and policy formulation.<br><br>Anonymous feedback forms used for evaluation purposes e.g. building process and it's outcomes.<br><br>Prevented ethical issues by a "two-stage procedure for the collection of informed consent from youth, written group agreements and codes of conduct, youth and staff training in ethics and flexibility in the organization, and timing of meetings with youth. Alliance members were presented a draft code of conduct in which safety, non-discrimination, and other conduct rules were proposed." (more details in Budin-Ljøsne 2022 study)

		IMPACT		PROCESS			
**No.**	**1st Author + year of publication**	**Individual outcomes of adolescent participation**	**Policy related outcomes at a community, national or global**	**Barriers reported by adolescents**	**Barriers reported by adult researchers**	**Enablers reported by adolescents**	**Enablers reported by researchers**
65c	Budin-Ljosne et al. 2023	"though we did not systematically assess which benefits were brought to the youth through their participation in the project, we observed that the young people developed new awareness of systemic factors influencing obesity, acquired decision-making and observation skills, and learned to use new tools such as Photovoice... We also experienced that some young people were accepted on higher school levels or finished their follow-up education sooner than expected as a direct result of their participation in the Youth Alliances. Others decided to pursue their work with local authorities after their participation in the project had ended to implement their policy ideas"	Not reported	Continued engagement: Some young people reported that they felt less engaged in the activities after a while and would rather focus on their school tasks	meetings and activities had to be conducted online following the outbreak of COVID-19 pandemic and may have influenced lower engagement e.g. digital burnout. Due to limited resources, it was not possible to provide digital access to those lacking it. Some youth were unable to participate because they didn't have access to software/ necessary equipment. Youth required more prompting during online meetings and seemed less willing to participate in discussion<br><br>the project staff later discovered that one school had incorporated participation in the project as part of the students' curriculum which went against their 'voluntary participation' consideration. This matter was resolved and students reminded they could withdraw at any time.<br><br>Conflicting Obligations: Many struggled to balance schoolwork, exams, family responsibilities, and transportation issues.<br><br>Timing Issues: Meetings held after school often led to fatigue and lack of focus, and some youth could not attend due to other commitments.<br><br>Engagement Challenges: Some participants did not complete assigned tasks between meetings, potentially due to either time constraints or lack of interest.<br><br>Exam Pressure: Those in their final year of upper secondary school faced particular difficulties due to the high demands of exam preparation and additional educational challenges, such as language barrier. Disengagement observations by project staff e.g., hiding face under hoodie or playing with phone), complained that the project was boring, that the meeting was too early in the morning, participated in the work only when the teacher was present, or in some occasions dropped out.<br><br>Researchers reported they struggled to accommodate the demands of youth regarding different meeting times due to restrictions on the availability of meetings rooms in the schools, and clashes with	Not reported	The young people received support from the CO-CREATE staff and co-facilitators, which were young people recruited from local youth organizations (Bröer et al.)<br><br>Held meetings either before or after school hours, or within school hours/scout meetings depending on youth preferences and other obligations.<br><br>Transition to online meetings meant some new young people could join remotely. To keep them engaging, they added quizzes and breakout rooms for smaller group discussions<br><br>To address potential issues of data privacy and confidentiality, the project partners provided youth, facilitators, and co-facilitators with training in ethics and management of written and visual information.<br><br>To prevent power imbalances in the Youth Alliances, young people were asked to outline written group agreements describing ground rules for interaction with each other such as respecting others' opinion, giving space for others to participate or not laughing at each other.<br><br>For Dialogue Forums, similar written agreements were implemented that stakeholders had to comply with, and a balanced representation (50/50) of youth and stakeholders in the meetings was required

		IMPACT		PROCESS			
No.	1st Author + year of publication	Individual outcomes of adolescent participation	Policy related outcomes at a community, national or global	Barriers reported by adolescents	Barriers reported by adult researchers	Enablers reported by adolescents	Enablers reported by researchers
					exams<br><br>Enabling equal levels of participation in larger groups was however challenging as some young people were more vocal and eager to take the lead than others. Some tension<br><br>Dialogue forum: "discussions with youth participants and moderators after the Dialogue Forums did demonstrate that at times, select stakeholders dominated the conversation, tried to convince youth to adapt or modify their opinion, and responded in condescending ways to young people's questions." (p.6)<br><br>Recruiting boys - "We discussed with project partners and youth already participating in the project how to recruit more boys but did not manage to identify successful strategies" (p. 6)<br><br>Ethical challenges:<br>Engaging youth throughout research design "may mean that renewed ethics approval for each new research cycle is required"<br>"“ethics” had some shortcomings and did not fully support participatory approaches under which youth are encouraged to endorse a role of research collaborators (who develop policy ideas) rather than research participants"		
65d	Klepp et al. 2023	Not reported	Key factors for youth engagement were: (i) safe and supportive spaces, (ii) passion and interest, (iii) using their voice, (iv) power dynamics, (v) accessibility and (vi) awareness.<br><br>Dialogue tool prototype was then tested with young people in various workshop and conference venues such as the EAT Stockholm Food Forum and UNICEF State of the World Children's Report launch."	Not reported	"There are several inherent risks in conducting such a large-scale research project with adolescents across several countries. These include, but are not limited to, recruitment challenges and involvement of diverse population groups, securing genuine involvement and treating adolescents as equal partners, and continued engagement over an extended time period."<br>Covid-19 - transitioning online	Not reported	system mapping - To generate the CLDs with the adolescent participants in the GMB sessions, we used STICKE software. They then merged each countries maps together - reported in Savona et al. 2021<br><br>Each Alliance had one or two co-facilitators: a young adult bridging the gap between young members and the (academic) adult facilitators.<br><br>"Various measures were integrated to ensure youth were protected yet still empowered to play a leading role in the Dialog Forum. Such measures include a 1:1 ratio of youth to adult participants, providing young people the opportunity and training to moderate, assessing stakeholders through a risk assessment table, and other measures"<br><br>"Creating a safe space where youth and adults can discuss ideas and cocreate

		IMPACT		PROCESS			
No.	1st Author + year of publication	Individual outcomes of adolescent participation	Policy related outcomes at a community, national or global	Barriers reported by adolescents	Barriers reported by adult researchers	Enablers reported by adolescents	Enablers reported by researchers
							solutions as equals is central to promoting meaningful youth engagement and reinforcing young people's role in policymaking."<br><br>"The project integrated mechanisms at all stages of the research to address and mitigate potential ethical challenges. Mechanisms, for instance, included a thorough process to inform adolescents about the research objectives and methods, adolescent training in advocacy and research methods, tools for stakeholder selection, and requirements that stakeholders commit to respect the views and opinions of adolescents."
66	Banik et al. 2023<br><br>*CO-CREATE, separate study	Not reported	Young people find the policy solutions from the NOURISHING and MOVING frameworks relevant to them within their local environments and identified current policies and opportunities to introduce policies to create supportive environments that promote healthy living. These policies include creating healthy retail and food environments, limiting unhealthy food advertisements, promoting physical activity through supportive structures, and improving transportation infrastructure to encourage active lifestyles.	Not reported	Limitation - the coding of policy themes was conducted by researchers only (without involving youth), and the findings were not validated by the members of the Youth Alliances.	Not reported	Photos were taken on adolescent smartphones or a camera provided by research team.
67	Conway-Moore et al. 2023<br><br>*CO-CREATE, separate study	Not reported	proposed policy ideas reviewed in this study provide evidence of the desire among young people to either lead or be directly involved in the processes that shape their diet and physical activity.	Not reported	limitation - post hoc analysis of the youth-generated policies using the ILF, evidence of the effectiveness of different levels of system intervention was not part of the Youth Alliances' original policy development.	Not reported	"Youth Alliance facilitators across participating countries also prioritized opportunities for capacity-building among the Youth Alliance members as a means to contribute to their idea development. This included organizing meetings with local public health officers where the Youth Alliance participants could learn more about existing health policies and initiatives happening in their communities, as well as connecting the Youth Alliance participants with key stakeholders (e.g., local and national-level policymakers, private sector business owners, and representatives from non-governmental organizations)" (p.2)
68	Herstad et al. 2023<br><br>Grewal et al. 2023<br><br>*CO-CREATE, separate study	Results from Herstad et al. 2023: Involving youth in co-creating policies to prevent obesity may increase adolescents' readiness for action (i.e. ways of expressing political voice, competence for civic action, advocacy outcome efficacy, knowledge of resources) and promote a shift in adolescent conceptualisation of obesity from individual perspective to a societal	Not reported	Not reported	transitioning to online meetings due to COVID-19 may have influenced drop in participation in the Youth Alliance activities	Not reported	Web-based surveys have several benefits, such as shorter transmitting time, lower delivery cost and less data entry time, and it may be a more efficient way to reach adolescents.

		IMPACT		PROCESS			
No.	1st Author + year of publication	Individual outcomes of adolescent participation	Policy related outcomes at a community, national or global	Barriers reported by adolescents	Barriers reported by adult researchers	Enablers reported by adolescents	Enablers reported by researchers
		responsibility and drivers of behaviour.<br><br>Increased Readiness for Action: the Alliance members scored higher than the comparison group on three factors and a single item: "ways of expressing political voice, "competence for civic action," "advocacy outcome efficacy," and "using social networking platforms to discuss a social issue." See table 2<br><br>Alliance members scored significantly higher than the comparison group on "advocacy outcome efficacy" (b = 0.3, 95% CI = 0.0, 0.6 p=<0.04) and "using social networking platforms to discuss asocial issue" (b=0.6,95%CI=0.1,1.0 p=<0.02)					
69	Nesrallah et al. 2023<br><br>Ulloa et al. 2023<br><br>*CO-CREATE, separate study	Not reported - "Measuring outcomes and impacts of Dialogue Forums proved to be challenging." (p.10)	Ulloa et al. 2023:<br>main outcomes from dialogue forum included young people getting connected with stakeholders after the Dialogue forum, however there were few tangible outcomes and short-term impacts. Portuguese stakeholders inviting Youth Alliance to present its policy ideas to City Hall and a Norwegian politician asking the minister of health to consider drafting a bill to ban ads of unhealthy food and drinks on digital platforms aimed at children under 18 years. Multiple prototypes were tested in 53 countries with more than 200 participants.<br><br>Co-Create Dialogue Forum Tool has now been used UN meetings, translated in 10 other languages and freely available: https://eatforum.org/initiatives/co-create/digital-dialogue-forum-tool/	Ulloa et al. 2023: vulnerability sharing personal stories with policy makers. Invitation - rare to be invited in policy decision making. If they are invited the purpose of the invitation is unclear. Preparation - they want to level the playing field and need time to prepare. Want to be treated as equals. Meeting/ dialogue: being patronized, invited to participate but not heard, hard to talk real beyond slogans, limited action, mainly talking.	extracted from Nesrallah et al. 2023: "the desire to promote youth autonomy in decision making was at times challenged by the desire to safeguard young people and the public health policy discussion. Ultimately, the suggested measures and tools in the framework aim to balance empowerment and safeguarding. However, providing young people with further education and training on how to identify, address, and eliminate COIs could create stronger participatory processes."<br><br>"Although efforts were made to extend engagement to both digital surveys and in person interviews, we know that when youth are not given sufficient time to engage in participatory processes, they risk being tokenistic. In an effort to strengthen the relevance of the COI framework, future consultations with youth should take place." (p.10)<br><br>Barriers in general identified from background section: "First, on the basis of age, youth face unique risks such as lack of civic engagement opportunities, lack of information on and voice in decision-making processes, income poverty, transitional development period, and other mental health risk factors (antisocial peers, bullying, etc.)."<br><br>"Second, on the basis of power, when engaging in dialogue with youth, adults can inadvertently exert their power and	Nesrallah et al. 2023: "if adults want to include youth in their work, then they have to meet them in a completely informal, unconventional way. Ideally, it should be the adults who break their routines to meet youth, not the other way around."<br><br>Ulloa et al. 2023: "little things make us feel valued - like the informal 5 minutes before, during or after the meeting". Informal and personal communication can be powerful and efficient than the formal routes. Knowing they aren't alone makes it easier to put themselves out there. They want to leave meetings with action points. If there's no follow up, be straight forward to avoid them left wondering.<br><br>They developed 7 design principles: 1) for youth by youth, 2) youth are plural, 3) invest time in the "unproductive bookend" moments, 4) make introductions personal, 5) everyone's perspective matters, 6) focus on desired outcomes, 7) focus on doing (not only talking)	extracted from Nesrallah et al. 2023: Actions to put principles of engagement into practice (p. 6-7)<br>"1. Respectful: Share a code of conduct with all participants ahead of the Dialogue Forum, with a particular emphasis on conduct that promotes mutual respect.<br>2. Inclusive: Meet young people where they are and organize Dialogue Forums in or near schools, local community centres, youth clubs, and other areas that reduce barriers to participation.<br>• Work with grassroots organizations that represent young people from various communities to engage said youth.<br>• Invite participants from a variety of sectors and at varying levels (local and national-level stakeholders, small-medium sized enterprises, multinational companies, etc.).<br>3. Voluntary: Communicate the voluntary nature of the dialogue to all participants at the beginning of the session.<br>• Provide youth with guidance on how to cease participation in the Dialogue Forum if they wish.<br>4. Open and transparent: Share clear roles, responsibilities, and expectations with all participants ahead of the dialogue.<br>• Provide youth with information about their right to express their views freely and will be encouraged to share their perspectives.<br>• Require all stakeholders to sign a consent form which outlines that by

		IMPACT		PROCESS			
**No.**	**1st Author + year of publication**	**Individual outcomes of adolescent participation**	**Policy related outcomes at a community, national or global**	**Barriers reported by adolescents**	**Barriers reported by adult researchers**	**Enablers reported by adolescents**	**Enablers reported by researchers**
					influence to shift overall consensus, and youth can be coerced into expressing views that go against their wishes or beliefs. As a result, youth may leave feeling exploited or unable to express their views." (p.3)<br><br>Ulloa et al. 2023: "Measuring outcomes and impacts of Dialogue Forums proved to be challenging. Through the development of the conflicts of interest framework,21 it was deemed high-risk to facilitate direct youth and adult stakeholder engagement post-dialogue."<br><br>"During the Dialogue Forum sessions, we felt a safe environment. Since the tool is designed to establish an equal discussion and an equal seat at the table, the stakeholders talked to us with a different attitude. They were trying to learn from us, as much as we were learning from them. After all, this is what intergenerational discussion is about. Youth cannot change the world on their own, and politicians cannot make everyone happy without putting themselves in everyone's shoes. So, if a policy idea affects youth, youth should be consulted."	"Since our feedback was heard, we can confirm that the tool was made for youth, with youth." (Ulloa et al.p.12)	agreeing to participate, they consent to putting the primary public health interest of the initiative ahead of any conflicting financial, academic, or personal interests. Stakeholders also consent to abiding by communication and exploitation guidelines regarding their involvement in CO-CREATE to mitigate any misrepresentation of their involvement.<br>• Communicate the purpose, process, and anticipated outcomes of the dialogue to all participants involved to increase transparency. Organizers must however be cautious and mitigate unrealistic expectations of the outcomes and impact of the forum.<br>5. Accountable:<br>• Share outcomes of the dialogue with all participants through a summary report.<br>• Encourage all participants to follow-up on actions and commitments made at the Dialogue Forum, published online.<br>• Give participants the opportunity to provide clear feedback on the Dialogue Forum process through an evaluation survey.<br>• Wherever possible, give youth the opportunity to participate in follow-up processes or activities, specifically related to advancing their policy ideas.<br>6. Safe and sensitive to risk: Inform youth of their right to be protected from harm, right to withdraw, and know where to go for help if needed.<br>• Clearly communicate safety measures that adult participants must abide by, such as not being alone with a young person in a room or closed space.<br>7. Empowered: Provide youth with the opportunity and capacity building training to act as moderators of the discussion.<br>• Encourage participants to take action and collaborate after the Dialogue Forum."<br><br>Stakeholder risk assessment to support the mitigation and management of COIs see table 2. page 8 + It was adapted from the World Obesity Federation's Financial Relationship Policy and WHO Tool to Safeguard Against Conflicts of Interest in Nutrition Policies and Programmes.<br><br>"The code of conduct sets standards for participant behavior during the Dialogue

		IMPACT		PROCESS			
**No.**	**1st Author + year of publication**	**Individual outcomes of adolescent participation**	**Policy related outcomes at a community, national or global**	**Barriers reported by adolescents**	**Barriers reported by adult researchers**	**Enablers reported by adolescents**	**Enablers reported by researchers**
							Forum, and informs them of the values, rules, and ethics of the dialogue."
70	Savona et al. 2021<br><br>Savona et al. 2023<br><br>*CO-CREATE, separate study	2023 paper: "Similarly to the “Our Voice” approach to citizen engagement in research, empower residents to assess how their communities impact their well-being—taking the focus away from individual-level behaviors—and to identify actions for improving citizens' health"	Not reported	Not reported	GMB can take time and may have deterred participants from signing up due to competing priorities/ busy schedules<br><br>Navigating admin and ethical hurdles	2023 paper: "Our experience was that the GMB sessions ran smoothly and openly, with no sense of stigma or awkwardness around the topic of body weight and little dropout between GMB sessions. With skilled facilitation, GMB can provide opportunities for open discussion on delicate topics such as body weight"<br><br>"The sessions switch throughout between exercises that are “convergent” and “divergent,” that is, working as individuals then as a group, then as individuals, through the different phases of creating the CLD (see Table 1). This ensures that even people who may be disinclined to speak out in a group setting are still ultimately represented on the CLD; the process builds a sense of shared vision and a group product in the diagram; it also helps incorporate and smooth out any disagreement that may arise. Again, we found that our adolescent participants were engaged, with high repeat attendance at second sessions."<br>Experienced facilitators	GMB with young people demonstrates a workable way of involving young people in research and a starting point for finding ways to develop effective policy actions that resonate with them (p.395)<br><br>2023: Researchers took part in a 5-day training course to learn how to recruit for, facilitate, and use results of GMB sessions to generate a causal loop diagram.
